# A highly recombined, high‐density, eight‐founder wheat MAGIC map reveals extensive segregation distortion and genomic locations of introgression segments

**DOI:** 10.1111/pbi.12504

**Published:** 2016-01-23

**Authors:** Keith A. Gardner, Lukas M. Wittern, Ian J. Mackay

**Affiliations:** ^1^ The John Bingham Laboratory National Institute of Agricultural Botany (NIAB) Cambridge UK; ^2^ Department of Plant Sciences University of Cambridge Cambridge UK

**Keywords:** Multiparent Advanced Generation Intercross (MAGIC), high‐density map, wheat, recombination, segregation distortion, introgression

## Abstract

Multiparent Advanced Generation Intercross (MAGIC) mapping populations offer unique opportunities and challenges for marker and QTL mapping in crop species. We have constructed the first eight‐parent MAGIC genetic map for wheat, comprising 18 601 SNP markers. We validated the accuracy of our map against the wheat genome sequence and found an improvement in accuracy compared to published genetic maps. Our map shows a notable increase in precision resulting from the three generations of intercrossing required to create the population. This is most pronounced in the pericentromeric regions of the chromosomes. Sixteen percent of mapped markers exhibited segregation distortion (SD) with many occurring in long (>20 cM) blocks. Some of the longest and most distorted blocks were collinear with noncentromeric high‐marker‐density regions of the genome, suggesting they were candidates for introgression fragments introduced into the bread wheat gene pool from other grass species. We investigated two of these linkage blocks in detail and found strong evidence that one on chromosome 4AL, showing SD against the founder Robigus, is an interspecific introgression fragment. The completed map is available from http://www.niab.com/pages/id/326/Resources.

## Introduction

Multiparent mapping populations are now being widely developed in plant species, and combine high genetic recombination with high diversity. Examples include nested association mapping populations (NAM, Yu *et al*., 2008), the Arabidopsis multiparent RIL population (AMPRIL, Huang *et al*., 2011) and Multiparent Advanced Generation Intercross (MAGIC) populations. The MAGIC approach was first advocated for crops in 2007 (Cavanagh *et al*., 2008; Mackay and Powell, 2007) and MAGIC populations have subsequently been developed in a range of species, such as rice (Bandillo *et al*., 2013), barley (Sannemann *et al*., 2015), tomato (Pascual *et al*., 2015) and wheat (Huang *et al*., 2012; Mackay *et al*., 2014; Milner *et al*., 2015; Thepot *et al*., 2015).

Multiparent Advanced Generation Intercross populations are multifounder equivalents of the advanced intercross introduced by Darvasi and Soller (1995) and are closely related to the heterogeneous stock and composite cross‐populations used in mouse genetics (Mott *et al*., 2000; Threadgill and Churchill, 2012). They are created by several generations of intercrossing among multiple founder lines leading to greater accumulation of recombination events and hence greater mapping precision. Multiple founders contribute more allelic and phenotypic diversity than captured in typical biparental mapping populations, raising the numbers of QTL that segregate and the types of trait and locus interactions that can be investigated. A MAGIC population founded from good representation of a breeder's gene pool also offers the opportunity to explore patterns of genomic diversity in that gene pool, such as identifying linkage blocks under fertility or viability selection and locating introgression fragments introduced from other species in the breeding process.

The NIAB eight‐parent winter wheat MAGIC population (Mackay *et al*., 2014) was developed in partnership with UK breeders to represent the diversity of UK wheat germplasm. Founder varieties were Alchemy, Brompton, Claire, Hereward, Rialto, Robigus, Soissons and Xi19. Here, we describe the creation and validation of a high‐density genetic map in this population, based on the Illumina Infinium iSelect 80K SNP array (http://www.illumina.com/). This is the first publically available eight‐founder MAGIC map created for wheat. We estimate the precision and accuracy of our map by reference to the wheat genome sequence and in comparison with existing wheat high‐density genetic maps. Finally, we map recombination rates, blocks of segregation distortion (SD) and blocks of high marker density, and infer the genomic locations of blocks of interspecific introgression.

## Results

### Genotyping

A total of 643 F4 MAGIC lines (assayed as F5 progeny bulks) passed stringent quality control and were used for mapping, and 20 639 SNP markers were scorable and polymorphic, compared to 25 499 markers in a comprehensive UK wheat association mapping panel (‘WAGTAIL’, 520 varieties, similarly genotyped and scored by K.A. Gardner), suggesting the MAGIC population has captured >80% of the genetic diversity of UK wheat germplasm. Fifty‐three markers with heterozygotes or missing data in the founder lines could not be used for mapping with R/mpMap (Huang and George, 2011). Of the remaining 20 586 markers, 18 750 (91%) were scored as codominant and 1836 (9%) as dominant; 664 of the dominant loci were nulls (3.2%) compared to 5.4% of single‐locus scorable SNPs showing null alleles in Wang *et al*. (2014). For codominant markers, 2.4% residual heterozygosity was observed, compared to an expectation of 2.2%. Four PCR markers were also genotyped (Appendix S1).

### Linkage map

In total, 18 601 markers were mapped across all chromosomes. The 2042 unmapped markers (Table S1) are a highly nonrandom sample: 30% are dominant (compared to 3% of mapped markers), 16% are nulls (2% mapped) and 17.5% show SD with a false discovery rate (‘fdr’) <0.01 (9.7% mapped). All unmapped markers were treated as traits and QTL mapped to the finished map, and their flanking markers were blasted against both wheat genome sequences (International Wheat Genome Sequencing Consortium, 2014, http://www.wheatgenome.org/, ‘IWGSC’; Chapman *et al*., 2015, ‘CHAPMAP’); 1118 of the unmapped markers had matching QTL and blast hits (with ≥98% identity, Table S1). From the distribution of these unmapped markers (Table S1, ‘data analysis’), both SD and translocations correlate with failure to map. About 20% of these markers were aligned to chromosome 5B or 7B, across a segregating translocation breakpoint and 12% were from chromosome 3B, which has two major SD blocks. Several large blocks of co‐localized segregation distorted markers were unmappable, including a 104‐marker block with SD against Robigus from the distal end of 4A.

**Figure 1:**
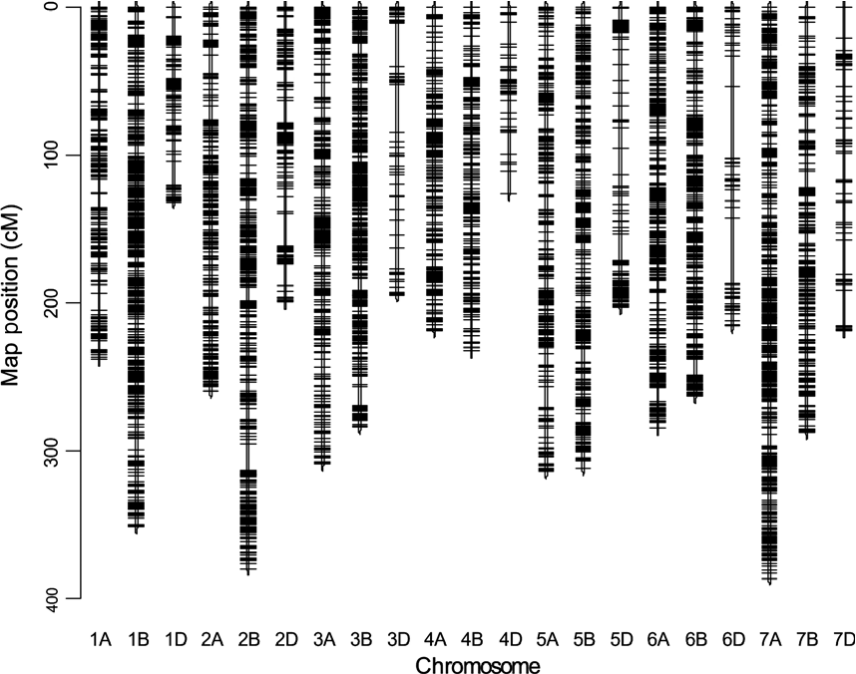
The NIAB2015 MAGIC genetic map. Short arm of each chromosome at top (0 cM).

Our NIAB MAGIC linkage map, ‘NIAB2015’, is dense and compact (Figure 1, data in Table S2). Summary statistics are shown in Table 1. The number of markers mapped per chromosome varied from 80 (4D) to 2327 (1B). About 37%, 50%, and 13% of markers were mapped to the A, B and D genomes, respectively. The map length totalled 5405 cM, with individual chromosomes ranging from 126 to 386 cM. A and B genome chromosomes together showed relatively low variation in length and were about 60% longer than D genome chromosomes. Altogether, the 18 601 markers were mapped to 4578 unique sites across the genome, with 41%, 47% and 12% of the unique sites mapping to the A, B and D genomes, respectively. The number of unique sites per cM is approximately one for the A and B genomes (0.94 and 1.02, respectively) but only 0.41 per cM for the D genome, reflecting the well‐documented lower polymorphism of the D genome of hexaploid wheat (Wang *et al*., 2014).

**Table 1:** MAGIC map summary statistics and comparison to other maps (see Table 2 for description of other maps)

	NIAB 2015	Other maps				NIAB2015 overlap^a^		
		CM2014	9KCONS	9KMAGIC	SynOp	CM2014	9KCONS	9KMAGIC
**Total length (cM)**	**5405**	**11 185**	**5192**	**3722**	**3242**			
Max chromosome length	386	743	396	300	225			
Min chromosome length	126	295	99	73	67			
Average chromo length (A)^b^	287	619	284	203	156			
Average chromo length (B)	301	552	277	201	155			
Average chromo length (D)	184	427	80	128	152			
**Total No markers**	**18 601**	**40 267**	**7497**	**4300**	**N/A** c	**15 672**	**2840**	**1663**
Max markers/chromosome	2327	3471	768	451	N/A	1906	284	162
Min markers/chromosome	80	296	38	14	N/A	67	5	4
% markers A	37	38	46	48	N/A	37	46	50
% markers B	50	46	46	45	N/A	50	46	43
% markers D	13	16	8	7	N/A	13	8	7
**Total unique sites**	**4578**	**5564**	**3010**	**1813**	**1446**	**2897**	**1436**	**966**
Max unique sites/chromo	452	387	265	202	114	234	119	88
Min unique sites/chromo	39	101	30	11	28	32	4	4
Average unique sites (A)	270	282	188	114	66	161	93.6	65
Average unique sites (B)	309	328	195	125	89	188	92.1	62
Average unique sites (D)	75	185	47	20	52	65	19.4	11
**Unique sites/cM**	**0.85**	**0.50**	**0.60**	**0.49**	**0.44**			
Unique sites/cM (A)	0.94	0.46	0.66	0.56	0.42			
Unique sites/cM (B)	1.02	0.59	0.70	0.62	0.57			
Unique sites/cM (D)	0.41	0.43	0.26	0.15	0.34			

a These comparisons only include markers variable in NIAB2015.

b (A) refers to A genome.

c >1 million contigs mapped in SynOp using GbS—see text.

For A and B genomes, chromosome map length in cM is strongly associated with the number of unique sites (adjusted *R* ^2^ 0.69, *P* = 0.000132), with a gradient of 0.53 cM/unique site (95% CI 0.32–0.74), very similar to the default minimum genetic distance of 0.5003 cM between proximal recombination fraction bins in mpMap (corresponding to a recombination fraction, rf, of 0.005). Indeed, 69% of proximal unique sites in the AB genomes are 0.5 cM apart, confirming the compactness of NIAB2015.

### Internal validations

**Figure 2:**
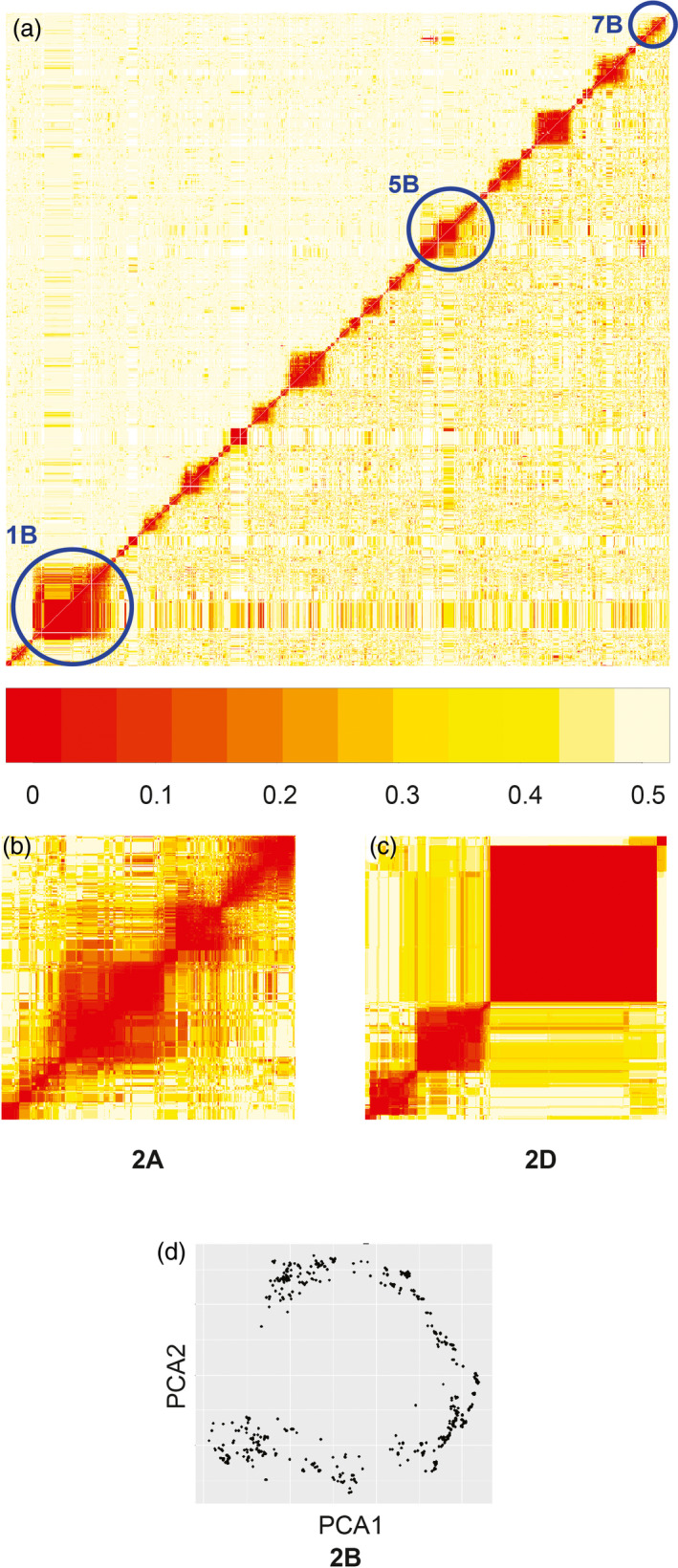
NIAB2015 map diagnostics. (a) Overall heatmap of recombination fraction matrix, with problematic chromosomes highlighted. Red to yellow = low (0) to high recombination (0.5). (b, c) heat maps for chromosomes 2A, 2D. (d) First two principal components (*x*‐axis, *y*‐axis) of recombination fraction in principal components analysis for 2B (see text).

The genomewide heatmap of the recombination fraction matrix and logarithm of odds (LOD) values for NIAB2015 shows distinctly separated chromosomes and very few off‐diagonal low‐value (rf 0–0.1) colours (Figure 2). Two exceptions are evident. Firstly, the well‐known rye introgression on chromosome 1B (Villareal *et al*., 1991; Worland and Snape, 2001) is segregating in the population (present in founders Brompton and Rialto), causing long range linkage disequilibrium (in a similar manner to Huang *et al*., 2012). During mapping, markers that are segregating within native wheat chromosomes in the region of the rye introgression will be ‘pushed to the side’ of the markers which are only segregating between rye and wheat. This is evident in our map, where we have opted to include all these markers. Secondly, there is segregation for the occurrence of a common wheat translocation between chromosomes 5BS and 7BS (Badaeva *et al*., 2007), although with unknown parental frequencies. The largest distinct group of unmapped markers are from around the translocation breakpoint.

The heat map for 2A (Figure 2) is typical for a cleanly mapped AB genome chromosome with only the centromeric region being relatively poorly defined. The heat map for 2D (Figure 2) is typical of D genome chromosomes with blocks of tightly linked markers separated by tracts of low variation, although our MAGIC map has high within‐block recombination. We also used principal component analysis (PCA) of the recombination fraction matrix to qualitatively assess whether chromosomes were well ordered. Figure 2 shows the first two principal components for chromosome 2B, demonstrating a horseshoe shape characteristic of a successfully mapped chromosome (Cheema and Dicks, 2009; Curtis, 1994). The curve shape of PCA plots is particularly sensitive to SD; deviations provided informative supporting evidence for our SD analysis. Figure S1 has heat maps and PCA figures for all chromosomes.

### Genetic map comparisons

Table 1 compares NIAB2015 to four other genetic maps, listed in Table 2. CM2014 contains 40 267 markers (5564 unique sites) and is 11 185 cM in length. However, looking only at markers segregating in our MAGIC population, there are 1.19× as many markers but 1.58× as many unique sites as CM2014; this difference is much more pronounced for the A (1.68×) and B (1.64×) genomes than for the D genome (1.15×). The difference is probably related to the presence of the ‘Synthetic’ part of the SynOp cross included in CM2014; SynOp and CM2014 both have a proportionally higher number of unique sites in the D genome than NIAB2015. Our total number of unique sites is much higher than the other three maps, as expected; the 9K maps have far fewer markers, and SynOp is based on a single biparental cross and far fewer progeny lines. NIAB2015 is 51% of the length of CM2014, despite having 81% of the unique sites. In fact, for the A and B genomes, our number of unique sites per cM (0.85) is higher than any of the other maps (range 0.44–0.60, Table 1). However, for the D genome, the unique sites/cM is roughly the same as all other maps except 9KCONS and 9KMAGIC.

**Table 2:** List of high‐density wheat genetic maps used for comparison to NIAB2015 MAGIC map

Map	Markers	Population	Reference
SynOp	Genotyping‐by‐sequencing	Synthetic W7894 × Opata M85	Poland *et al*. (2012)
9KMAGIC	9K SNP array	4‐founder MAGIC	Cavanagh *et al*. (2013)
9KCONS	9K SNP array	9KCONS+6 bi‐parentals (inc. SynOp)	Cavanagh *et al*. (2013)
CM2014	80K SNP array	8 bi‐parentals (inc. SynOp)	Wang *et al*. (2014)

**Figure 3:**
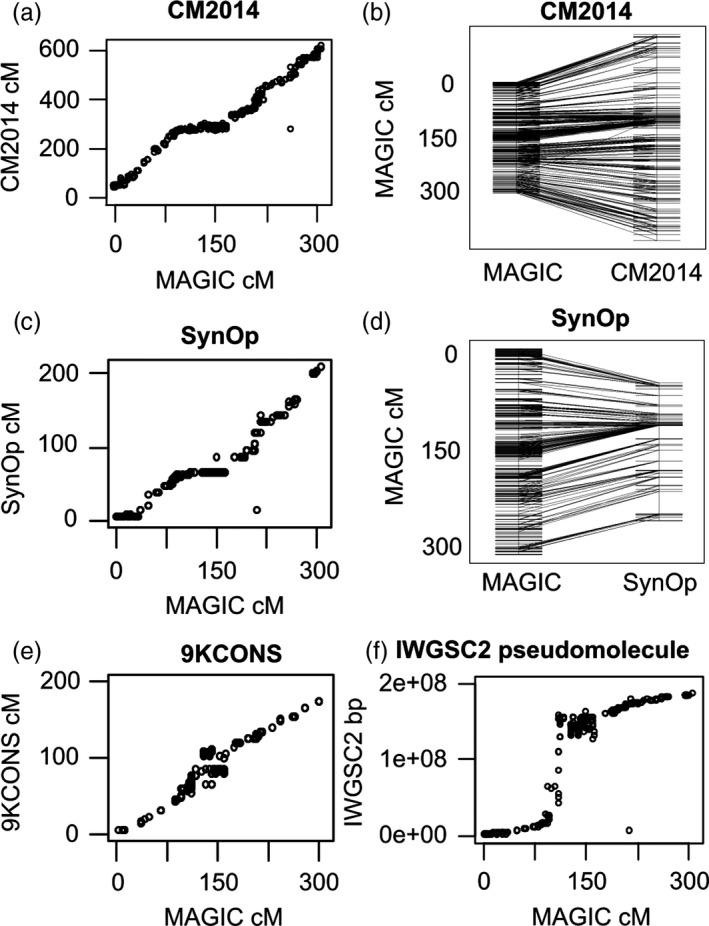
NIAB2015 chromosome 3A compared to four other genetic maps (a, b) CM2014 (c, d) SynOp (e) 9KCONS (f) IWGSC2 pseudomolecule. (a, c, e) cM‐cM genetic map comparison, NIAB2015 on *x*‐axis. (b, d) direct comparison between chromosome diagrams, NIAB2015 on left (f) comparison of NIAB2015 (cM,* x*‐axis) to IWGSC2 pseudomolecule (base pairs).

Figure 3 compares NIAB2015 chromosome 3A to the CM2014, 9KCONS and SynOp maps and also to the pseudomolecule POPSEQ data from IWGSC2, derived from SynOp. Other chromosomes are in Figure S2. There is a very low gradient in the middle of most chromosomes, that is NIAB2015 is much longer in this region than CM2014 and SynOp, as a result of more recombination events. This effect is either absent or weak (Figure S2) in the 9KCONS comparisons. There is still very low recombination directly at the centromere in NIAB2015 (Figure 3f), but the surrounding nonrecombining region is smaller than other non‐MAGIC maps. Figure 3b clearly demonstrates the centromeric effect in combination with the much more compact linkage map; at the distal ends of the chromosome, the markers fan outwards from NIAB2015 (left of the figure) to CM2014 (on the right) whereas in the centromeric region, the markers fan outwards from CM2014 to NIAB2015. Further examination of all chromosomes (Figure S2) reveals: (i) between NIAB2015 and CM2014, nonlocal map order in the D genome is less well conserved than in the A and B genomes; (ii) anomalous near vertical lines apparent on some chromosomes (e.g. 1B 100 cM, 4A 175 cM) reflect blocks of low recombination in the MAGIC population (possible introgression fragments); and (iii) two instances of missing chromosomal fragments. On chromosome 5A, the first 85 cM of NIAB2015 is largely absent in SynOp, whereas the first 15 cM of chromosome 5B in SynOp is largely missing in NIAB2015. The latter is the unmapped region around the translocation breakpoint of 5BS‐7BS.

**Figure 4:**
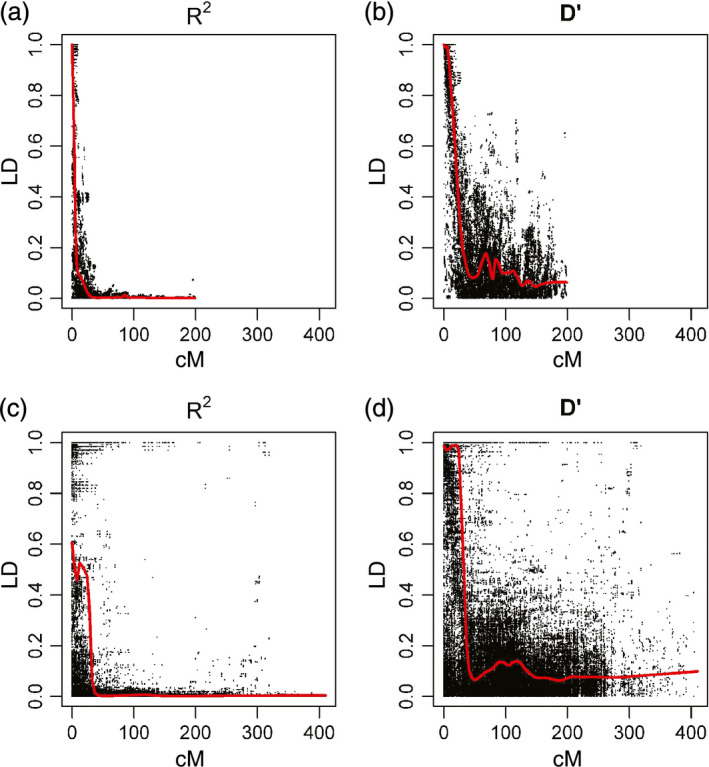
Linkage disequilibrium decay for chromosome 2D. (a, b) NIAB2015 (c, d) CM2014. Red line is best fit lowess curve with smoothing span parameter = 0.10.

Figure 4 compares LD decay for chromosome 2D in NIAB2015 with 2D in CM2014, as measured by *D*’ and *R* ^2^. For other chromosomes, see Figure S3. Graphically, it is apparent that the pattern of LD decay in the MAGIC map is considerably cleaner than in CM2014, with many more long‐distance high LD values apparent in CM2014.

### Alignment to 3B reference sequence

**Figure 5:**
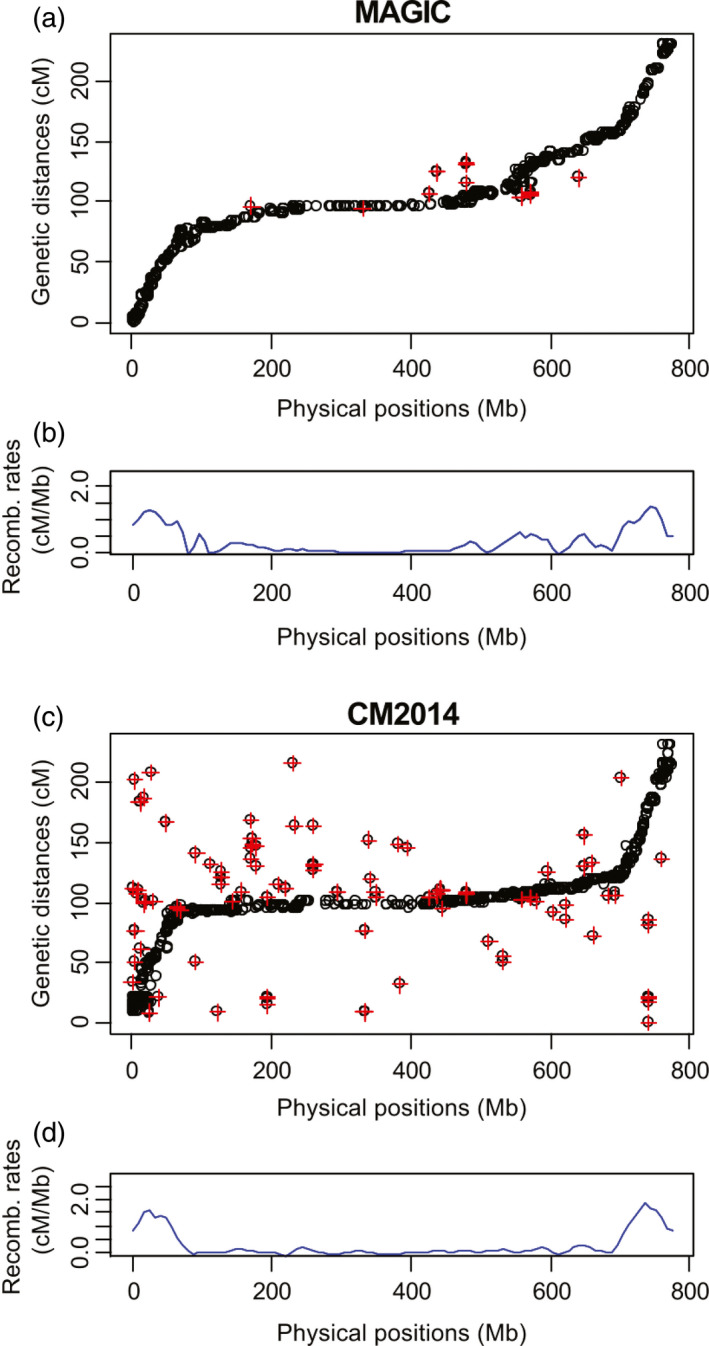
Chromosome 3B physical map comparison. (a, c) physical vs map distance for NIAB2015, CM2014. Note CM2014 scaled to be same length as NIAB2015. (b, d) recombination profile for NIAB2015, CM2014.

Comparison of the alignment of CM2014 and NIAB2015 to the physical map shows differences in quality of marker alignment as well as recombination landscape (Figure 5). 2.9% of the markers in NIAB2015 which align to the 3B pseudomolecule had inconsistent orders with respect to the reference sequence (identified by a red cross in Figure 5), while in CM2014 this is 10.46%. Nineteen of the 23 misaligned markers from NIAB2015 are also found in CM2014, which showed identical inconsistencies. This suggests the alignment problems for these markers may lie with their physical positions within the reference sequence, rather than the genetic maps. Consistent with this explanation, nine of the 23 anomalous markers also mapped to duplicated genes with different physical positions on 3B.

Detected crossovers on chromosome 3B vary by method of analysis: haplotype analysis in R/mpMap using R/happy.hbrem detects 2655 crossovers, equalling 1.46 crossovers per line per Morgan, while haplotype analysis using R/qtl detects 2920 crossovers or 1.60 per line per Morgan. The countXO function in R/qtl detects 4117 crossovers, equalling 2.26 per line per Morgan. Choulet *et al*. (2014) report 787 crossovers in the biparental Chinese Spring × Renan population. The distribution of crossovers in NIAB2015 varies significantly over the chromosome with average chromosome‐wide recombination rates of 0.30 cM/Mb and a maximum of 1.34 cM/Mb. The recombination landscape between NIAB2015 and CM2014 differs with proportionally higher recombination rates in the two distal chromosome regions (identified as 0–68 Mb and 715–774 Mb) in CM2014 (1.18 cM/Mb and 1.47 cM/Mb) compared to NIAB2015 (1 cM/Mb and 1 cM/Mb), but lower recombination rates in the large proximal regions (0.07 cM/Mb compared to 0.15 cM/Mb). Choulet *et al*. (2014) report distal recombination rates to be 0.60 and 0.96 cM/Mb and the large proximal region 0.05 cM/Mb. We observe falls in recombination rates around SD loci. SD loci associated with the founder Soissons are at 107–127 Mb and 242–414 Mb, while Robigus SD loci are at 145–150 Mb, 538–554 Mb and 641–663 Mb.

### Further genome sequence comparisons

For chromosomes where no physical map is currently available, we compared NIAB2015 map locations of markers which had top BLAST hits (>99% sequence identity) to the same CHAPMAP genomic sequence contig (Table 3, Appendix S1). Sixty‐six percent of markers could be assigned to a contig in CHAPMAP, but only 39% were in shared contigs. Twenty percent (22% for CM2014) of markers in singleton contigs were mapped to a different chromosome than the BLASTn hit but for markers in shared contigs, this number was only 5% (7% for CM2014). However, 76% of singleton and 81% of shared contig disagreements mapped to homeologous chromosomes. Combined with the high percentage of no hits, this strongly suggests that the incomplete sequence coverage of the genome (estimated 10.1/17 Gb, Chapman *et al*., 2015) explains most of the NIAB2015‐BLASTn discrepancies. Of markers in shared contigs on the same chromosome, 82% were fully consistent with our map order (same or adjacent location), a further 6% had a single nonshared unique site (usually a single marker) between the shared contig markers and the remaining 12% were further apart (median distance 6.6 cM) although 53% of these occurrences corresponded to separation by only 2–5 unique sites.

**Table 3:** Results of BLASTn analysis against Chapman *et al*. (2015)

	NIAB2015	CM2014^a^
Grouping		
No hit	34%	
Singleton	27%	
Same chr	80%	78%
Diff chr	20%	22%
Shared	39%	
Same chr	95%	93%
Diff chr	5%	7%
Ordering		
Same/adjacent map position	82%	
1‐site gap	6%	
Multisite gap	12%	
Multisite gap median cM dist	6.6 cM	
Multisite gap ≤5 sites	53%	

a CM2014 comparison used only markers also mapped in NIAB2015.

### Genome diversity analysis

**Figure 6:**
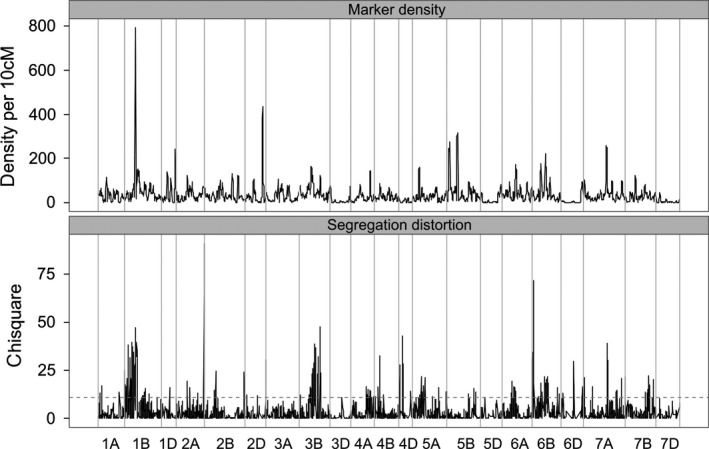
Genomewide patterns of marker density and segregation distortion in NIAB2015. Markers above the dotted line in the lower figure show significant SD at fdr < 0.01.

There has been no intentional trait selection within the MAGIC population except for the removal of double dwarf lines (lines carrying dwarfing alleles at both the *RhtB* and *RhtD* height loci). Mapping SD in the MAGIC population is thus a powerful approach to locate genomic regions causing meiotic segregation problems or under fertility or viability selection. Figure 6 shows the distribution of marker density and SD across the genome. A substantial fraction of mapped markers show statistically significant SD (Table 4): 2887 (15.5%) at the fdr<0.05 level, 1764 (9.5%) at fdr<0.01. If unmapped markers are included, these figures are higher: 16.4% fdr<0.05, 10.3% fdr<0.01. Markers exhibiting SD are nonrandomly distributed: 52% of mapped markers showing SD at fdr<0.01 map to chromosome 1B, mostly showing SD against the rye introgression (i.e. alleles from founders carrying the introgression are at lower than expected frequencies), and a further 12% of SD markers are found on each of chromosomes 3B and 6B. Otherwise, only 4A (6%) shows statistically significantly more SD loci than expected across the whole data set at the fdr <0.01 level (Table 4).

**Table 4:** Summary of segregation distortion results by chromosome

Chrom	Marker numbers		Percentage	
	SD, fdr < 0.05	SD, fdr < 0.01	%SD, fdr < 0.05	%SD, fdr < 0.01
ALL	2887	1764		
1A	12	3	0.42	0.17
**1B**	**1033**	**912**	**35.78**	**51.70**
1D	3	2	0.10	0.11
2A	37	8	1.28	0.45
2B	27	8	0.94	0.45
2D	7	5	0.24	0.28
3A	23	0	0.80	0.00
**3B**	**392**	**207**	**13.58**	**11.73**
3D	1	1	0.03	0.06
**4A**	**138**	**114**	**4.78**	**6.46**
4B	60	50	2.08	2.83
4D	14	9	0.48	0.51
5A	162	84	5.61	4.76
5B	32	4	1.11	0.23
5D	5	1	0.17	0.06
6A	122	38	4.23	2.15
**6B**	**502**	**213**	**17.39**	**12.07**
6D	**82**	18	**2.84**	1.02
7A	159	51	5.51	2.89
7B	60	36	2.08	2.04
7D	16	0	0.55	0.00

SD, segregation distortion; fdr, false discovery rate.

Bold = significantly more SD markers than expected for given chromosome (*P* < 0.01).

Some blocks of SD for particular founders occur over long distances along a chromosome, often >20 cM. Frequently, two single founders individually show weak SD along separate chromosome blocks but when these blocks overlap and the founders co‐occur, a much stronger SD effect is seen (e.g. on 2B a weak SD against Claire block starting at 33 cM overlaps at 98.4–98.9 cM with a weak SD against Soissons block ending at 130 cM; the combined peak has stronger SD). Conversely, counteracting blocks (SD in favour of founder A but against founder B) cancel when overlapping. From these observations, we conclude that for many founder combinations, SD extends over considerable distances in the MAGIC population. We inferred 63 SD blocks at fdr < 0.01 (96 for fdr < 0.05), of which 39 consisted of at least two unique sites. This may be an underestimate: (i) it was difficult to conclude whether some widely separated SD blocks with identical founder patterns were one block or two; we treated them as one block if no contradictory founder pattern was found in between them (ii) as we can detect SD blocks only when they are adequately discriminated by the available markers, we will have by chance missed small SD blocks where the appropriate founder combination did not happen to occur (iii) a high frequency of strong SD markers are unmapped. Table 5 shows the 39 blocks with fdr < 0.01, covering more than one unique site. Blocks containing the dwarfing loci *RhtD* and *RhtB* rank 4th and 6th, with the direction of SD as expected (dwarfing allele *RhtB*‐*1b* is found only in Robigus and Soissons, *RhtD*‐*1b* is found in the other six founders but not in Robigus and Soissons).

**Table 5:** Major segregation distortion (SD) blocks detected in the NIAB2015 population, ordered by false discovery rate (fdr) value of peak marker

Rank	PEAK SD_fdr	No SNPs	No sites	Direction	Minority founder	Ch	Start (cM)	Finish (cM)	Range (cM)	HMD	Map distort	Known Locus
1	6.48E‐14	55	23	FOR	CL, SO, XI	6B	0.0	24.5	24.5		Yes	
**2**	**2.32E‐09**	**268**	**89**	**FOR**	**RO**	**3B**	**86.6**	**198.5**	**111.9**	**HMD**	**Yes**	
**3**	**2.67E‐09**	**931**	**80**	**AGAINST**	**BR, RI**	**1B**	**0.0**	**119.0**	**119.0**	**HMD**	**Yes**	**1BR**
4	1.69E‐08	8	2	FOR	RO, SO	4D	32.2	40.1	7.9			*RhtD*
5	7.35E‐08	15	6	FOR	AL, CL	7A	216.3	219.8	3.5	Edge	Yes	
6	2.78E‐07	16	10	AGAINST	RO, SO	4B	10.4	52.7	42.2			*RhtB*
7	7.85E‐07	17	5	AGAINST	RO, SO	7A	219.8	221.8	2.0			
8	2.63E‐06	4	2	AGAINST	SO	4D	3.1	4.1	1.0			
9	1.92E‐05	2	2	AGAINST	SO, XI	2B	372.4	376.0	3.5			
10	4.79E‐05	33	13	AGAINST	CL	7B	194.9	226.2	31.4			
11	5.37E‐05	88	27	FOR	XI	5A	58.1	118.6	60.5			
**12**	**5.43E‐05**	**351**	**56**	**FOR**	**BR, RO, SO**	**6B**	**81.7**	**149.6**	**67.8**	**HMD**	**Yes**	
13	7.96E‐05	13	7	FOR	SO, XI	7A	347.1	352.6	5.6	Edge		
14	1.71E‐04	13	9	FOR	RI, SO, XI	6A	84.6	113.0	28.4			
15	7.28E‐04	12	6	AGAINST	BR, SO	4A	145.5	158.7	13.2			
16	7.39E‐04	70	9	AGAINST	SO	6D	192.7	204.6	11.9			
17	7.86E‐04	47	17	FOR	XI	6B	227.7	252.3	24.6			
18	8.16E‐04	18	5	AGAINST	XI	5A	242.5	249.2	6.6			
19	8.46E‐04	4	3	FOR	RI, SO	2A	127.6	130.7	3.0			
20	1.06E‐03	11	6	FOR	HE, RO, SO	1B	183.6	210.6	27.0			
21	1.12E‐03	7	5	FOR	HE, SO, XI	5B	251.9	265.0	13.2			
22	1.45E‐03	16	8	FOR	AL	7B	207.8	216.4	8.6			
23	1.46E‐03	40	4	FOR	RI	4B	54.7	61.3	6.6			
**24**	**1.63E‐03**	**99**	**14**	**AGAINST**	**RO**	**4A**	**170.1**	**210.2**	**40.1**	**HMD**	**Yes**	
**24** a	**1.35E‐11**	**62**	**n/a**	**AGAINST**	**RO**	**4A**	**218.3**	**218.3**	**0.0**			
25	1.70E‐03	87	36	AGAINST	AL, CL, RO	7A	240.1	310.3	70.2			
26	1.77E‐03	5	3	AGAINST	CL, SO	2B	98.5	101.0	2.5			
27	2.06E‐03	2	2	FOR	HE, RI	4D	104.5	106.0	1.5			
28	2.06E‐03	22	5	AGAINST	RO, SO, XI	5A	310.0	313.6	3.5			
**29**	**2.21E‐03**	**96**	**12**	**FOR**	**HE, SO, XI**	**6A**	**125.9**	**137.0**	**11.1**	**HMD**		
30	3.12E‐03	7	4	AGAINST	XI	6D	11.1	19.3	8.1			
31	3.43E‐03	12	7	AGAINST	BR	2A	152.5	207.6	55.1			
**32**	**3.73E‐03**	**120**	**12**	**AGAINST**	**SO**	**3B**	**67.7**	**114.2**	**46.5**	**HMD**	**Yes**	
33	4.09E‐03	22	4	FOR	CL	5B	202.4	210.0	7.6			
34	5.41E‐03	18	4	FOR	XI	4A	150.1	154.7	4.6			
35	5.52E‐03	55	19	AGAINST	RI, XI	1B	142.2	205.0	62.8			
36	5.81E‐03	3	2	FOR	RO	6D	215.0	215.5	0.5			
37	6.00E‐03	2	2	FOR	RI	2D	121.0	122.6	1.5			
38	8.26E‐03	11	10	AGAINST	HE	5A	181.1	215.0	33.9			
39	8.34E‐03	48	9	FOR	SO	6B	47.6	79.2	31.6	Edge		

HMD‐SD (‘High marker density segregation distortion’) blocks in bold. Minority founder = origin of minority allele: AL Alchemy, BR Brompton, CL Claire, HE Hereward, RI Rialto, RO Robigus, SO Soissons, Xi Xi19. Ch = chromosome, HMD = overlaps with high‐density block (HMD = in high‐density block, edge = border of HMD‐block). Map distort = visual evidence of map distortion.

a Additional unmapped markers almost certainly belong here (see text).

Marker density peaks around the centromere in many chromosomes but noncentromeric high marker density (HMD) blocks are also visible on 1D, 2D, 3B, 4A, 5A, 5B, 6B, 7A and most notably on 1B in the centre of the region containing the rye introgression (SNP density up to 793 markers/10 cM). Twenty‐seven noncontiguous blocks of markers contain more than 100 markers/10 cM. Of these, 11 are centromeric without significant SD and three are both centromeric and overlap SD blocks. Six SD blocks are associated with noncentromeric HMD blocks (Table 5, bolded). These include the blocks with the 2nd and 3rd highest chi‐squared peaks: a block with SD favouring Robigus alleles on 3B and the 1B‐1R rye introgression (SD against Brompton and Rialto) on 1B. Block 24, against Robigus on 4A, is under‐represented in this table: 74 unmapped markers showing significant SD against Robigus were mapped as traits to this location, with lower SD *P*‐values, which would make this the 2nd strongest block. Furthermore, these six SD‐HMD blocks are very long: they are the six blocks with the highest number of SD markers in Table 3, 5 of the top 6 with most unique sites (all 6 within top 12) and the two longest cM ranges (5 of top 10). These large SD regions posed considerable mapping challenges (chromosomes 1B, 3B and 6B, Figure S1), caused PCA plot distortions (1B, 3B, 4A, Figure S1), or were clearly visible in map comparisons (1B, 4A, Figure S2).

Robigus is thought to have emmer wheat (*Triticum dicoccoides*) in its pedigree (P. Werner, pers. comm.), but the location of any introgressions has not previously been reported. We examined the occurrence of the Robigus alleles in the two SD‐HMD Robigus blocks using the Bristol University 820K SNP array database (http://www.cerealsdb.uk.net, Wilkinson *et al*., 2012). In the 820K data set, 7202 SNPs were found in Robigus and its descendants but in no more than 15% of lines with known non‐Robigus pedigree, and could be assigned an IWGSC2 location with high certainty using BLASTn (Appendix S1). Of these, 481 mapped to the MAGIC 3B SD‐HMD block, most densely in the distal part of the block containing the peak SD markers. Almost all SNPs in this region had 4–6 non‐Robigus pedigree lines carrying Robigus alleles: Oratorio, Moisson, Garcia and Bacanora consistently carried Robigus alleles, while Dekan and Highbury did so sporadically. In addition, Robigus alleles for most markers in the peak region are found in the variety Glasgow, also suspected to have *T. dicoccoides* in its pedigree (R. Jennaway, pers. comm.). Within the core 3B SD block, 10 ‘perfect match’ Robigus SNPs (i.e. found in 0 non‐Robigus pedigree lines) are found; however, they are not the peak markers and all have ‘no call’ as the Robigus allele. In contrast, 155 ‘perfect match’ Robigus markers mapped exactly to the MAGIC 4A SD‐HMD block (175–218 cM in MAGIC map), none of which had null alleles. This represents 23% of all perfect, null‐free Robigus markers which could be assigned an IWGSC location in the entire 820K data set. Furthermore, when we grouped all the perfect, null‐free Robigus markers into blocks (<0.75 Mb separation between markers in IWGSC2 pseudomolecule) along chromosomes, this block was the largest, containing nearly three times as many markers as the 2nd largest block.

## Discussion

### Linkage map

We have constructed the first eight‐parent MAGIC genetic map for wheat, comprising 18 601 mapped markers. This required strict quality control of marker calling and extensive manual curation. During map construction, we dropped 10% of markers which could not be cleanly placed along a chromosome or which could not be placed without a large increase in map distance; these were biased towards dominant, null and SD loci and included a block of loci from around the translocation breakpoint of chromosomes 5BS and 7BS. To the best of our knowledge, this small block is the only specific missing chromosomal block in our map, and 5BS‐7BS was the only large‐scale chromosomal rearrangement we were able to detect.

There are two major practical benefits from constructing a genetic map in an eight‐founder MAGIC population. Firstly, we obtain an increase in precision from a larger number of accumulated recombination events via three rounds of intercrossing. Secondly, while all high‐density genetic maps are by necessity an average across several parental combinations, a MAGIC map produced in a single mapping experiment should have greater accuracy (lower error rate) than a map produced by merging data from several separate biparental crossing populations. Comparing our eight‐founder MAGIC genetic map (‘NIAB2015’) to published high‐density wheat genetic maps, including the current reference standard CM2014 (Wang *et al*., 2014), we can see that NIAB2015 has either considerably more total unique sites or, compared to CM2014, more unique sites for mutually shared markers (Table 1, Figures 3 and S2). Confirming the advantage of increased recombination in multiparental populations, we have a considerably higher number of unique sites per cM in the A and B genomes than all other maps; NIAB2015 is much more compact. The most visible evidence of increased recombination in NIAB2015 is in the pericentromeric regions (Figures 3, 5 and S2) where NIAB2015 has many more unique sites than other maps. Of all map comparisons our map has most in common with the four‐parent wheat MAGIC map (Cavanagh *et al*., 2013), which has a close to linear relationship with NIAB2015 for most chromosomes, (i.e. an overall similar recombination pattern along the chromosome), confirming the expectation that MAGIC maps exhibit greater precision.

We quantified recombination rates in NIAB2015 using chromosome 3B, for which a physical map is available. Broman (2005) calculates for an eight‐founder MAGIC population the expected number of informative crossovers per Morgan per line to be on average 4. Our estimates were 1.46 using R/happy.hbrem and 1.60 using R/qtl in mpMap with the mpprob function, and 2.26 using the countXO function in R/qtl. Similar to our countXO estimate, Huang *et al*. (2012) also observed about half the number of recombinations expected from simulations in a four‐parent MAGIC population. In the case of using inferred haplotypes from ‘mpprob’ to calculate crossovers, this underestimation can partially be attributed to the extent of missing haplotype data (18.61% using R/qtl, 37.46% using R/happy.hbrem). The other main reason for underestimation of crossovers is that each SNP is not fully informative in a multiparent population, making it not possible to count all recombination events directly. Nevertheless, using R/qtl (Broman *et al*., 2003), we detected over 5× as many crossovers on Chromosome 3B (using the function countXO) as in the Chinese Spring × Renan population of Choulet *et al*. (2014).

A comparison between recombination rates on chromosome 3B using NIAB2015 and CM2014 shows that NIAB2015 has a much cleaner estimation of recombination rate as recombination rates rarely drop below 0 cM/Mb (Figure 5). NIAB2015 has a slightly higher rate of recombination in the central chromosomal region compared to CM2014, and an 18–42% lower estimated recombination rate in the distal regions. A possible explanation is that our map distances are inflated in the proximal regions, due to the increased number of unique proximal recombination fraction bins compared to CM2014. Genetic mapping errors that will lead to over‐ or underestimation of recombination rates, especially in the pericentromeric regions, could also be a confounding problem. Furthermore, the two SD blocks on 3B contributed to lower recombination estimates, a potential problem for all estimates of recombination rates from multifounder populations. Interestingly, Maccaferri *et al*. (2015) also report that CM2014 shows a much lower recombination rate throughout the centromeric–pericentromeric regions compared to tetraploid wheat.

Theoretically, an increase in unique sites in our map compared to others may result from erroneous ordering falsely breaking up linkage blocks, or a higher genotyping error rate, rather than from increased recombination. Several lines of evidence suggest this is not the case. For genotype calling, we explicitly aimed for high accuracy by a combination of strict QC and manual calling, and estimated error rates at several stages of the process are very low (Appendix S1). Furthermore, very few recombination bins contain only 1 marker. More generally, our detailed physical map comparison to chromosome 3B (Figure 5) shows a considerably lower rate of disagreement (2.9%) than in CM2014 (10.3%). Our marker order on 3B, especially in the pericentromeric region, matches the physical map, which would not be expected if many unique sites resulted from errors. Local inversions would also show up as negative recombination values in Figure 5 (as they do in CM2014). For other chromosomes, assessing absolute accuracy of our map, especially of grouping, is hampered by the incompleteness of the wheat genome sequences. Comparatively, grouping was better than in CM2014. For ordering, our results are in strong agreement with the 3B results. Although 18% of markers in shared contigs were not in identical or adjacent map positions, it is highly unlikely that both markers are erroneously mapped, so the best estimate of our *error* rate is 9%, of which 6% is very local (1–5 unique sites). The remaining 3% of longer‐range error matches the 2.9% visible component seen in Figure 5 for chromosome 3B. For 3B, our evidence suggests that some of this error results from duplicate genes. In summary, we have some minor local ordering issues common to most high‐density genetic maps, but have greatly improved longer‐range error compared to CM2014. A caveat of using MAGIC populations for mapping is that map accuracy may be diminished in SD regions. Shah *et al*. (2014) developed an approach to minimize this effect for distortions involving a single founder SD locus, which we used successfully on chromosome 3B. Until a more general methodology is developed, we believe that including and noting the introgressions, as we do here, is the optimal approach. Overall, we are confident that our map represents a significant improvement in both precision and map accuracy over previously published maps. The completed map is available for download from http://www.niab.com/pages/id/326/Resources.

### Genome diversity

Given the lack of deliberate selection on all but one trait, plant height, we found a remarkably extensive and diverse array of linkage blocks showing SD in our MAGIC population (Figure 6, Table 5). Many of these extend for substantial distances along the chromosome (16 blocks > 20 cM), suggesting that the underlying cause was operating from the earliest intercrossing generations. This unanticipated degree of SD may reflect negative interactions between linkage blocks from different genetic backgrounds in a multifounder population, or simply reflect lower detection ability in previous biparental and low‐marker‐density populations. We assume that most of these blocks either cause meiotic problems or are subject to some form of viability or fertility selection. There is evidence in wheat for meiotic problems causing SD, particularly in interspecific crosses. For example, gametocidal genes from *Aegilops* species are expressed in crosses to bread wheat (Endo, 1990) and in general crosses between even closely related species such as durum wheat and *T. dicoccoides* show considerable SD (Avni *et al*., 2014).

Several interspecific introgression fragments have been incorporated into bread wheat germplasm in an attempt to broaden the genetic base and improve specific traits such as disease resistance and yield (e.g. 1B rye introgression). Often such fragments are known to exhibit SD. Such cases are likely to be characterized by a high marker density as well as significant SD for two reasons: (i) a higher frequency of markers will be polymorphic between species than within species (ii) reduced recombination between the introgressed fragment and native chromosomes. Six major SD fragments were also associated with HMD blocks in our data set, including 3 of the 4 strongest and 5 of the 10 longest SD blocks. The top 3 are the well‐known rye introgression on 1B (Worland and Snape, 2001), with strong SD against the introgression, and two blocks with Robigus alone as the minority founder. Robigus is thought to have *T. dicoccoides* in its pedigree, but the locations of introgressed fragments in Robigus and its descendent lines have been a matter of speculation. One of the Robigus SD‐HMD blocks at the distal end of chromosome 4AL (170–210 cM) is a perfect fit to the model of an interspecific introgressed fragment, with strong SD against Robigus in MAGIC centred on an HMD block, and restriction to Robigus and Robigus descendants only in the 820K SNP array data set from Bristol University. In the 820K data set, this block is by far the largest linkage block of Robigus‐restricted markers, including 23% of all perfect match Robigus SNPs on the array. The other SD‐HMD Robigus linkage block in MAGIC, on chromosome 3B, is being strongly selected *for* in the MAGIC population (allele frequency 1.8× expectation). The presence of a SD‐HMD block in MAGIC, the long chromosomal range of the effect and the occurrence of Robigus alleles in Glasgow all suggest the presence of a *T. dicoccoides* introgression fragment. On the other hand, this SD block is clearly found in some older UK wheat lines and some of their more recent descendants, suggesting it is either of hexaploid wheat origin or there has been at least one more, older introgression of the fragment into the UK wheat gene pool. In the 820K array, alleles of both these Robigus fragments occur sporadically in accessions of wheat wild relatives, including a single *T. dicoccoides* accession, so definitive assignment to *T. dicoccoides* could not be confirmed from this source. Forthcoming genomic resources for *T. dicoccoides* (F. Leigh, M. Caccamo, pers. comm.) will help resolve this question.

Knowledge of the location of interspecific introgressions and other linkage blocks containing useful alleles but showing negative interactions in common genetic backgrounds is important in disentangling their desirable and undesirable effects and in tracking their inheritance during breeding. Identifying the two Robigus SD blocks is an exemplar of the potential of the MAGIC approach to mapping to aid in this process. We are presently researching the underlying genetic causes and phenotypic effects of these complex SD‐HMD and translocation patterns in our MAGIC population in further detail.

## Methods

### Plant material

Details of the MAGIC population construction are given in Mackay *et al*. (2014) and Appendix S1. Genotyping was performed using the Illumina Infinium iSelect 80 000 SNP wheat array (‘80K array’, http://www.illumina.com/), described in Wang *et al*. (2014).

### SNP genotype calling

The polyploid module of Genome Studio V2011.1 (Illumina, San Diego, CA) developed for wheat by Wang *et al*. (2014) is poorly suited to genotype calling in our MAGIC population, due to the presence of a low‐density heterozygote cluster in most assays. We used a strategy of strict QC/error quantification followed by manual curation of all assays that failed to pass QC—about half the markers in the final data set. This data set is a larger, higher quality version of that presented in Mackay *et al*. (2014). See Appendix S1 for details.

### Map construction

The MAGIC map was constructed in two steps using the R package mpMap version 1.25 (Huang and George, 2011) available from github (https://github.com/behuang/mpMap). For the first round of mapping, we used a subset of 18 750 SNP markers scored as co‐dominant. All heterozygote calls were set to missing. These markers were then filtered for missing founder genotypes as well as SD with *P*‐values <1e‐5. Recombination fractions between all pairs of markers were calculated using the function ‘mpestrf’ at default values. Markers were grouped hierarchically using the ‘mpgroup’ function into 300 linkage groups. These linkage groups were merged using R/mpMapInteractive (https://github.com/rohan-shah/mpMapInteractive) to produce larger groups which could then be assigned chromosome names based on marker groupings in previous genetic maps (Cavanagh *et al*., 2013; Wang *et al*., 2014; Genomezipper v5 (http://wheat-urgi.versailles.inra.fr/Seq-Repository/Genes-annotations) and in the Kansas deletion lines genotyped for the 80K array by Bristol University (http://www.cerealsdb.uk.net), as well as top BLASTn (Altschul *et al*., 1990) hits to IWGSC contigs listed in Wang *et al*. (2014, Table S6).

Within linkage groups, markers were ordered using two‐point ordering implemented in the function ‘mporder’ using default settings. Fine ordering was performed interactively using R/mpMapInteractive. Map distances were computed using the Haldane mapping function using ‘computemap’. Once a draft map was built, previously excluded markers, including all those scored as dominant, were mapped as traits to the existing draft chromosomes, that is we treated the unmapped markers as phenotypes in a QTL analysis, similarly to Rostoks *et al*. (2006). Founder haplotype probabilities were computed with the ‘mpprob’ function in mpMap implemented in R/qtl (Broman *et al*., 2003) with a threshold of 0.6 and QTL were calculated using single‐stage QTL mapping in ‘mpIM’. Linkage groups were then reordered following the same steps as previously but including all the previously excluded loci which could be mapped to a chromosome with –log_10_ *P* > 16. In the construction of this final map, manual curation was used throughout the process and loci which could not be cleanly fitted into the ordered chromosomes were dropped.

### Map validation

Recombination fractions were visualized using R/mpMap, and by PCA of the recombination fraction matrix to qualitatively assess if chromosomes were well ordered. We plotted the decay of linkage disequilibrium (LD) against map distance in our MAGIC map, ‘NIAB2015’, and in the Wang *et al*. (2014) consensus map, ‘CM2014’, using R/popgen (Marchini, 2013) to calculate LD, then fitting a lowess curve with smoothing span parameter = 0.10. For cross‐chromosome comparisons to other published maps, we used a modified version of the ‘plotMap’ function in R/qtl (Broman *et al*., 2003). We validated our map against both published wheat genome sequences (Chapman *et al*., 2015; International Wheat Genome Sequencing Consortium, 2014). Flanking sequences of SNP markers in our map were initially blasted against both published genomes using BLASTn (Altschul *et al*., 1990) with an *e*‐value cut‐off of 1e‐20 and ≥99 identity. For SNP markers with multiple blast hits, we retained the top hit and all other hits which had an equal match or one mismatch worse than the top hit. Where the marker assay had only one segregating SNP and one of the multiple BLAST hits was the same as our map location, we selected this hit as our top hit. For IWGSC data, we converted IWGSC2 coordinates to IWGSC1 coordinates using EnsemblPlants (www.plants.ensembl.org).

### Alignment to 3B reference sequence

Flanking marker sequences for both NIAB2105 and CM2014 were aligned to the 3B pseudomolecule (Choulet *et al*., 2014; https://urgi.versailles.inra.fr/download/wheat/3B/) using BLASTn. We only included alignments which were part of the final 3B pseudomolecule and had no mismatches. The strong Robigus SD locus was controlled for using the method described in Shah *et al*. (2014), and map distances were re‐estimated using the Haldane mapping function. This 783‐marker long MAGIC chromosome 3B had a reduced length of 230 cM compared to the original of 284 cM with 1408 markers. The number of crossovers per line was calculated using the function ‘mpprob’ in mpMap with either the options program = ‘qtl” (Broman *et al*., 2003) or program = ‘happy’ (Mott *et al*., 2000) and a threshold of 0.5. In R/qtl the number of crossovers was calculated using the function ‘calculateXO’ on default settings for eight‐parent RILs. Recombination per physical distance (cM/Mb) was calculated and visualized using R/MareyMap 1.3.0 (Rezvoy *et al*., 2007). A cubic spline with a smoothing parameter of spar 0.65 was fitted to calculate local recombination rates. Markers with inconsistent alignments were excluded from the fitting. Chromosome 3B of CM2014 was aligned to the 3B pseudomolecule as described above (with a total of 1406 markers), and its genetic distance rescaled to match the reduced 3B MAGIC map length of 230 cM.

### Genome diversity analysis

Marker density was calculated in a 10‐cM sliding window along each chromosome to identify HMD blocks. Segregation distortion was estimated with chi‐squared tests using fdr correction for multiple comparisons. SD markers were assigned into linkage blocks by scanning along chromosomes for consistent patterns. We confirmed the SD assessment of all these blocks by manually rescoring up to 5 individual markers per SD block and recalculating SD. Flanking sequences of SNP markers in the 820K array data set were blasted against the IWGSC genome sequence in a similar manner to our map validation and then aligned to NIAB2015 via our IWGSC top hits. Details are given in Appendix S1.

## Conflict of interest

The authors have no conflict of interest to declare.

## Supporting information


**Figure S1** Heat maps of individual chromosomes in NIAB2015.

**Figure S2** Graphical comparison of all chromosomes to four genetic maps.

**Figure S3** LD decay (measured as *R* ^2^ and *D*') compared across all chromosomes for NIAB2015 and CM2014.

**Table S1** List of unmapped markers, with QTL positions, blast hits (see ‘Notes’ worksheet).

**Table S2** The NIAB2015 MAGIC map (see ‘Notes’ worksheet).

**Appendix S1** Methods (Genotype calling including genotype error calculation, mapping error rate estimation, SD analysis).

## References

[pbi12504-bib-0001] Altschul, S.F. , Gish, W. , Miller, W. , Myers, E.W. and Lipman, D.J. (1990) Basic local alignment search tool. J. Mol. Biol. 215, 403–410. 2231712 10.1016/S0022-2836(05)80360-2

[pbi12504-bib-0002] Avni, R. , Nave, M. , Eilam, T. , Sela, H. , Alekperov, C. , Peleg, Z. , Dvorak, J. *et al*. (2014) Ultra‐dense genetic map of durum wheat x wild emmer wheat developed using the 90K iSelect SNP genotyping assay. Mol. Breed. 34, 1549–1562.

[pbi12504-bib-0003] Badaeva, E.D. , Dedkova, O.S. , Gay, G. , Pukhalskyi, V.A. , Zelenin, A.V. , Bernard, S. and Bernard, M. (2007) Chromosomal rearrangements in wheat: their types and distribution. Genome, 50, 907–926. 18059554 10.1139/g07-072

[pbi12504-bib-0004] Bandillo, N. , Raghavan, C. , Muyco, P.A. , Sevilla, M.A.L. , Lobina, I.T. , Dilla‐Ermita, C.J. , Tung, C.W. *et al*. (2013) Multi‐parent advanced generation inter‐cross (MAGIC) populations in rice: progress and potential for genetics research and breeding. Rice, 6, 15. 24280183 10.1186/1939-8433-6-11 PMC4883706

[pbi12504-bib-0005] Broman, K.W. (2005) The genomes of recombinant inbred lines. Genetics, 169, 1133–1146. 15545647 10.1534/genetics.104.035212 PMC1449115

[pbi12504-bib-0006] Broman, K.W. , Wu, H. , Sen, S. and Churchill, G.A. (2003) R/qtl: QTL mapping in experimental crosses. Bioinformatics, 19, 889–890. 12724300 10.1093/bioinformatics/btg112

[pbi12504-bib-0007] Cavanagh, C. , Morell, M. , Mackay, I. and Powell, W. (2008) From mutations to MAGIC: resources for gene discovery, validation and delivery in crop plants. Curr. Opin. Plant Biol. 11, 215–221. 18295532 10.1016/j.pbi.2008.01.002

[pbi12504-bib-0008] Cavanagh, C.R. , Chao, S.M. , Wang, S.C. , Huang, B.E. , Stephen, S. , Kiani, S. , Forrest, K. *et al*. (2013) Genome‐wide comparative diversity uncovers multiple targets of selection for improvement in hexaploid wheat landraces and cultivars. Proc. Natl Acad. Sci. USA, 110, 8057–8062. 23630259 10.1073/pnas.1217133110 PMC3657823

[pbi12504-bib-0009] Chapman, J.A. , Mascher, M. , Buluc, A. , Barry, K. , Georganas, E. , Session, A. , Strnadova, V. *et al*. (2015) A whole‐genome shotgun approach for assembling and anchoring the hexaploid bread wheat genome. Genome Biol. 16, 26. 25637298 10.1186/s13059-015-0582-8 PMC4373400

[pbi12504-bib-0010] Cheema, J. and Dicks, J. (2009) Computational approaches and software tools for genetic linkage map estimation in plants. Brief. Bioinform. 10, 595–608. 19933208 10.1093/bib/bbp045

[pbi12504-bib-0011] Choulet, F. , Alberti, A. , Theil, S. , Glover, N. , Barbe, V. , Daron, J. , Pingault, L. *et al*. (2014) Structural and functional partitioning of bread wheat chromosome 3B. Science, 345, 7. 10.1126/science.1249721 25035497

[pbi12504-bib-0012] Curtis, D. (1994) Another procedure for the preliminary ordering of loci based on 2 point LOD scores. Ann. Hum. Genet. 58, 65–75. 8031015 10.1111/j.1469-1809.1994.tb00725.x

[pbi12504-bib-0013] Darvasi, A. and Soller, M. (1995) Advanced intercross lines, an experimental population for fine genetic‐mapping. Genetics, 141, 1199–1207. 8582624 10.1093/genetics/141.3.1199 PMC1206841

[pbi12504-bib-0014] Endo, T.R. (1990) Gametocidal chromsomes and their induction of chromosome mutations in wheat. Jpn. J. Genet. 65, 135–152.

[pbi12504-bib-0015] Huang, B.E. and George, A.W. (2011) R/mpMap: a computational platform for the genetic analysis of multiparent recombinant inbred lines. Bioinformatics, 27, 727–729. 21217121 10.1093/bioinformatics/btq719

[pbi12504-bib-0016] Huang, X.Q. , Paulo, M.J. , Boer, M. , Effgen, S. , Keizer, P. , Koornneef, M. and van Eeuwijk, F.A. (2011) Analysis of natural allelic variation in Arabidopsis using a multiparent recombinant inbred line population. Proc. Natl Acad. Sci. USA, 108, 4488–4493. 21368205 10.1073/pnas.1100465108 PMC3060268

[pbi12504-bib-0017] Huang, B.E. , George, A.W. , Forrest, K.L. , Kilian, A. , Hayden, M.J. , Morell, M.K. and Cavanagh, C.R. (2012) A multiparent advanced generation inter‐cross population for genetic analysis in wheat. Plant Biotechnol. J. 10, 826–839. 22594629 10.1111/j.1467-7652.2012.00702.x

[pbi12504-bib-0018] International Wheat Genome Sequencing Consortium . (2014) A chromosome‐based draft sequence of the hexaploid bread wheat (*Triticum aestivum*) genome. Science, 345, 11. 10.1126/science.1251788 25035500

[pbi12504-bib-0019] Maccaferri, M. , Ricci, A. , Salvi, S. , Milner, S.G. , Noli, E. , Martelli, P.L. , Casadio, R. *et al*. (2015) A high‐density, SNP‐based consensus map of tetraploid wheat as a bridge to integrate durum and bread wheat genomics and breeding. Plant Biotechnol. J. 13, 648–663. 25424506 10.1111/pbi.12288

[pbi12504-bib-0020] Mackay, I. and Powell, W. (2007) Methods for linkage disequilibrium mapping in crops. Trends Plant Sci. 12, 57–63. 17224302 10.1016/j.tplants.2006.12.001

[pbi12504-bib-0021] Mackay, I.J. , Bansept‐Basler, P. , Barber, T. , Bentley, A.R. , Cockram, J. , Gosman, N. , Greenland, A.J. *et al*. (2014) An eight‐parent multiparent advanced generation inter‐cross population for winter‐sown wheat: creation, properties, and validation. G3‐Genes Genomes Genet., 4, 1603–1610. 10.1534/g3.114.012963 PMC4169152 25237112

[pbi12504-bib-0022] Marchini, J.L. (2013) Popgen: Statistical and Population Genetics. R package version 1.0–3. https://cran.r-project.org/web/packages/popgen/popgen.pdf.

[pbi12504-bib-0023] Milner, G.M. , Maccaferri, M. , Huang, B.E. , Mantovani, P. , Massi, A. , Frascaroli, E. , Tuberosa, R. *et al*. (2015) A multiparental cross population for mapping QTL for agronomic traits in durum wheat (*Triticum turgidum* ssp. durum). Plant Biotechnol. J. 13, 648–663. 26132599 10.1111/pbi.12424 PMC11388855

[pbi12504-bib-0024] Mott, R. , Talbot, C.J. , Turri, M.G. , Collins, A.C. and Flint, J. (2000) A method for fine mapping quantitative trait loci in outbred animal stocks. Proc. Natl Acad. Sci. USA, 97, 12649–12654. 11050180 10.1073/pnas.230304397 PMC18818

[pbi12504-bib-0025] Pascual, L. , Desplat, N. , Huang, B.E. , Desgroux, A. , Bruguier, L. , Bouchet, J.P. , Le, Q.H. *et al*. (2015) Potential of a tomato MAGIC population to decipher the genetic control of quantitative traits and detect causal variants in the resequencing era. Plant Biotechnol. J. 13, 565–577. 25382275 10.1111/pbi.12282

[pbi12504-bib-0026] Poland, J.A. , Brown, P.J. , Sorrells, M.E. and Jannink, J.L. (2012) Development of high‐density genetic maps for barley and wheat using a novel two‐enzyme genotyping‐by‐sequencing approach. PLoS ONE, 7, 8. 10.1371/journal.pone.0032253 PMC3289635 22389690

[pbi12504-bib-0027] Rezvoy, C. , Charif, D. , Gueguen, L. and Marais, G.A.B. (2007) MareyMap: an R‐based tool with graphical interface for estimating recombination rates. Bioinformatics, 23, 2188–2189. 17586550 10.1093/bioinformatics/btm315

[pbi12504-bib-0028] Rostoks, N. , Ramsay, L. , Mackenzie, K. , Cardle, L. , Bhat, P.R. , Roose, M.L. , Svensson, J.T. *et al*. (2006) Recent history of artifical outcrossing facilitates whole‐genome association mapping in elite inbred crop varieties. Proc. Natl Acad. Sci. USA, 103, 18656–18661. 17085595 10.1073/pnas.0606133103 PMC1693718

[pbi12504-bib-0029] Sannemann, W. , Huang, B.E. , Mathew, B. and Léon, J. (2015) Multi‐parent advanced generation inter‐cross in barley: high‐resolution quantitative trait locus mapping for flowering time as a proof of concept. Mol. Breeding, 35, 86.

[pbi12504-bib-0030] Shah, R. , Cavanagh, C.R. and Huang, B.E. (2014) Computationally efficient map construction in the presence of segregation distortion. Theor. Appl. Genet. 127, 2585–2597. 25260690 10.1007/s00122-014-2401-0

[pbi12504-bib-0031] Thepot, S. , Restoux, G. , Goldringer, I. , Hospital, F. , Gouache, D. , Mackay, I. and Enjalbert, J. (2015) Efficiently tracking selection in a multiparental population: the case of earliness in wheat. Genetics, 199, 609–623. 25406468 10.1534/genetics.114.169995 PMC4317666

[pbi12504-bib-0032] Threadgill, D.W. and Churchill, G.A. (2012) Ten years of the collaborative cross. G3‐Genes Genomes Genet., 2, 153–156. 10.1534/g3.111.001891 PMC3284322 22384393

[pbi12504-bib-0033] Villareal, R.L. , Rajaram, S. , Mujeebkazi, A. and Deltoro, E. (1991) The effect of chromosome 1B/1R translocation on the yield potential of certain spring wheats (*Trtiticum aestivum*). Plant Breed. 106, 77–81.

[pbi12504-bib-0034] Wang, S. , Wong, D. , Forrest, K. , Allen, A. , Chao, S. , Huang, B.E. , Maccaferri, M. *et al*. (2014) Characterization of polyploid wheat genomic diversity using a high‐density 90,000 single nucleotide polymorphism array. Plant Biotechnol. J. 12, 787–796. 24646323 10.1111/pbi.12183 PMC4265271

[pbi12504-bib-0035] Wilkinson, P.A. , Winfield, M.O. , Barker, G.L.A. , Allen, A.M. , Burridge, A. , Coghill, J.A. , Burridge, A. *et al*. (2012) CerealsDB 2.0: an integrated resource for plant breeders and scientists. BMC Bioinformatics, 13, 219. 22943283 10.1186/1471-2105-13-219 PMC3447715

[pbi12504-bib-0036] Worland, A.J. and Snape, J.W. (2001) Genetic basis of worldwide wheat varietal improvement. In The World Wheat Book ( Bonjean, A.P. , Angus, W.J. , eds), pp. 59–100. Paris, France: Lavoisier.

[pbi12504-bib-0037] Yu, J.M. , Holland, J.B. , McMullen, M.D. and Buckler, E.S. (2008) Genetic design and statistical power of nested association mapping in maize. Genetics, 178, 539–551. 18202393 10.1534/genetics.107.074245 PMC2206100

