# Supplementary figures and images for: A highly recombined, high‐density, eight‐founder wheat MAGIC map reveals extensive segregation distortion and genomic locations of introgression segments
Source: Plant Biotechnol J. 2016 Jan 23;14(6):1406–17. doi: 10.1111/pbi.12504 (PMC4985697; doi:10.1111/pbi.12504)

# 1A

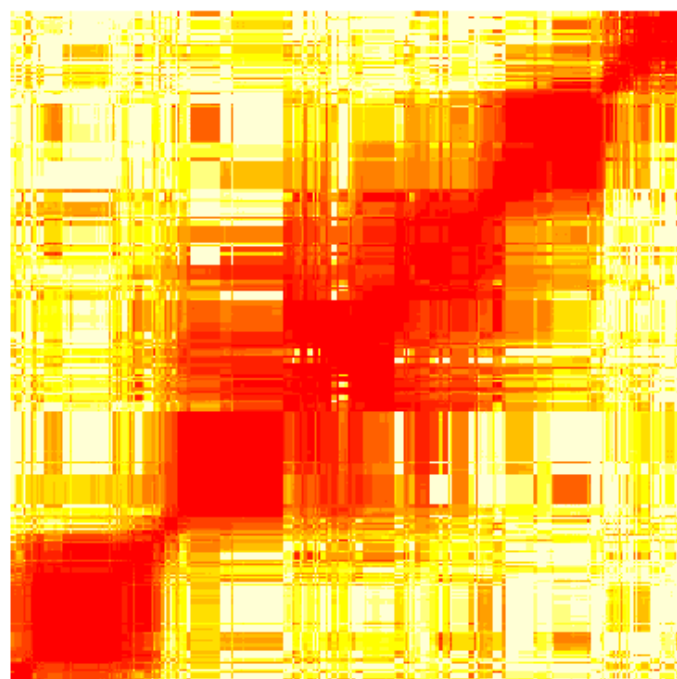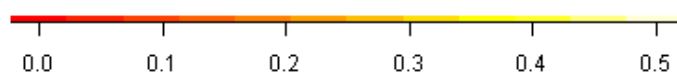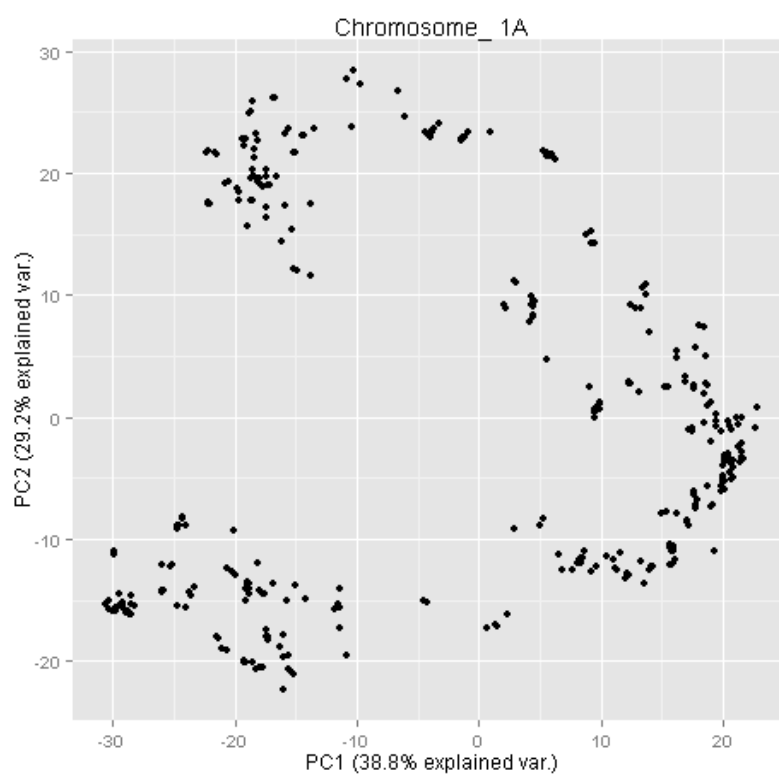

1B

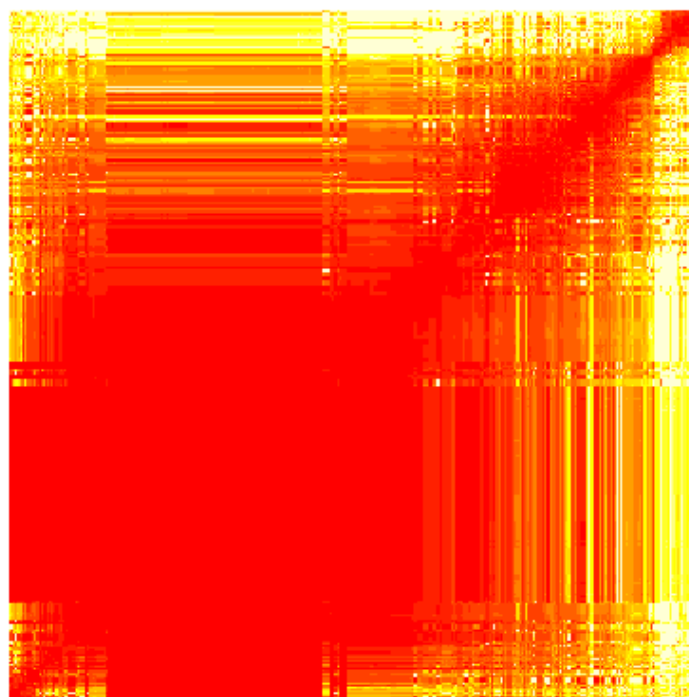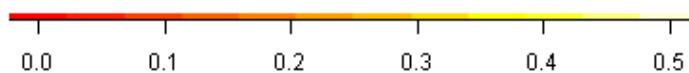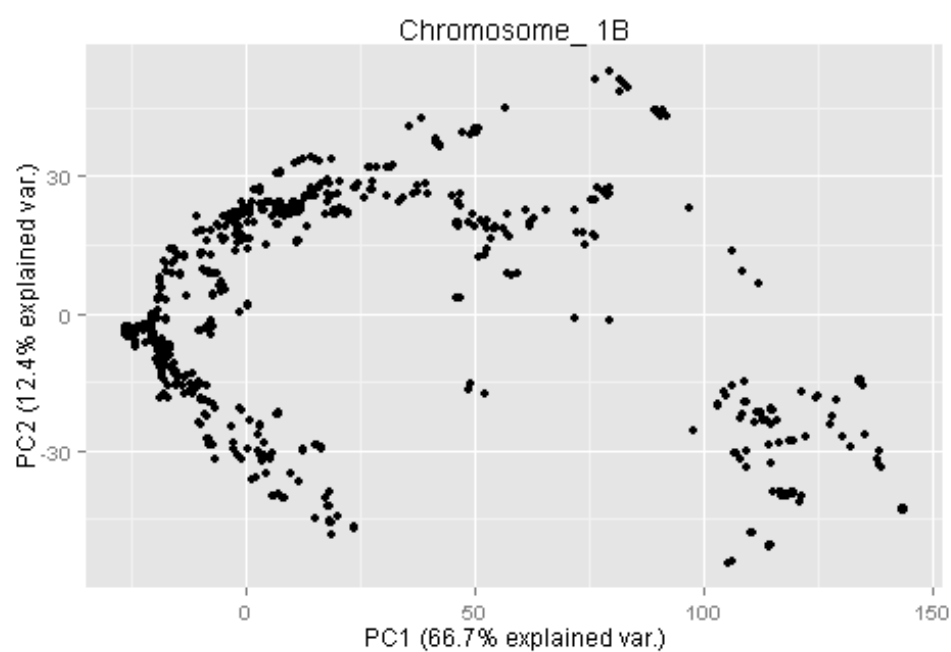

# 1D

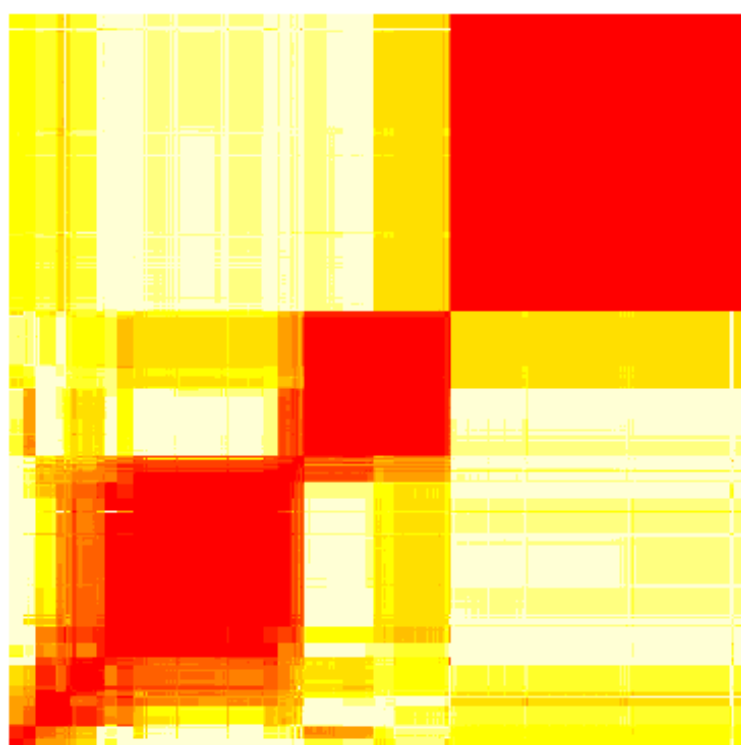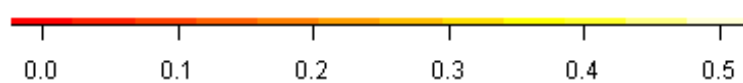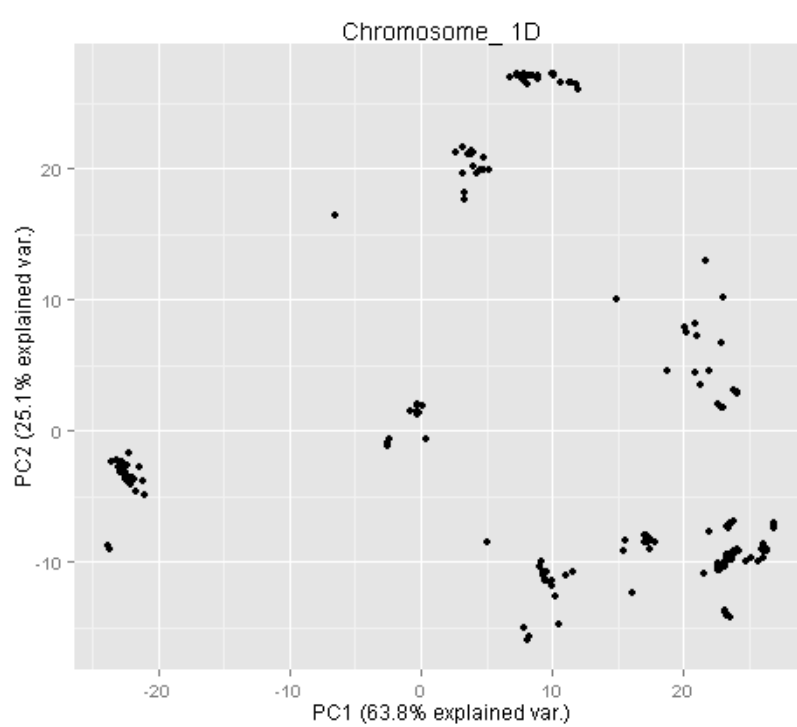

2A

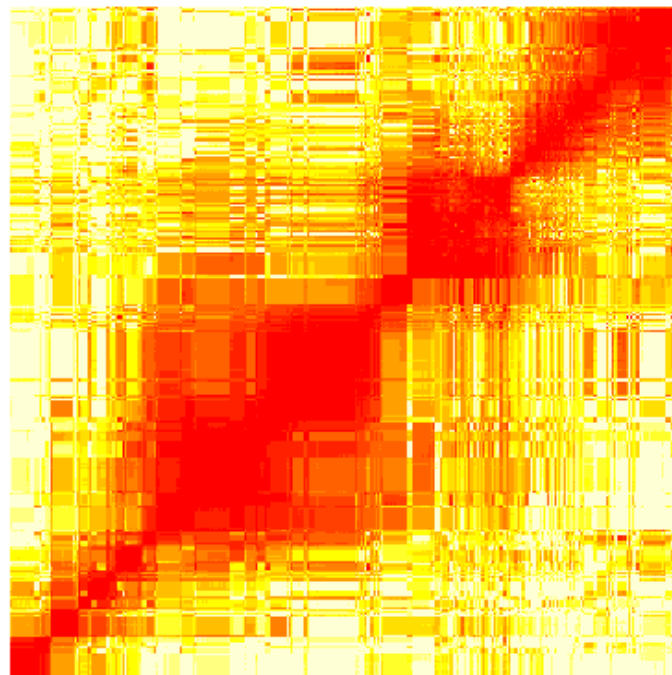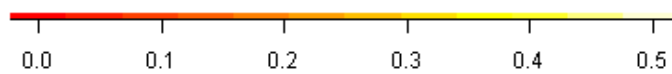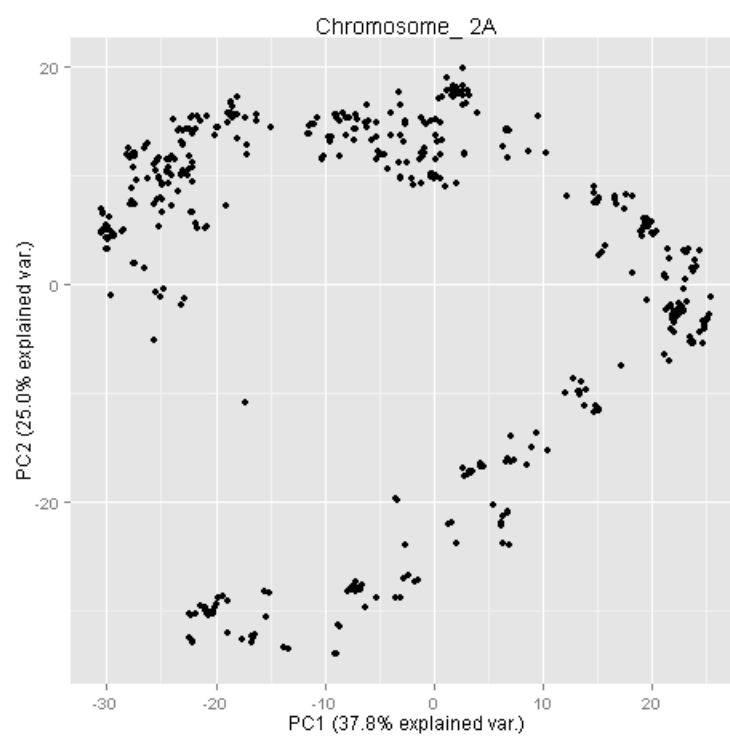

2B

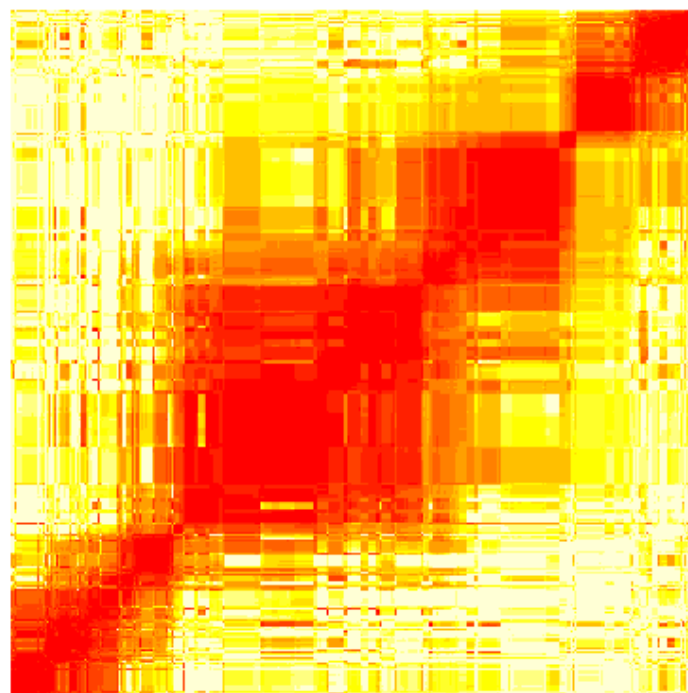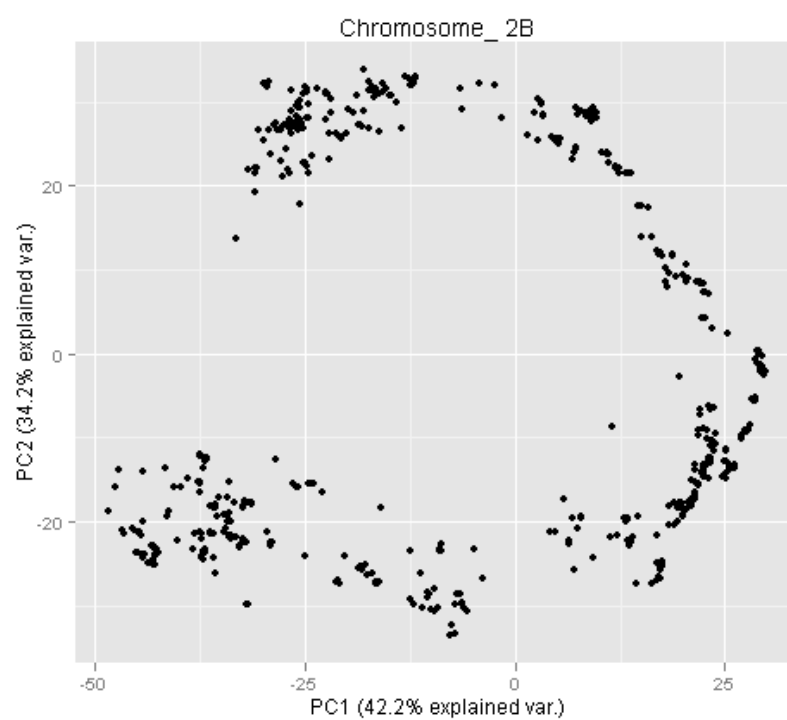

# 2D

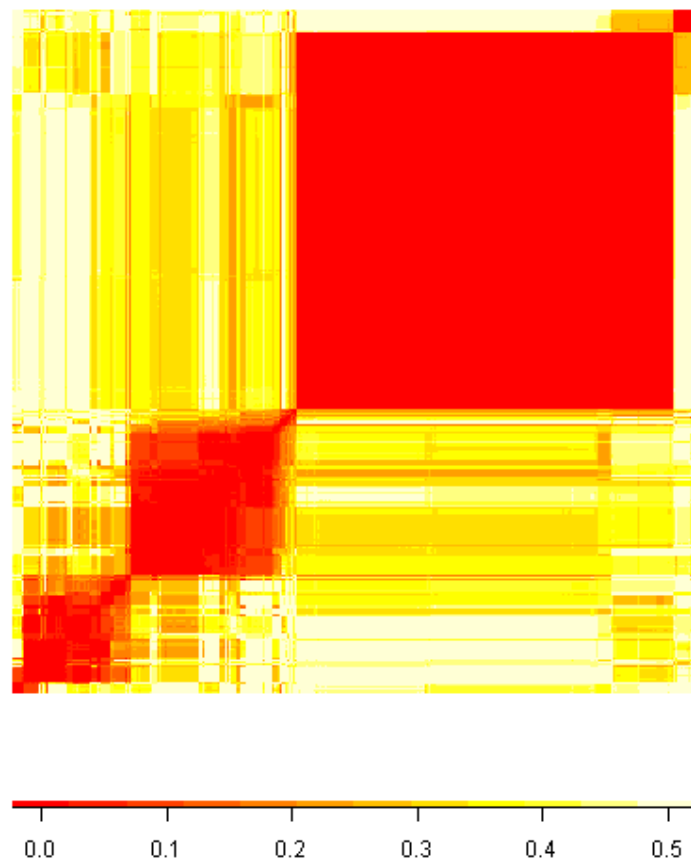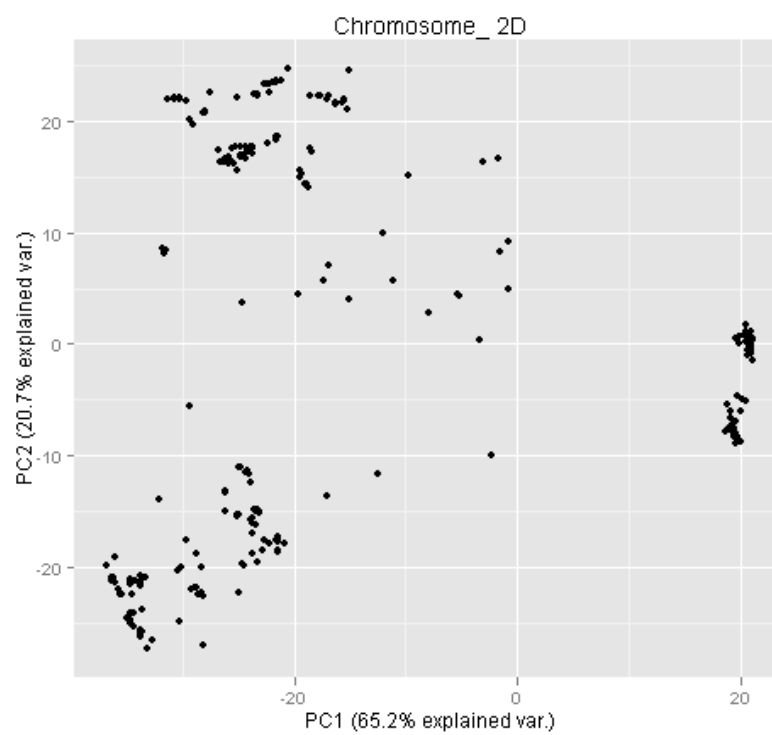

3A

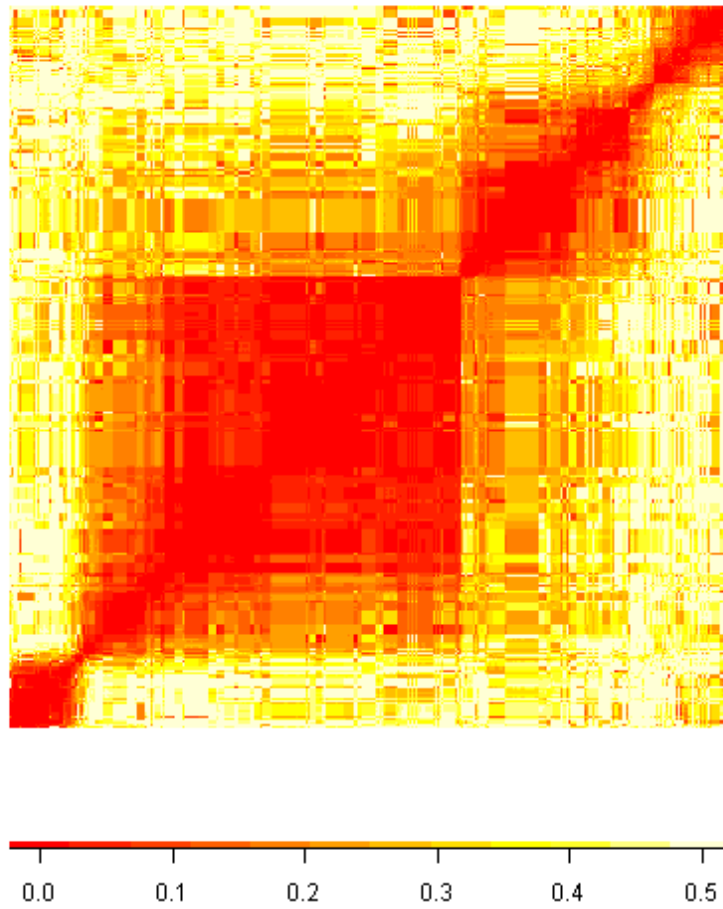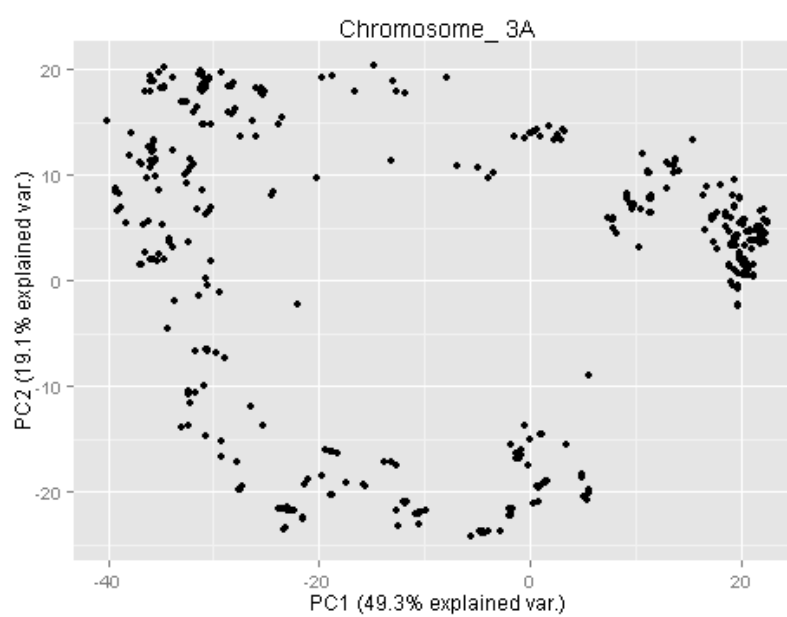

3B

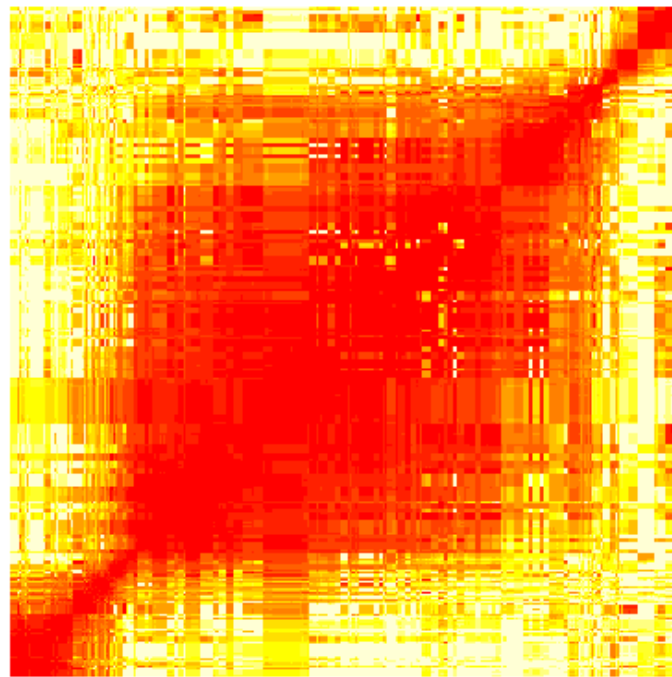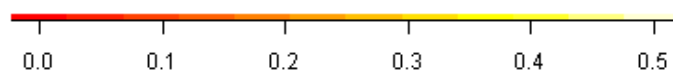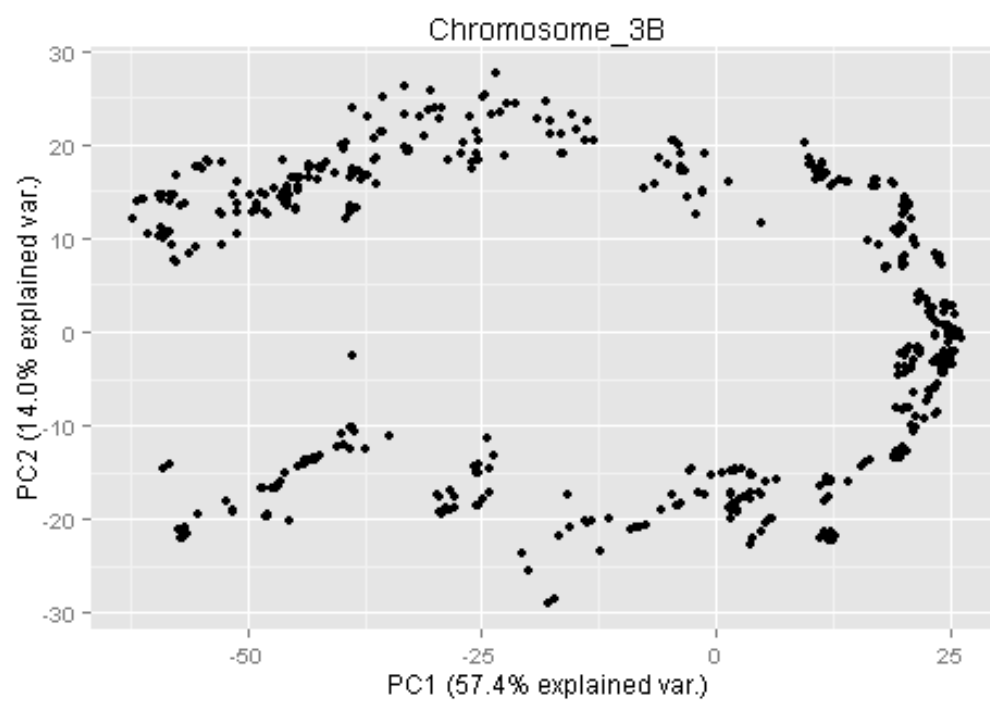

# 3D

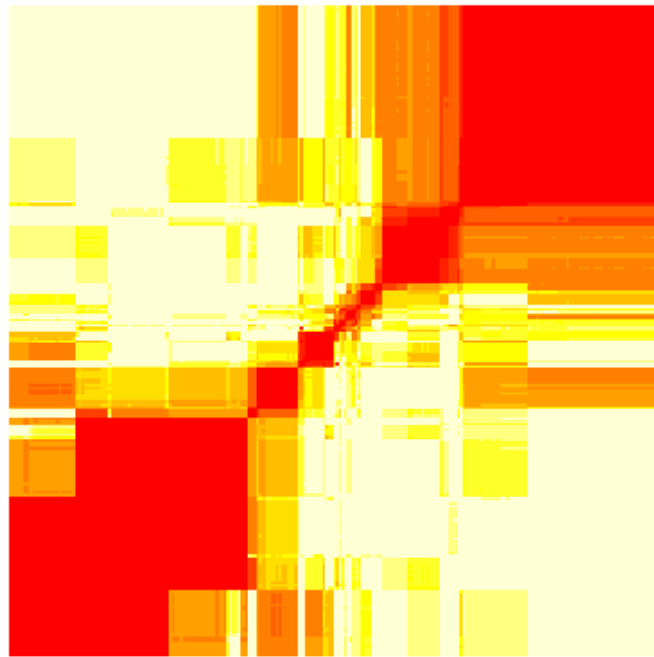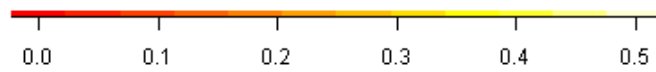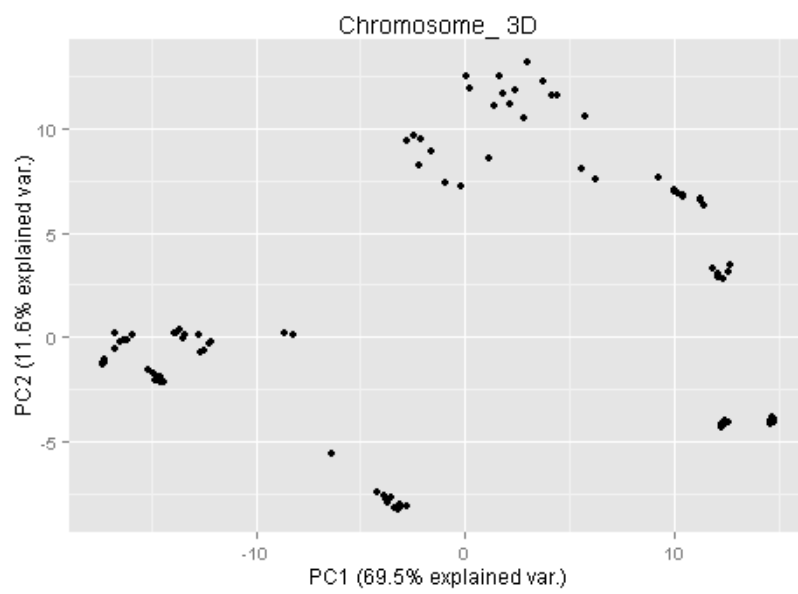

4A

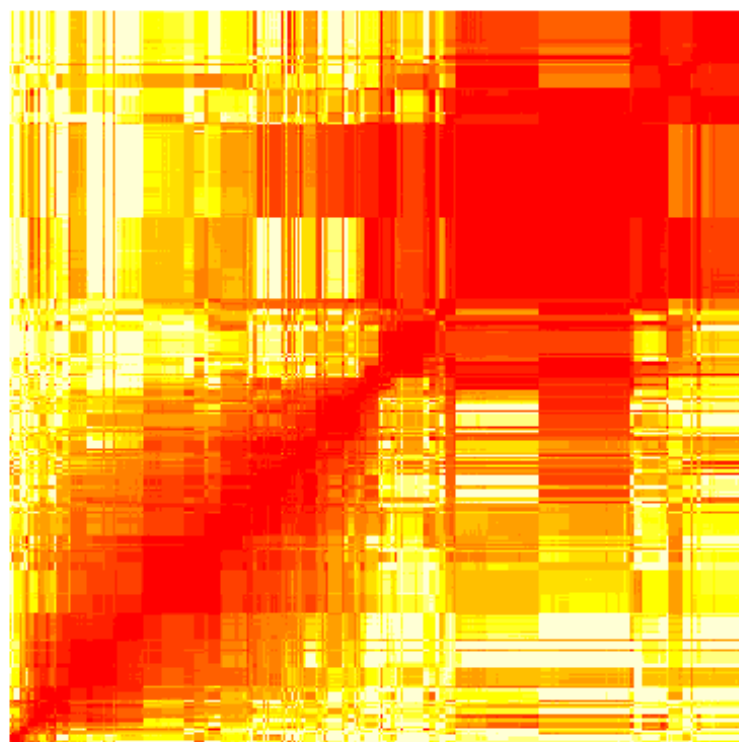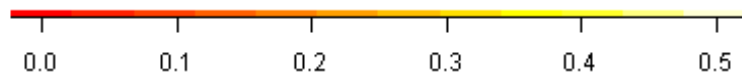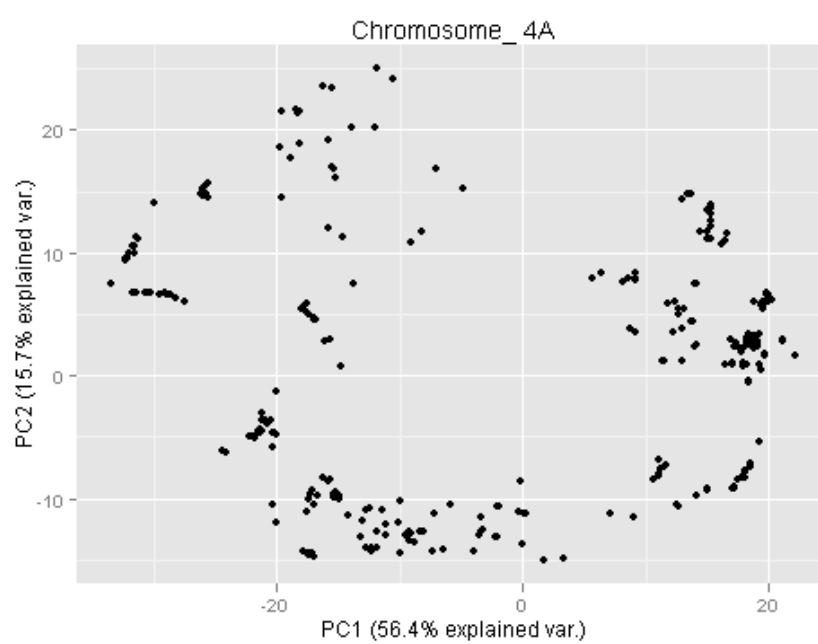

4B

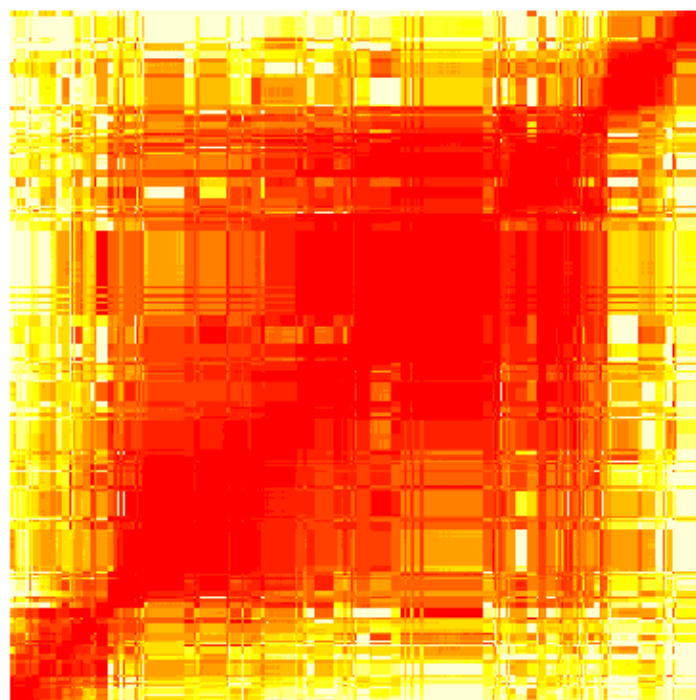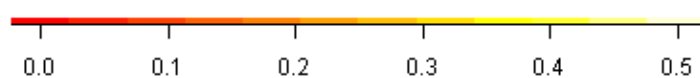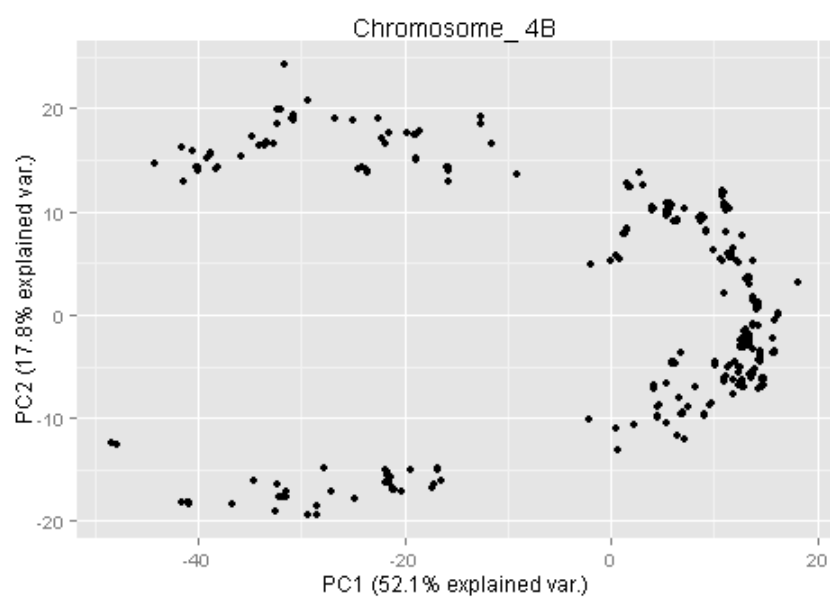

# 4D

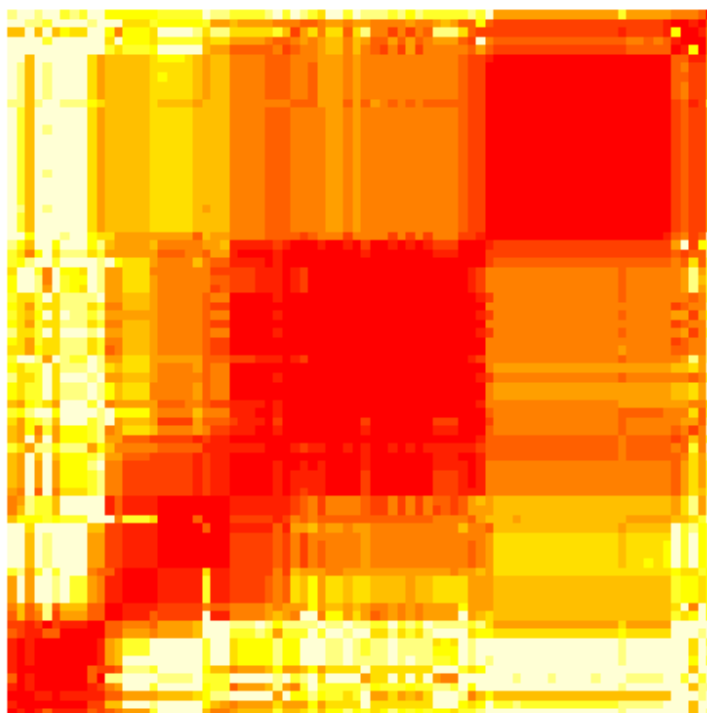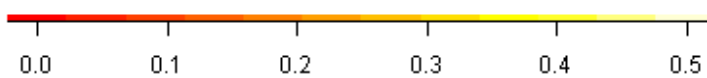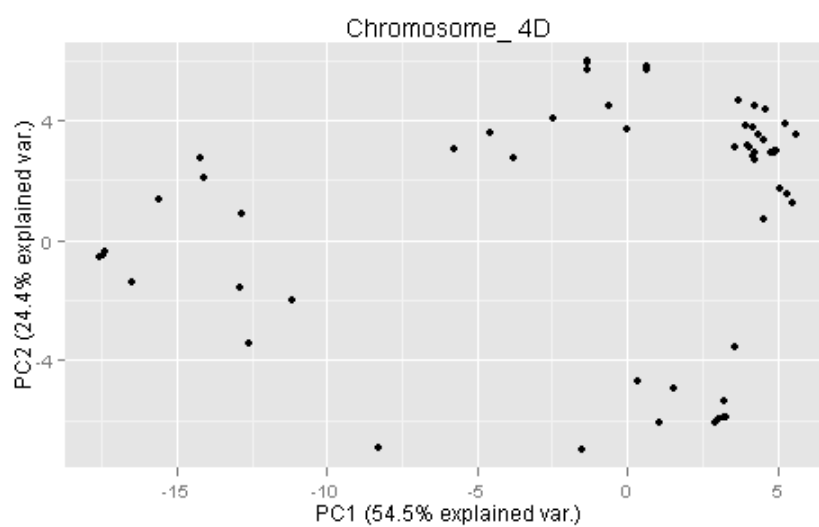

5A

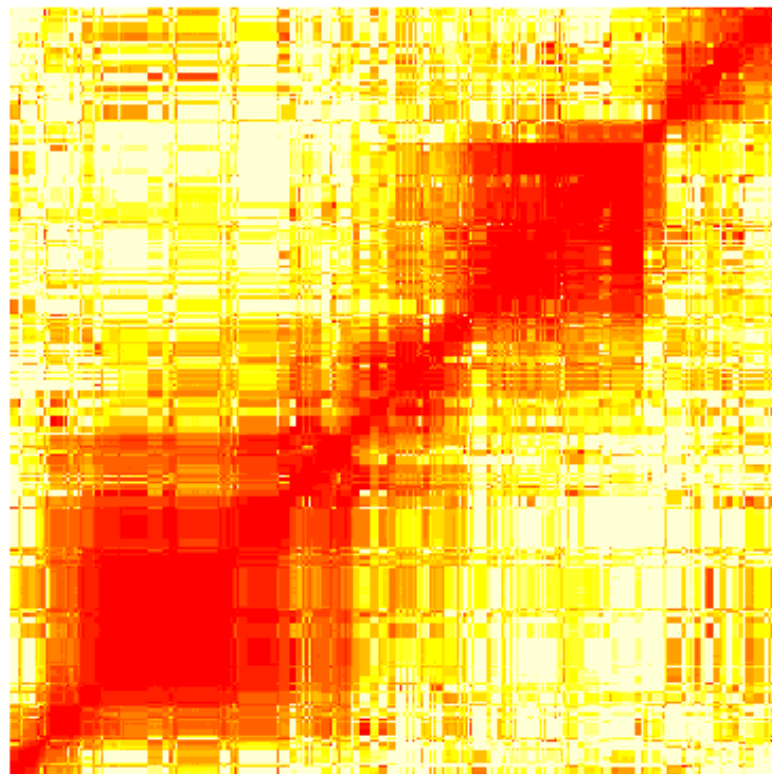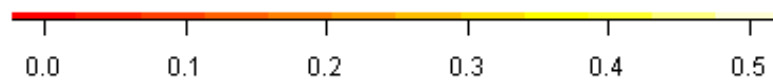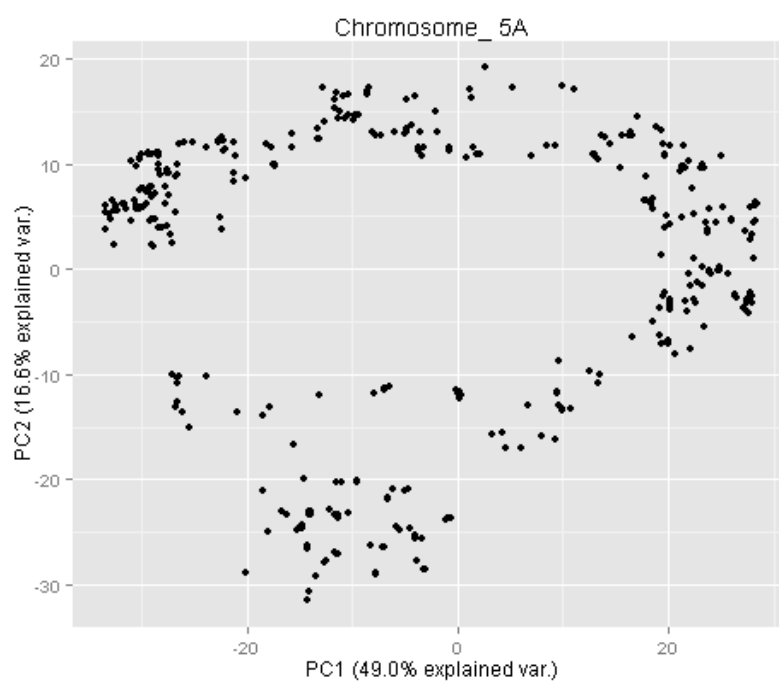

5B

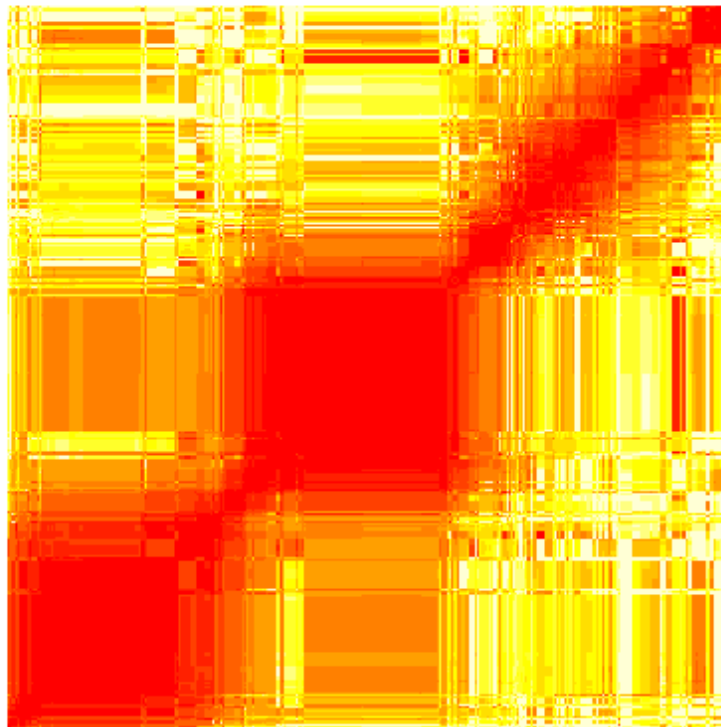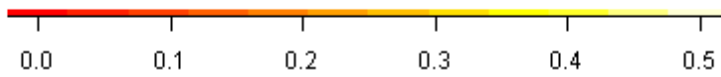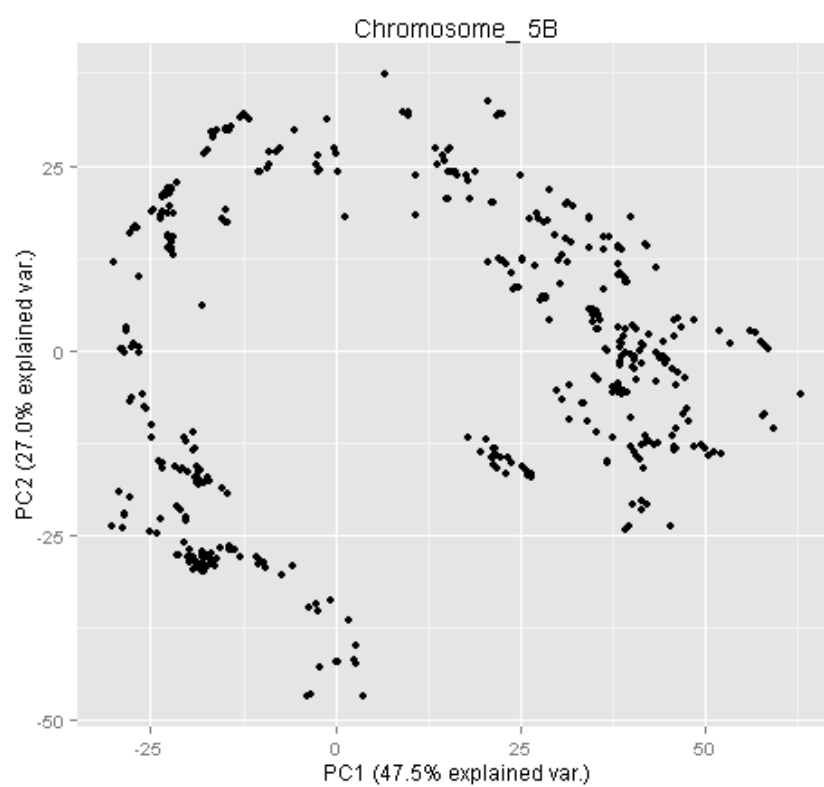

# 5D

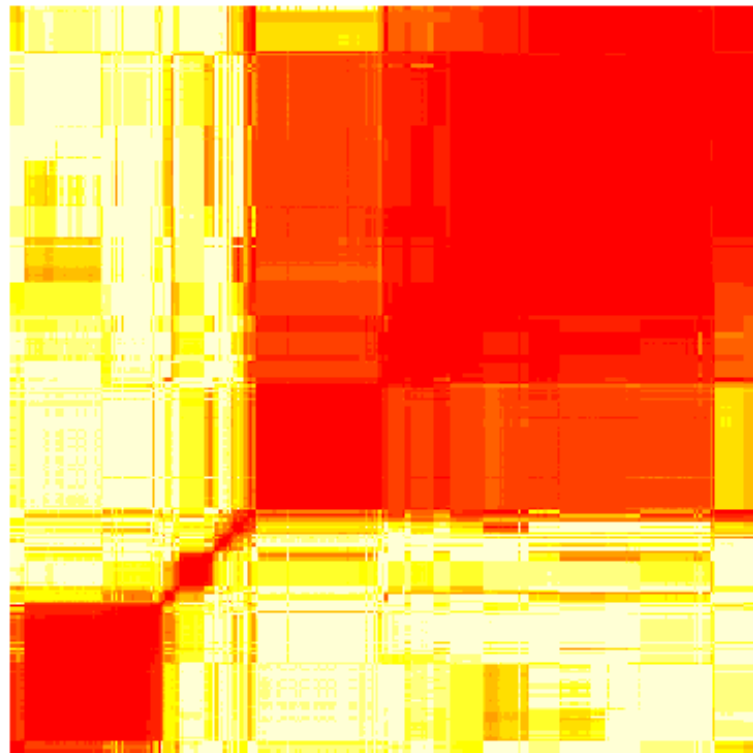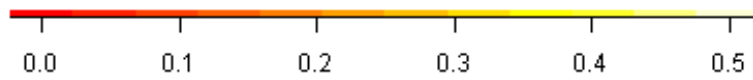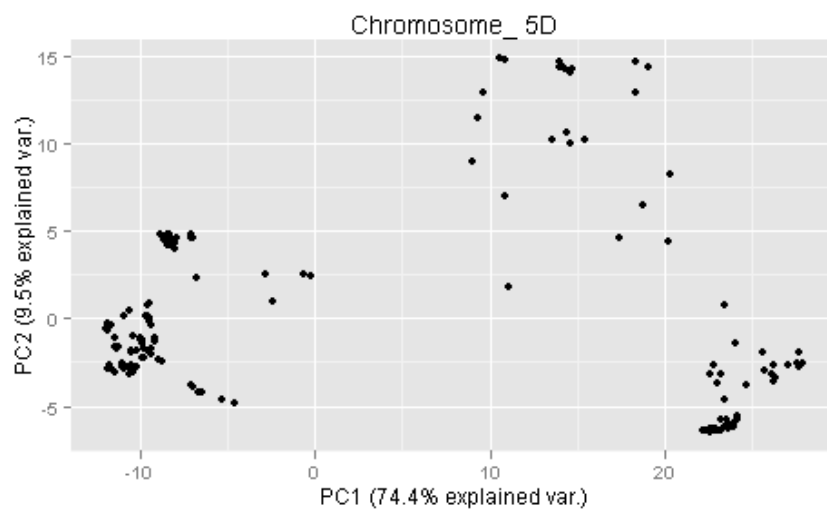

6A

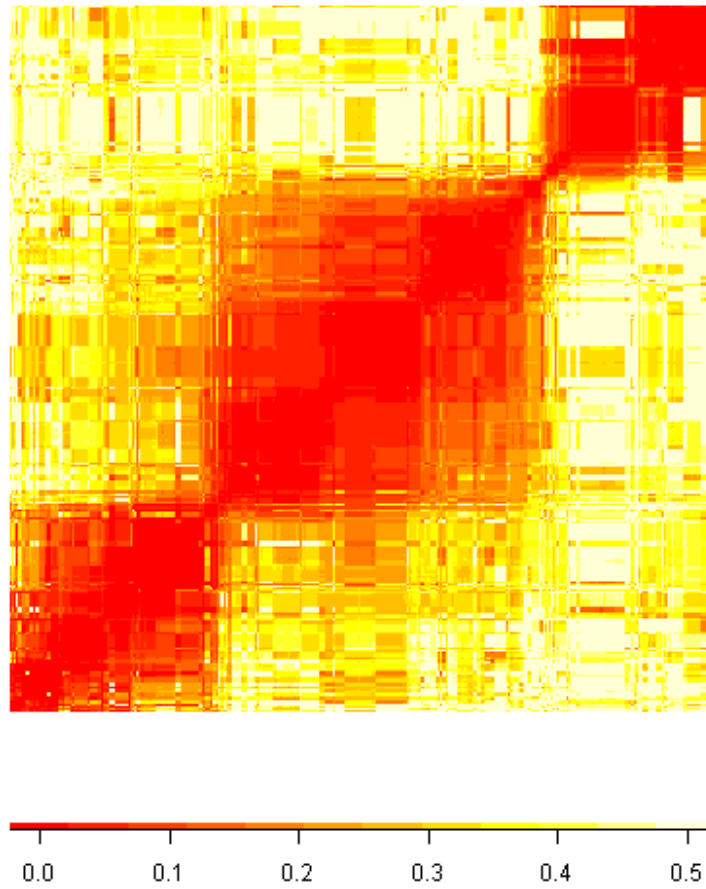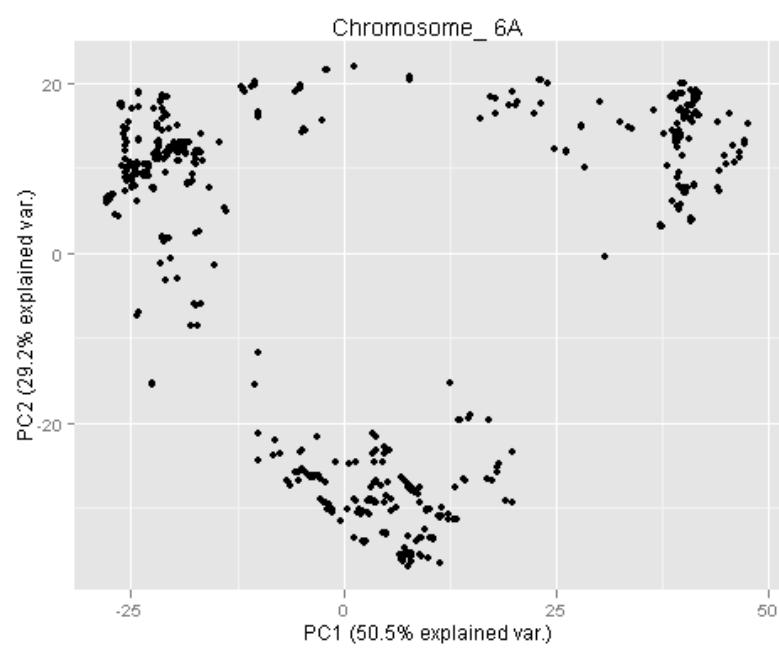

6B

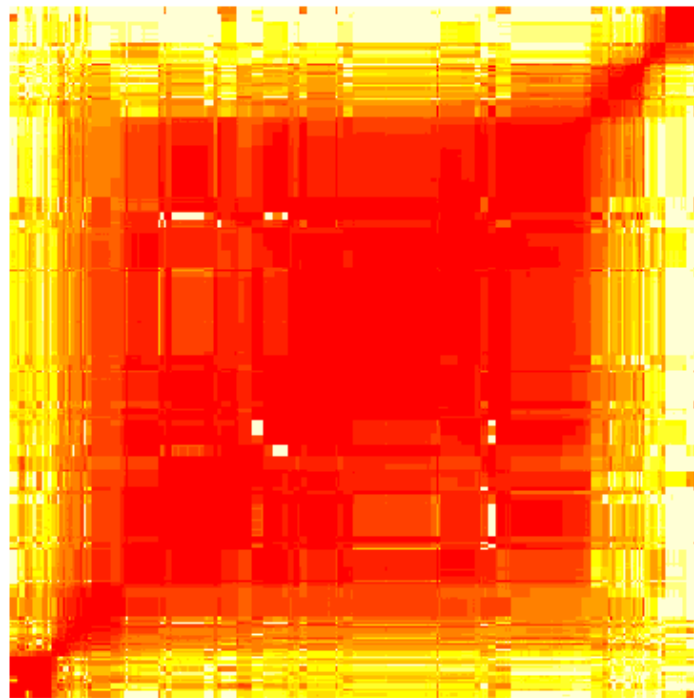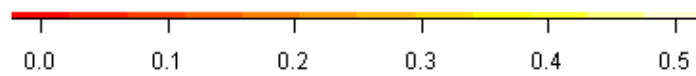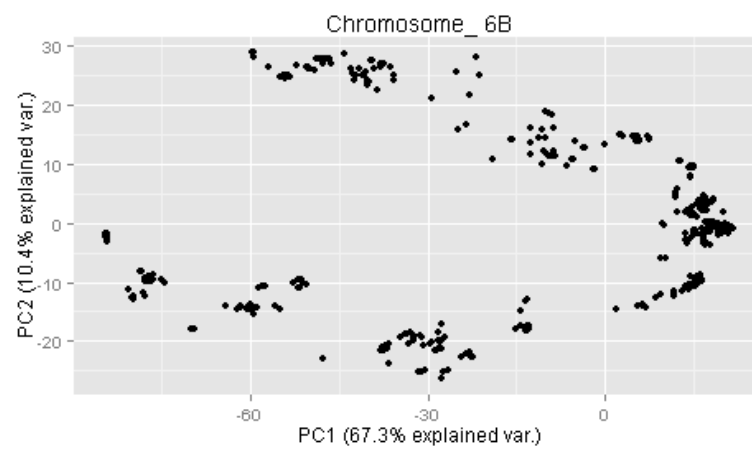

# 6D

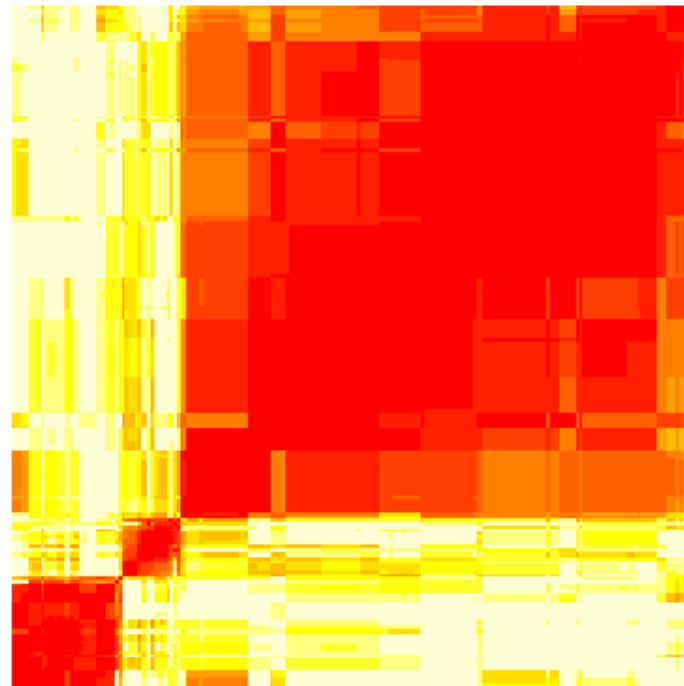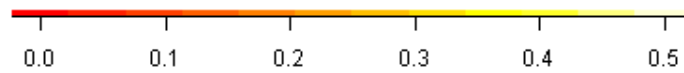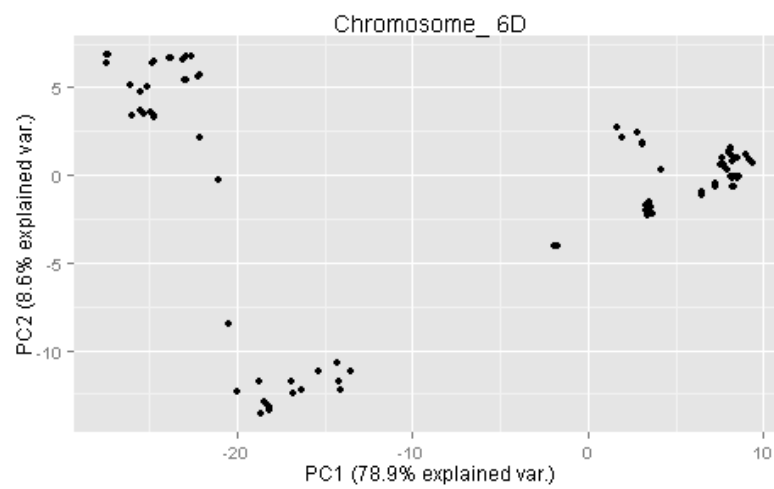

# 7A

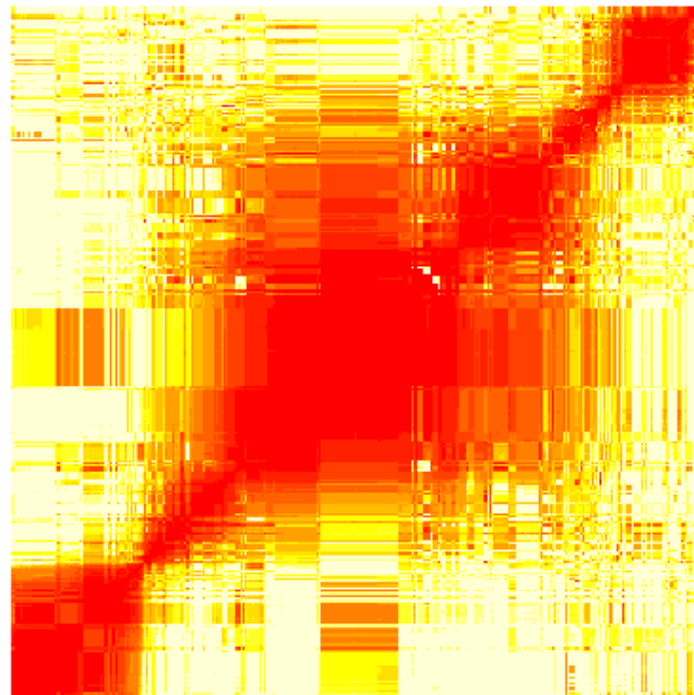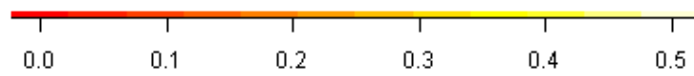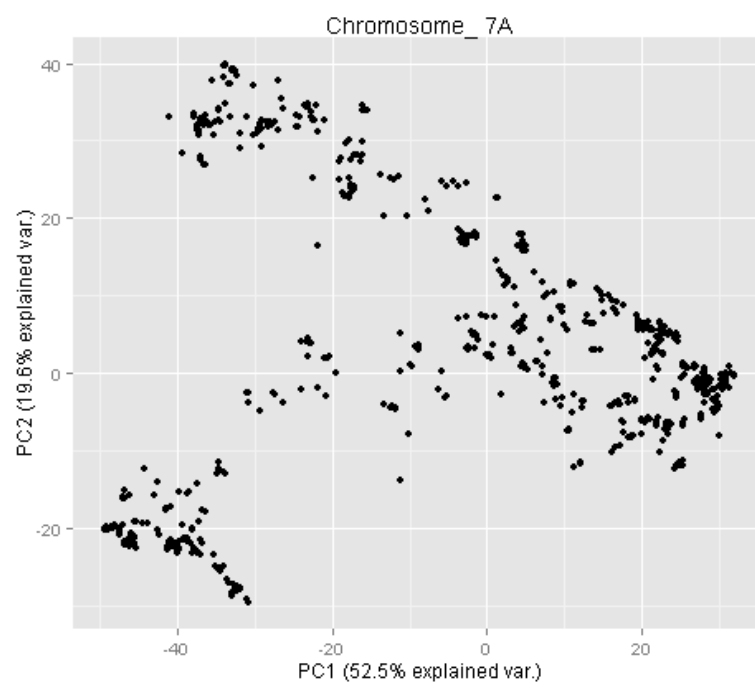

7B

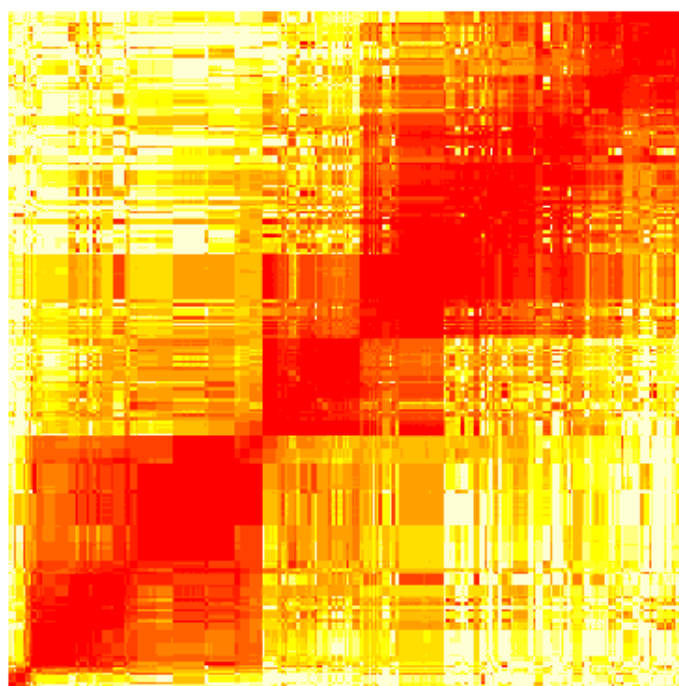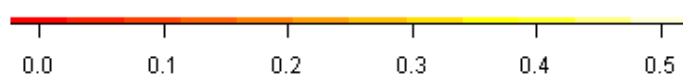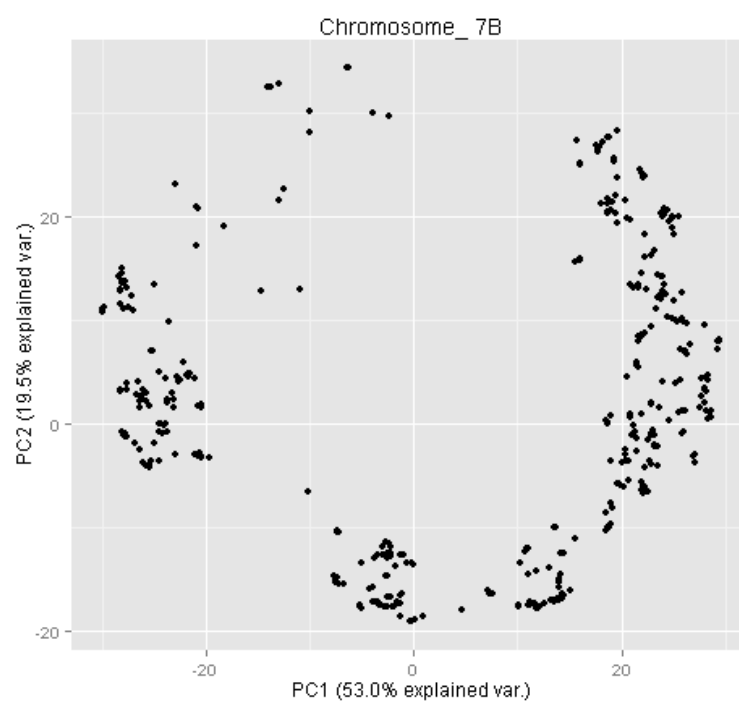

# 7D

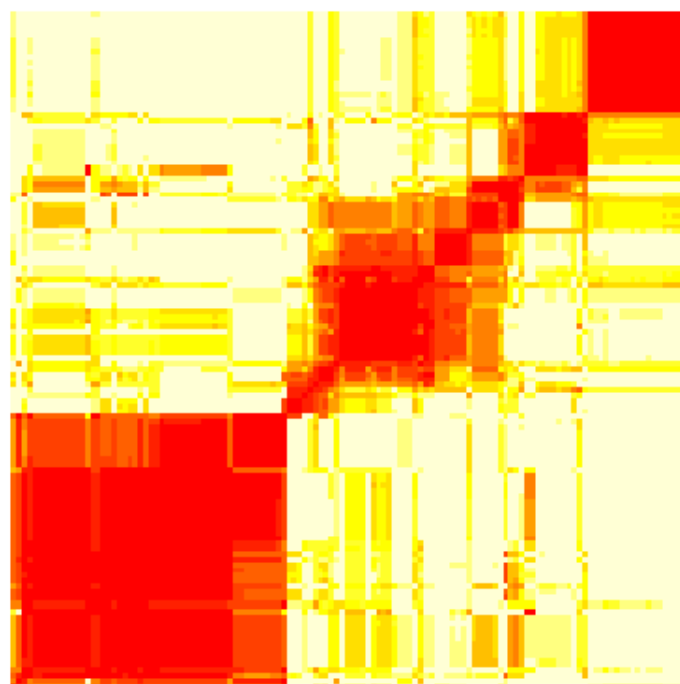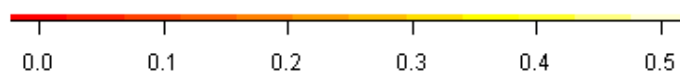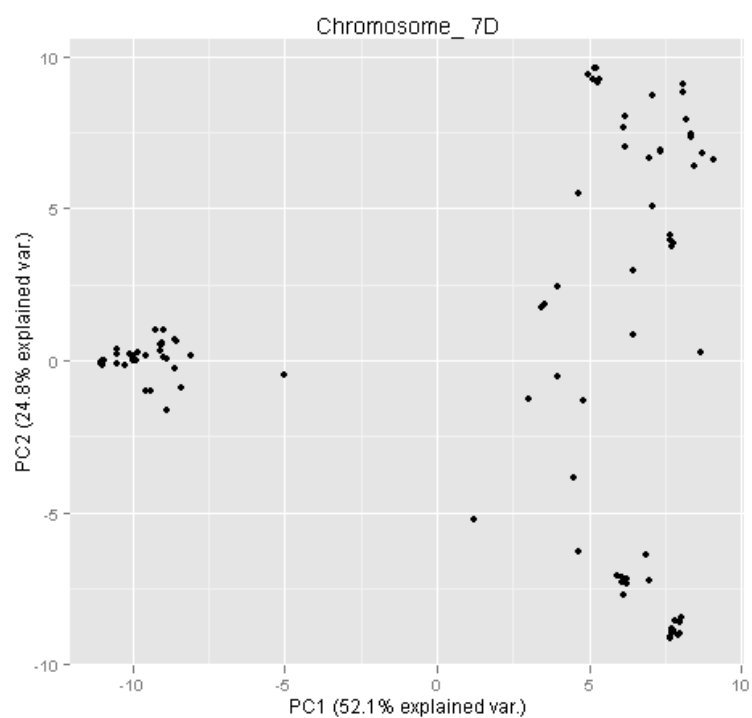

Supplement: Supplementary file 1 — Figure S1 Heat maps of individual chromosomes in NIAB2015. [file PBI-14-1406-s005.pdf]
