# Supplementary material for: A highly recombined, high‐density, eight‐founder wheat MAGIC map reveals extensive segregation distortion and genomic locations of introgression segments
Source: Plant Biotechnol J. 2016 Jan 23;14(6):1406–17. doi: 10.1111/pbi.12504 (PMC4985697; doi:10.1111/pbi.12504)
Supplement: Supplementary file 2 — Figure S2 Graphical comparison of all chromosomes to four genetic maps. [file PBI-14-1406-s004.pdf]

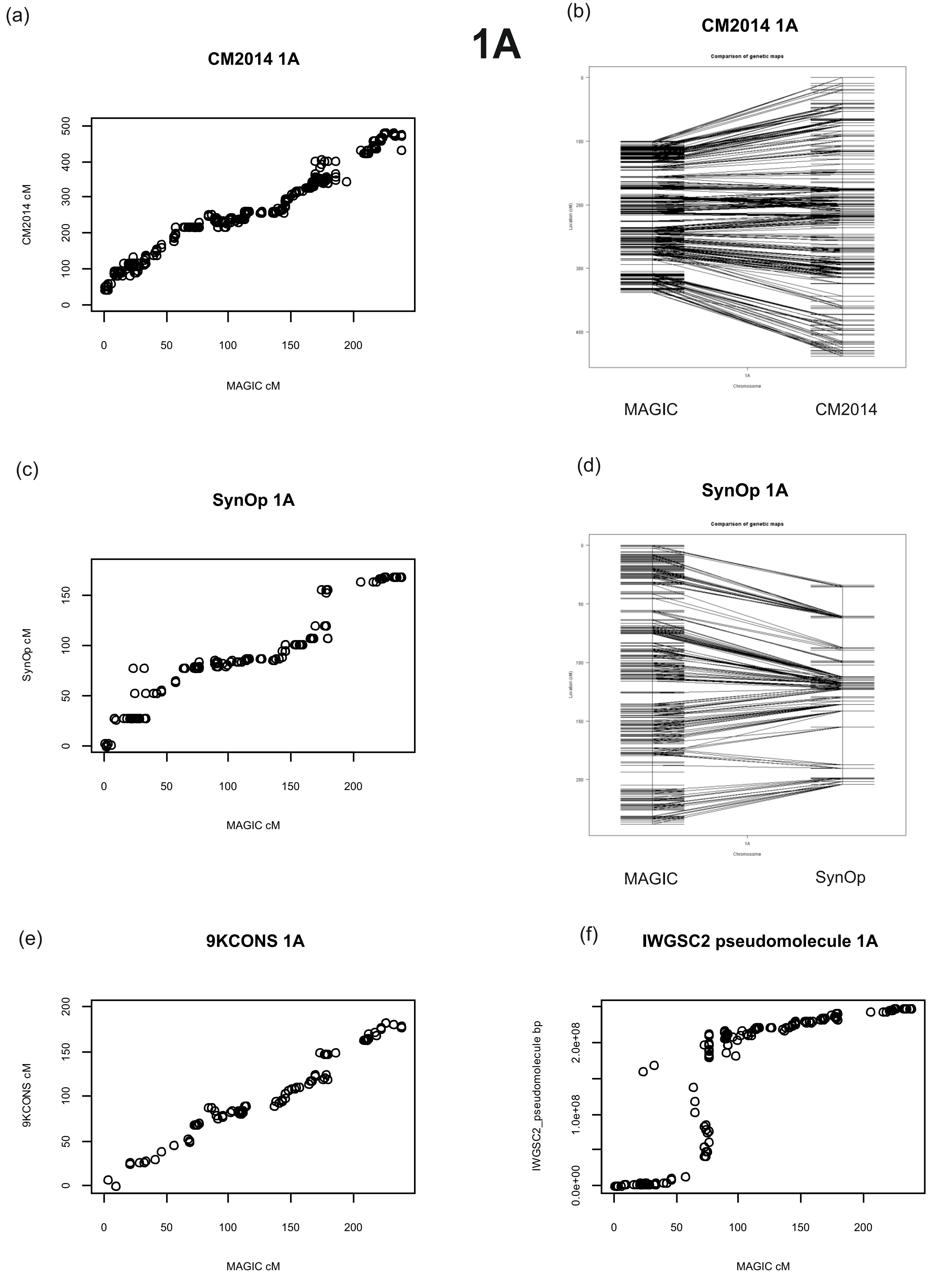
1A
(a)
CM2014 1A
CM2014 cM
MAGIC cM
(b)
CM2014 1A
Comparison of genetic maps
Location (cM)
1A
Chromosome
MAGIC
CM2014
(c)
SynOp 1A
SynOp cM
MAGIC cM
(d)
SynOp 1A
Comparison of genetic maps
Location (cM)
1A
Chromosome
MAGIC
SynOp
(e)
9KCONS 1A
9KCONS cM
MAGIC cM
(f)
IWGSC2 pseudomolecule 1A
IWGSC2_pseudomolecule bp
MAGIC cM

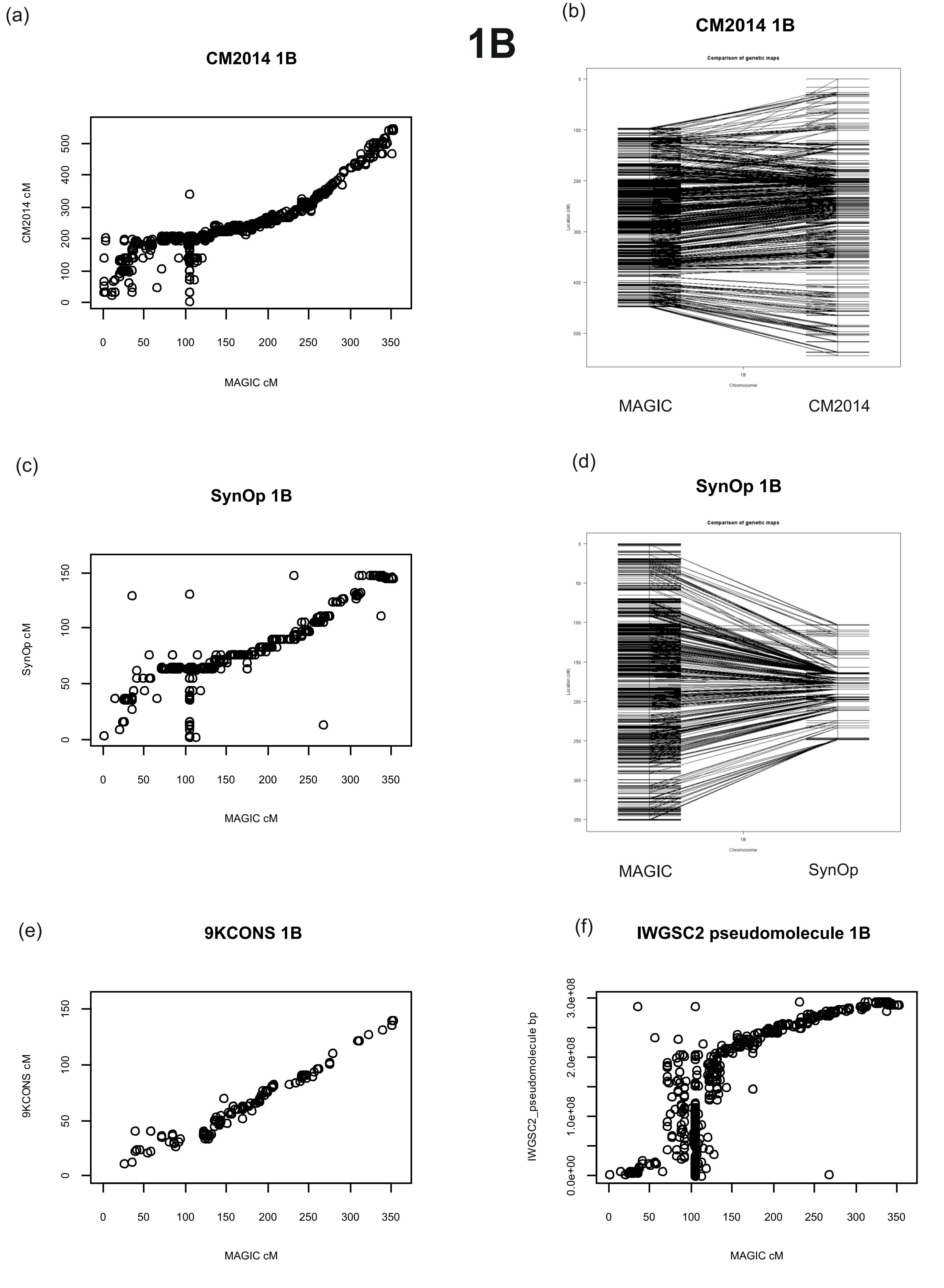
1B
(a)
CM2014 1B
CM2014 cM
MAGIC cM
(b)
CM2014 1B
Comparison of genetic maps
Location (cM)
Chromosome
MAGIC
CM2014
(c)
SynOp 1B
SynOp cM
MAGIC cM
(d)
SynOp 1B
Comparison of genetic maps
Location (cM)
Chromosome
MAGIC
SynOp
(e)
9KCONS 1B
9KCONS cM
MAGIC cM
(f)
IWGSC2 pseudomolecule 1B
IWGSC2_pseudomolecule bp
MAGIC cM

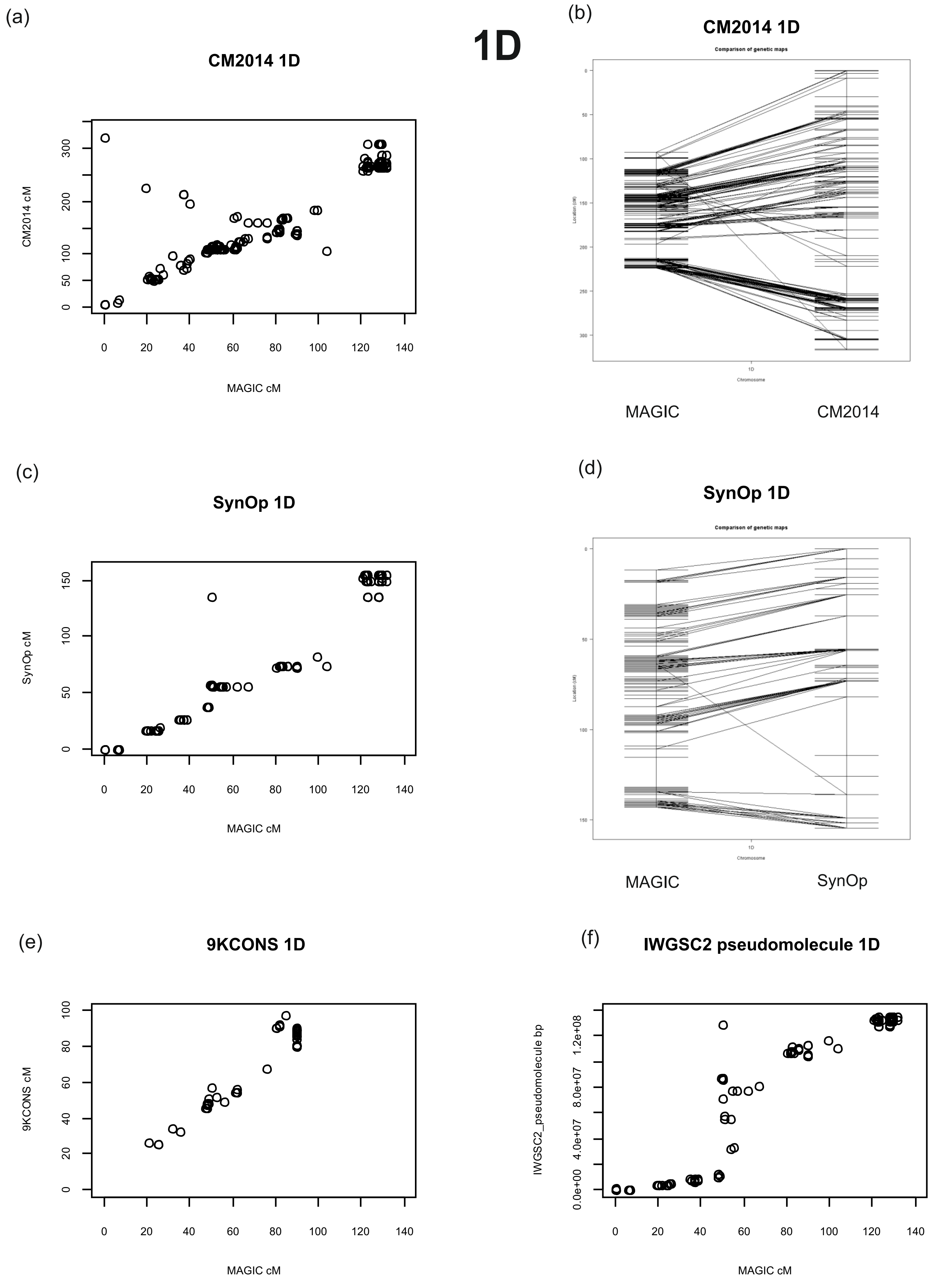
1D
(a)
CM2014 1D
CM2014 cM
MAGIC cM
0
50
100
200
300
0
20
40
60
80
100
120
140
(b)
CM2014 1D
Comparison of genetic maps
Location (cM)
Chromosome
1D
MAGIC
CM2014
(c)
SynOp 1D
SynOp cM
MAGIC cM
(d)
SynOp 1D
Comparison of genetic maps
Location (cM)
Chromosome
1D
MAGIC
SynOp
(e)
9KCONS 1D
9KCONS cM
MAGIC cM
(f)
IWGSC2 pseudomolecule 1D
IWGSC2_pseudomolecule bp
0.0e+00
4.0e+07
8.0e+07
1.2e+08
MAGIC cM

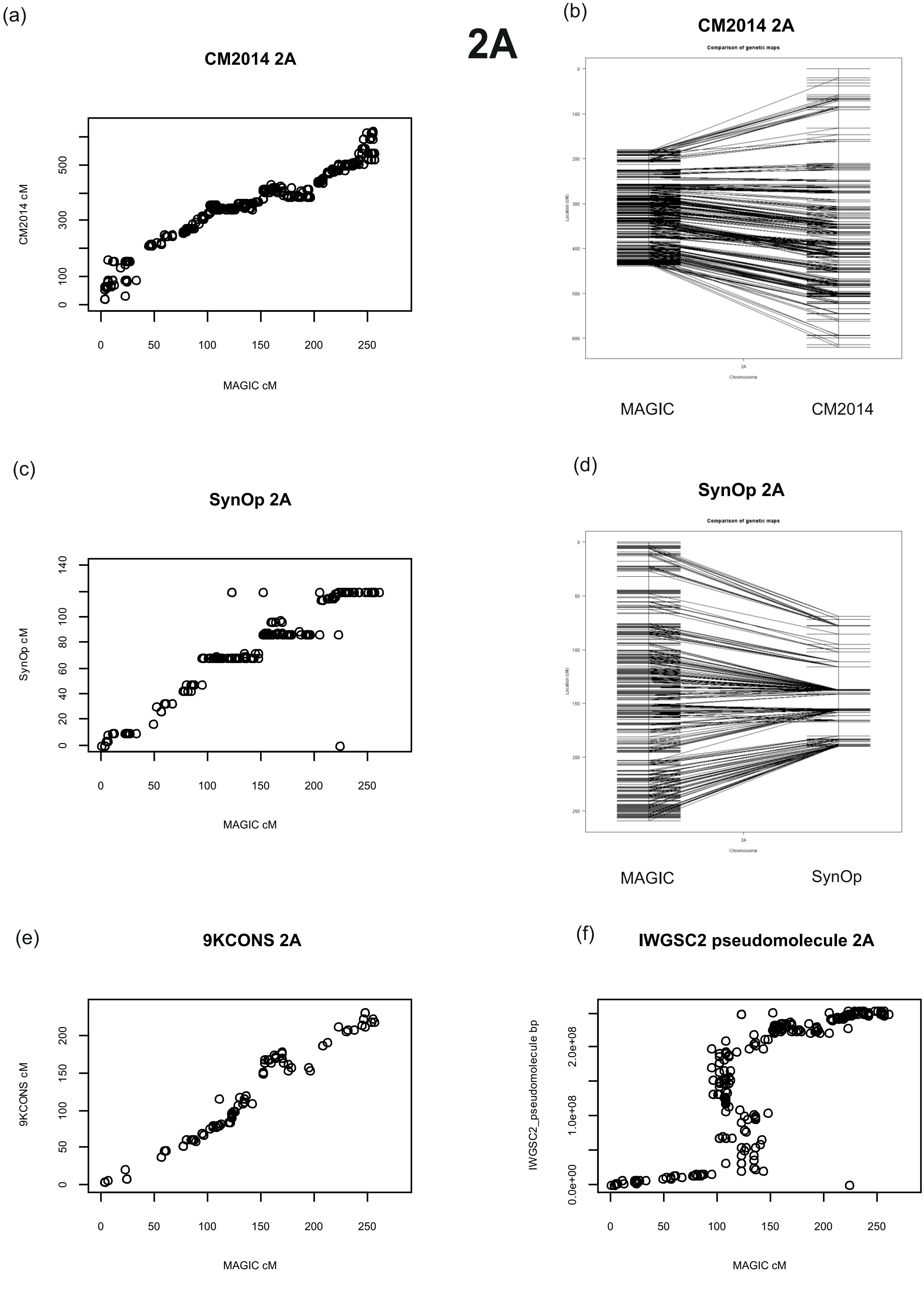
2A
(a)
CM2014 2A
CM2014 cM
MAGIC cM
(b)
CM2014 2A
Comparison of genetic maps
MAGIC
CM2014
(c)
SynOp 2A
SynOp cM
MAGIC cM
(d)
SynOp 2A
Comparison of genetic maps
MAGIC
SynOp
(e)
9KCONS 2A
9KCONS cM
MAGIC cM
(f)
IWGSC2 pseudomolecule 2A
IWGSC2_pseudomolecule bp
MAGIC cM

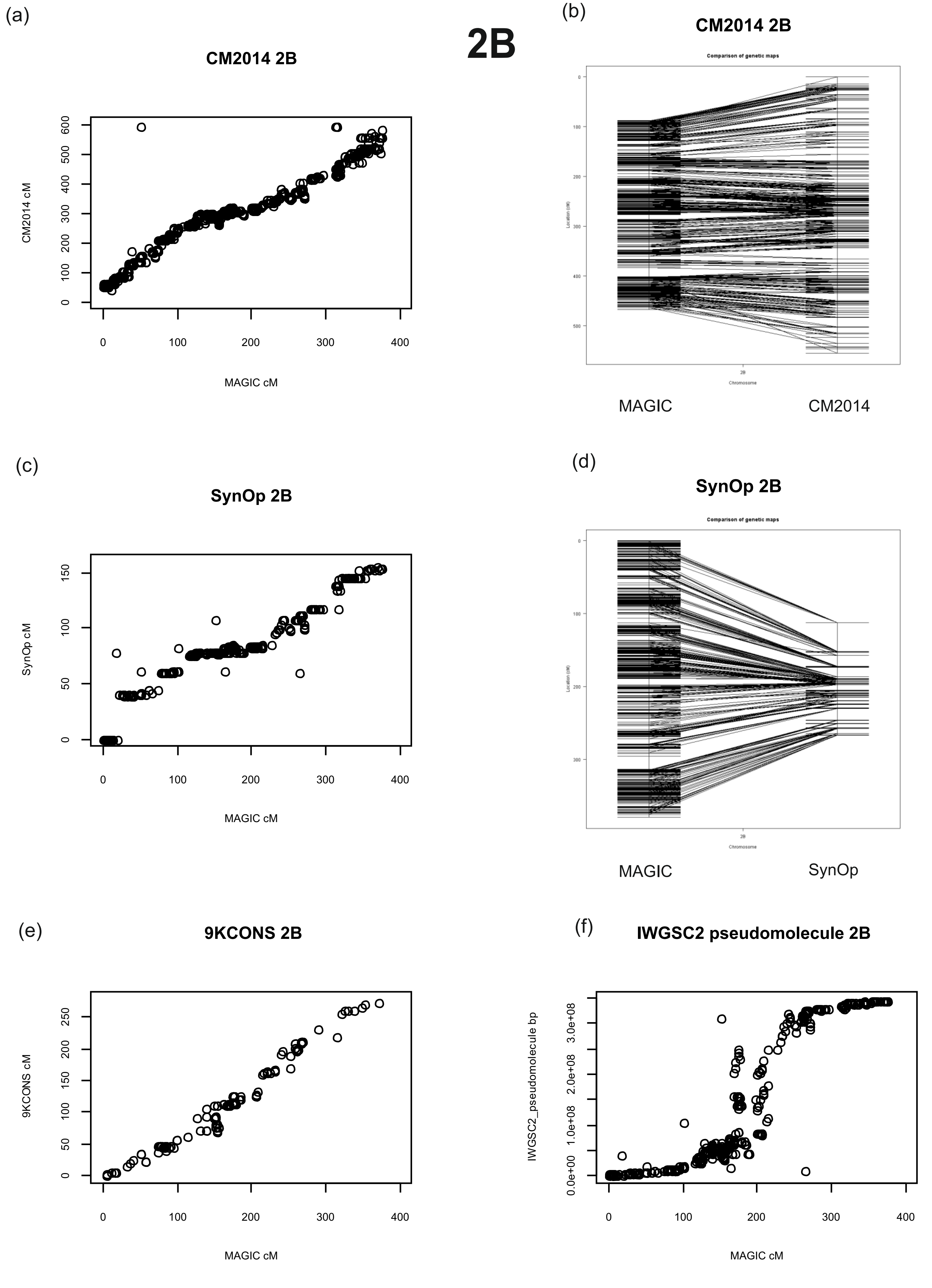

2B
(a)
CM2014 2B
CM2014 cM
MAGIC cM
(b)
CM2014 2B
Comparison of genetic maps
MAGIC
CM2014
(c)
SynOp 2B
SynOp cM
MAGIC cM
(d)
SynOp 2B
Comparison of genetic maps
MAGIC
SynOp
(e)
9KCONS 2B
9KCONS cM
MAGIC cM
(f)
IWGSC2 pseudomolecule 2B
IWGSC2_pseudomolecule bp
MAGIC cM

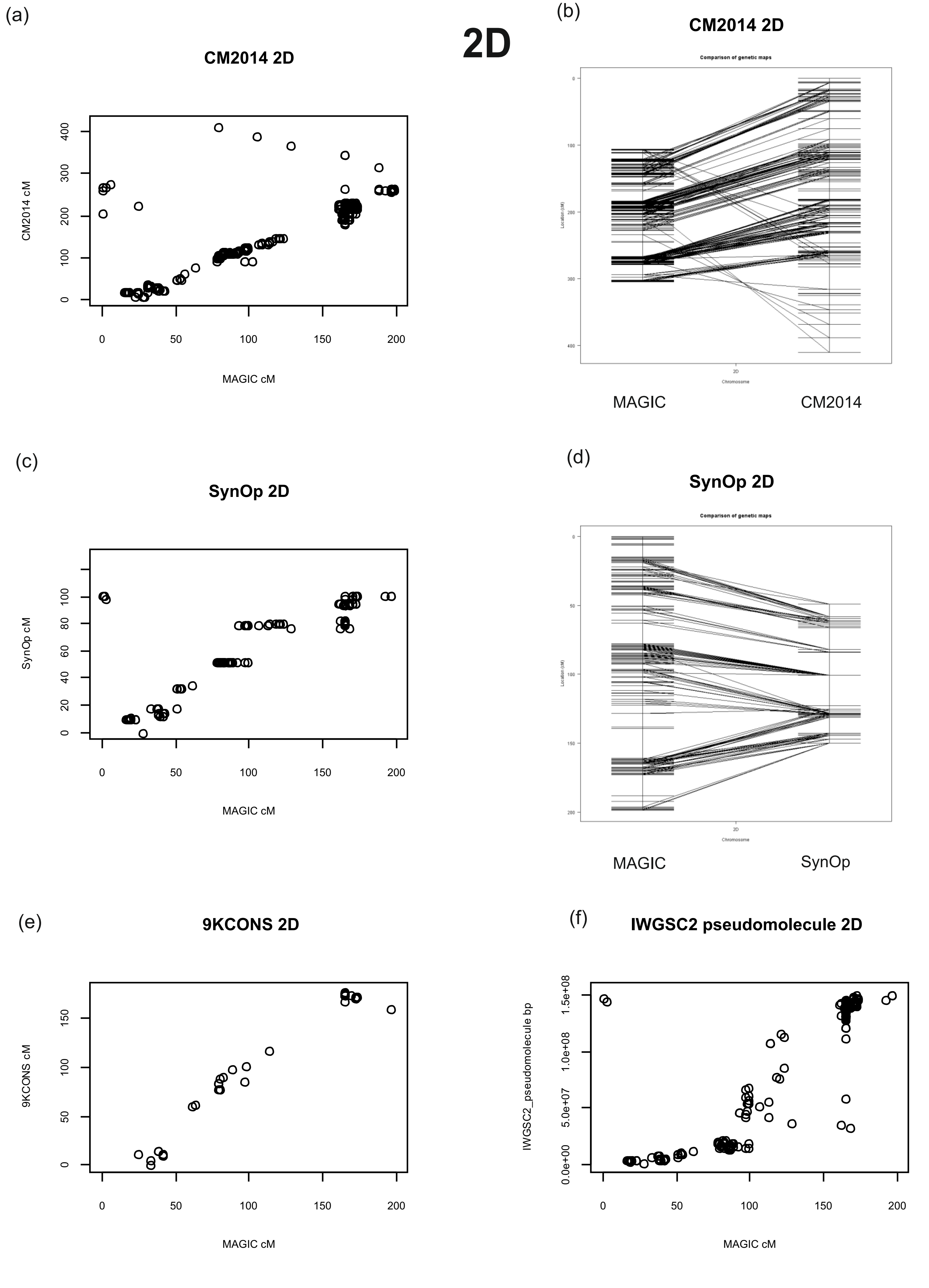
2D
(a)
CM2014 2D
CM2014 cM
MAGIC cM
(b)
CM2014 2D
Comparison of genetic maps
Location (cM)
Chromosome
MAGIC
CM2014
(c)
SynOp 2D
SynOp cM
MAGIC cM
(d)
SynOp 2D
Comparison of genetic maps
Location (cM)
Chromosome
MAGIC
SynOp
(e)
9KCONS 2D
9KCONS cM
MAGIC cM
(f)
IWGSC2 pseudomolecule 2D
IWGSC2_pseudomolecule bp
MAGIC cM

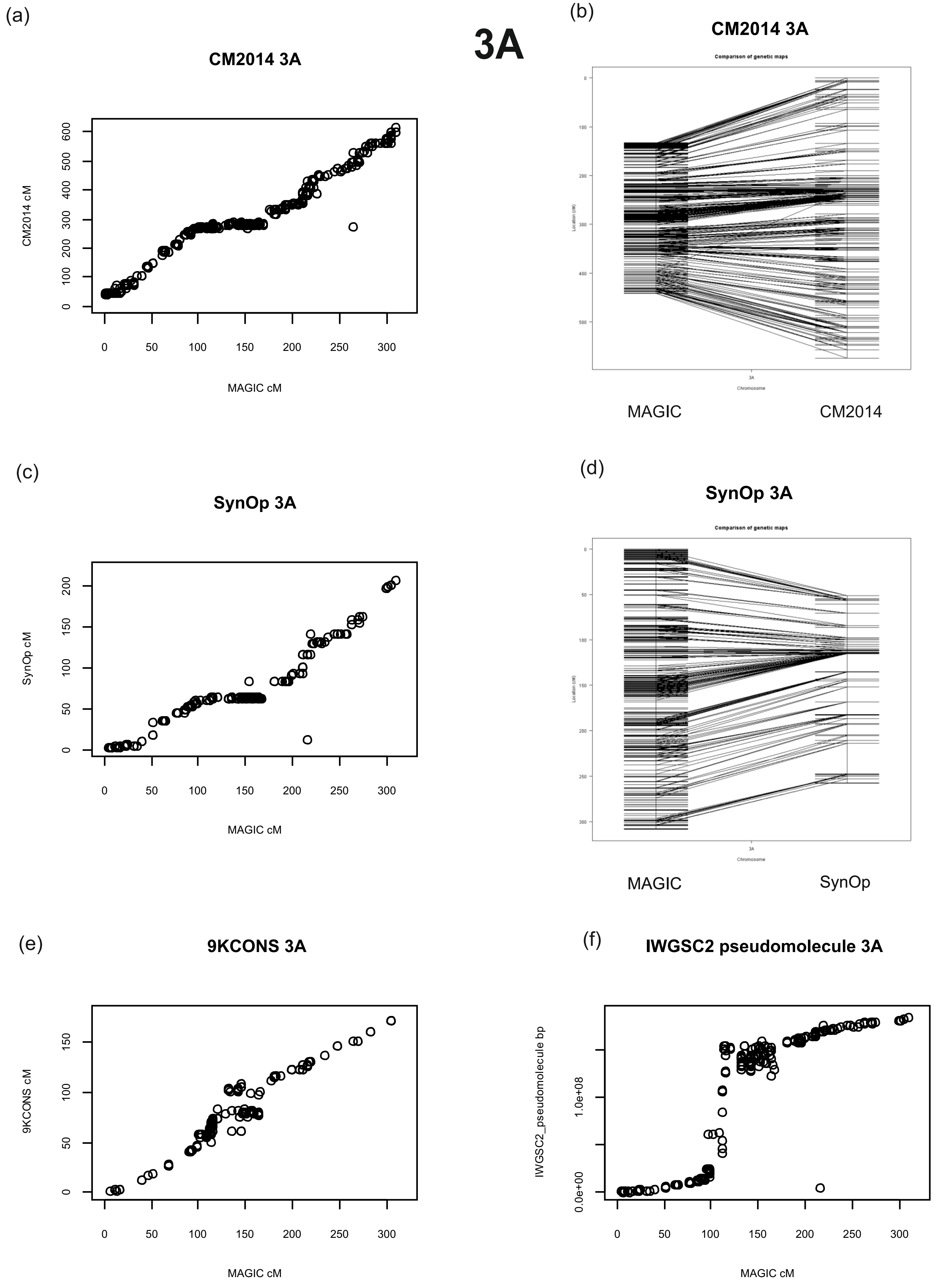
3A
(a)
CM2014 3A
CM2014 cM
MAGIC cM
(b)
CM2014 3A
Comparison of genetic maps
Location (cM)
Chromosome
MAGIC
CM2014
(c)
SynOp 3A
SynOp cM
MAGIC cM
(d)
SynOp 3A
Comparison of genetic maps
Location (cM)
Chromosome
MAGIC
SynOp
(e)
9KCONS 3A
9KCONS cM
MAGIC cM
(f)
IWGSC2 pseudomolecule 3A
IWGSC2_pseudomolecule bp
MAGIC cM

3B

(a)

CM2014 3B

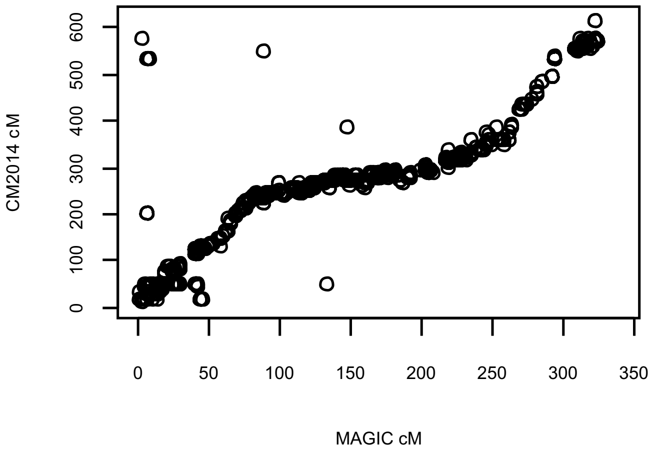

(b)

CM2014 3B

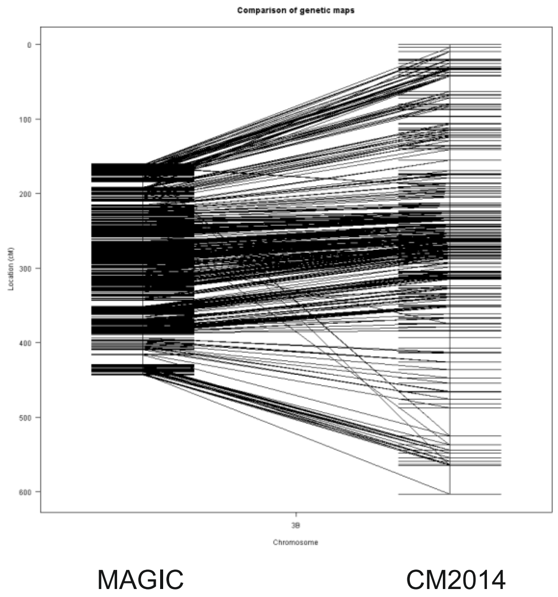

(c)

SynOp 3B

For 3B comparison to Synop and SynOp derived pseudomolecule, see physical map comparison in main text

(d)

SynOp 3B

For 3B comparison to Synop and SynOp derived pseudomolecule, see physical map comparison in main text

MAGIC SynOp

(e)

9KCONS 3B

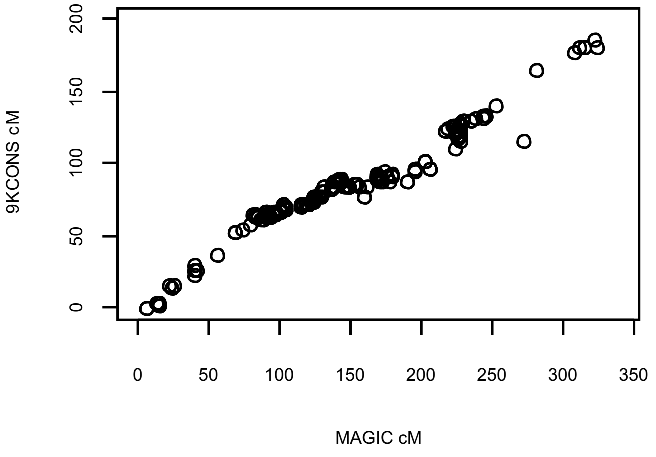

(f)

For 3B comparison to Synop and SynOp derived pseudomolecule, see physical map comparison in main text

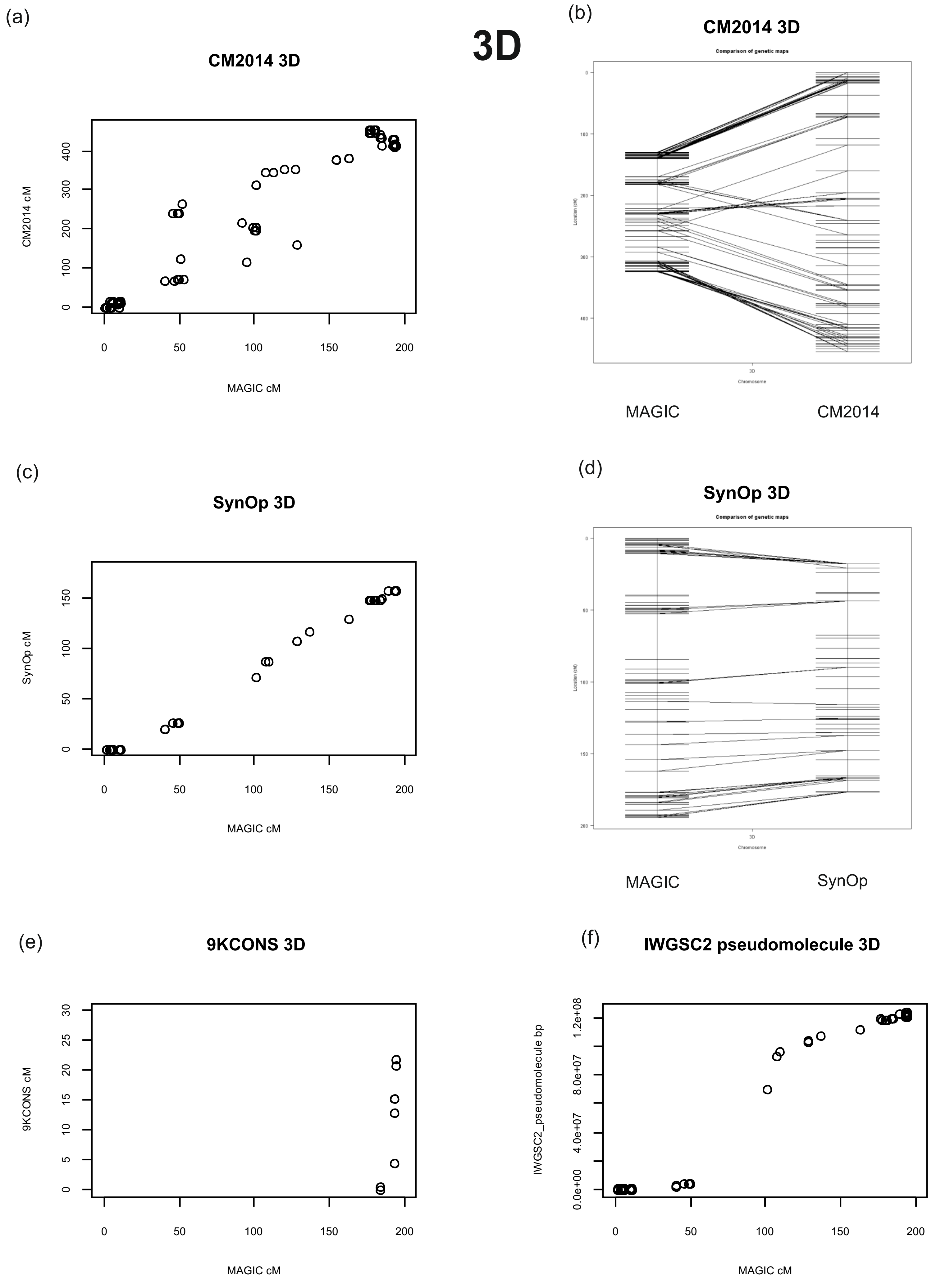

3D
(a)
CM2014 3D
CM2014 cM
MAGIC cM
(b)
CM2014 3D
Comparison of genetic maps
Location (cM)
3D
Chromosome
MAGIC
CM2014
(c)
SynOp 3D
SynOp cM
MAGIC cM
(d)
SynOp 3D
Comparison of genetic maps
Location (cM)
3D
Chromosome
MAGIC
SynOp
(e)
9KCONS 3D
9KCONS cM
MAGIC cM
(f)
IWGSC2 pseudomolecule 3D
IWGSC2_pseudomolecule bp
MAGIC cM

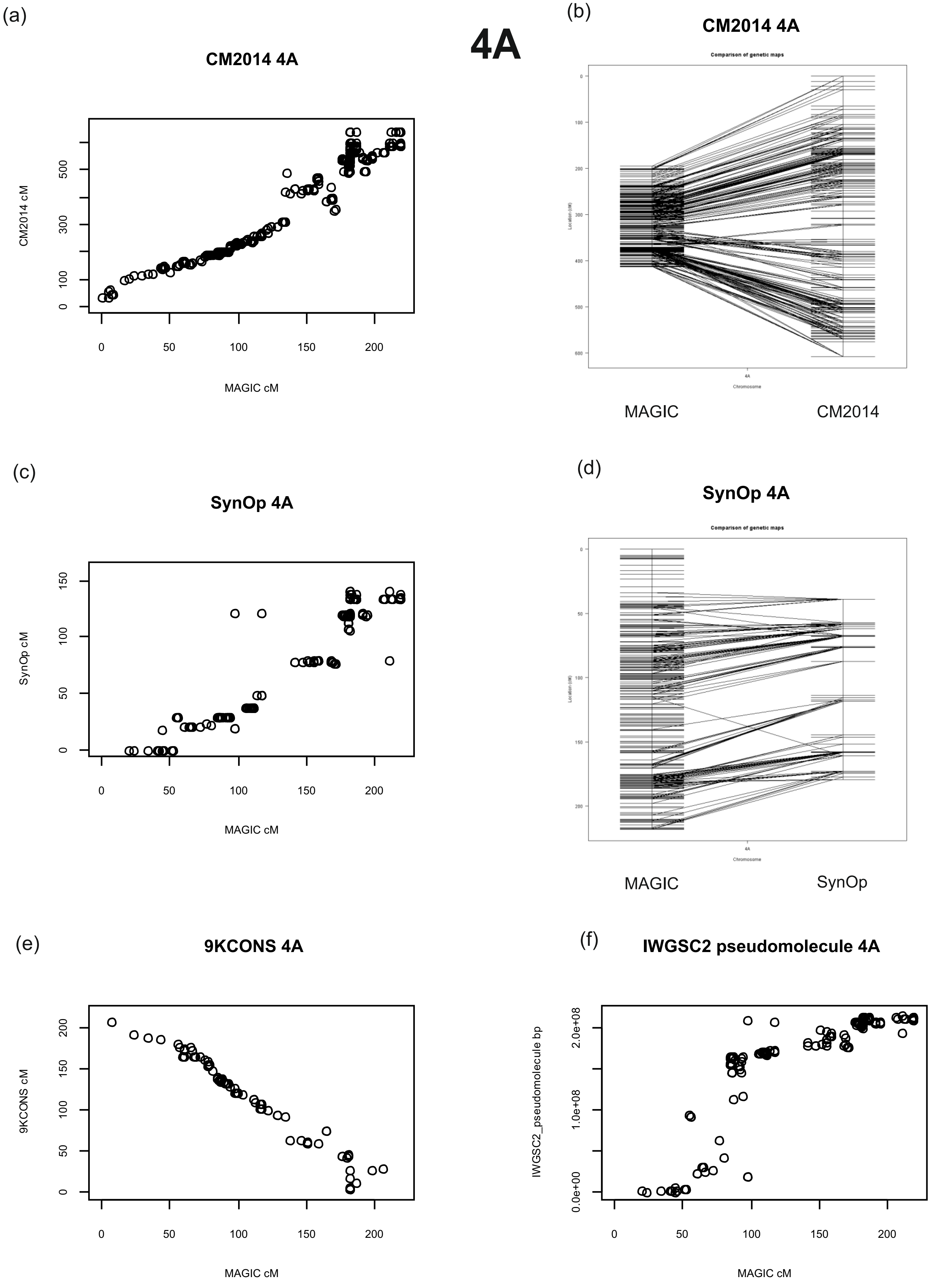

4A
(a)
CM2014 4A
CM2014 cM
MAGIC cM
0
100
300
500
0
50
100
150
200
(b)
CM2014 4A
Comparison of genetic maps
MAGIC
CM2014
(c)
SynOp 4A
SynOp cM
MAGIC cM
0
50
100
150
0
50
100
150
200
(d)
SynOp 4A
Comparison of genetic maps
MAGIC
SynOp
(e)
9KCONS 4A
9KCONS cM
MAGIC cM
0
50
100
150
200
0
50
100
150
200
(f)
IWGSC2 pseudomolecule 4A
IWGSC2_pseudomolecule bp
MAGIC cM
0.0e+00
1.0e+08
2.0e+08
0
50
100
150
200

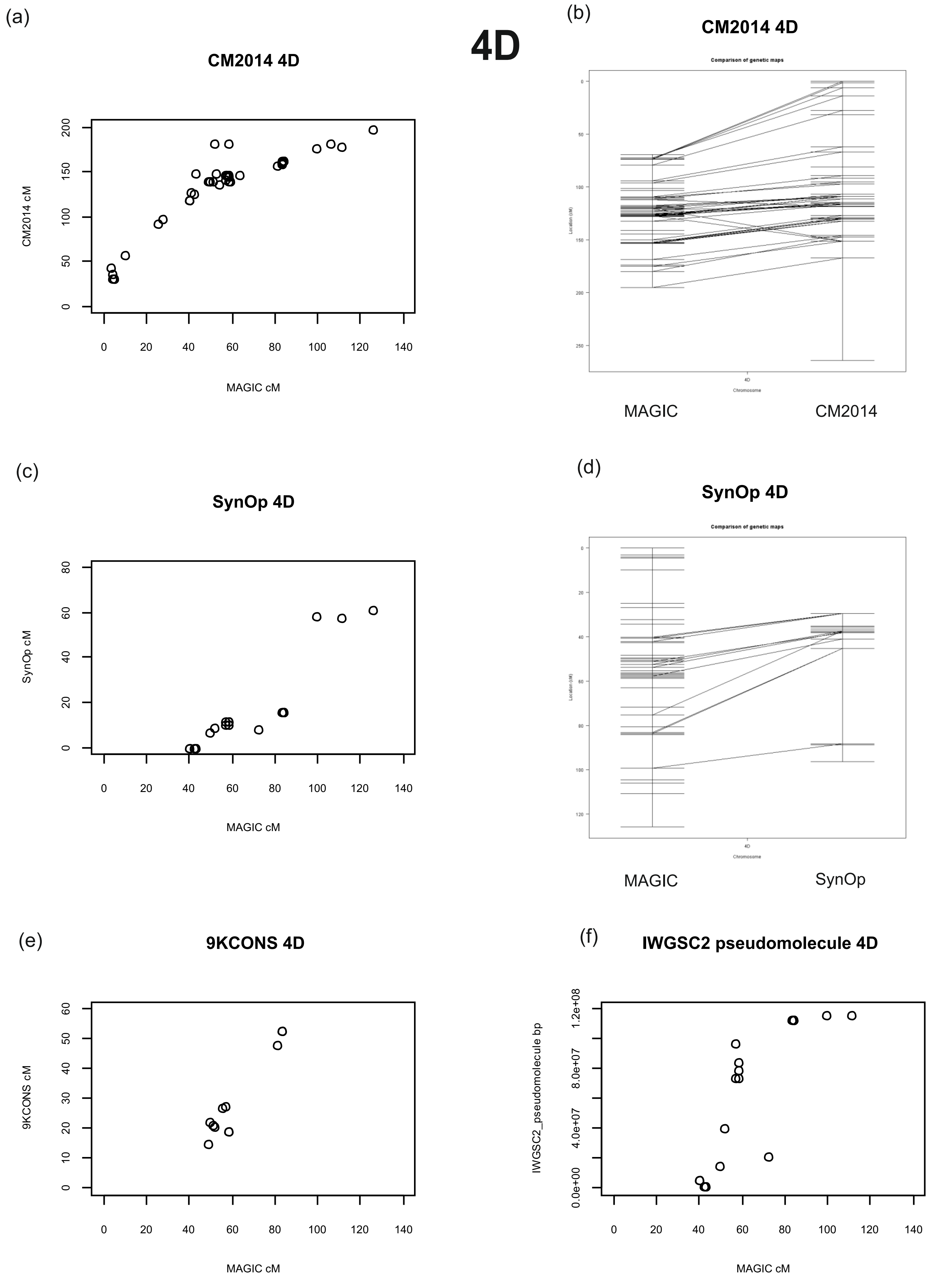
4D
(a)
CM2014 4D
CM2014 cM
MAGIC cM
(b)
CM2014 4D
Comparison of genetic maps
MAGIC
CM2014
(c)
SynOp 4D
SynOp cM
MAGIC cM
(d)
SynOp 4D
Comparison of genetic maps
MAGIC
SynOp
(e)
9KCONS 4D
9KCONS cM
MAGIC cM
(f)
IWGSC2 pseudomolecule 4D
IWGSC2_pseudomolecule bp
MAGIC cM

4B

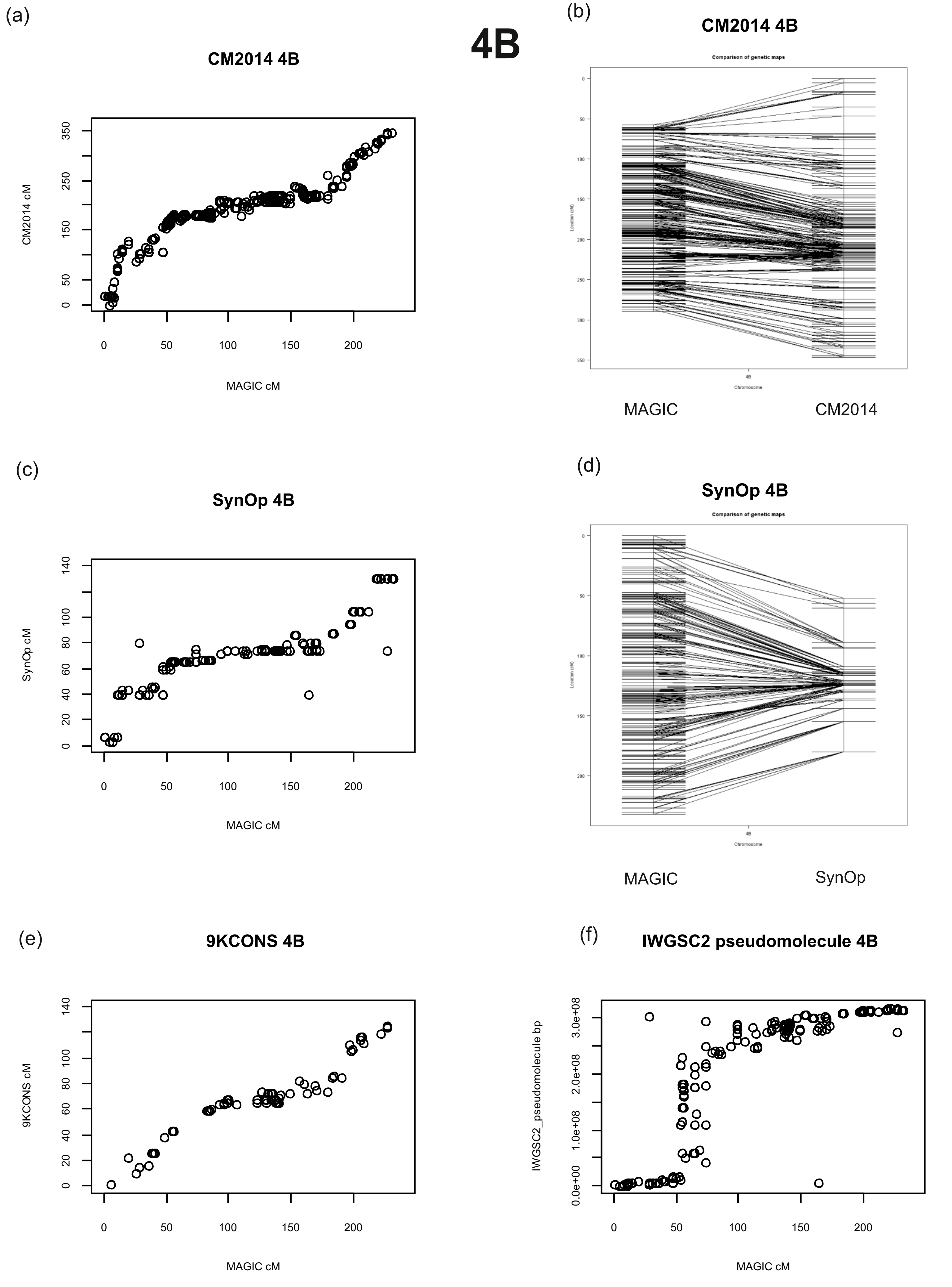

(a)
CM2014 4B
CM2014 cM
MAGIC cM
(b)
CM2014 4B
Comparison of genetic maps
Location (cM)
Chromosome
MAGIC
CM2014
(c)
SynOp 4B
SynOp cM
MAGIC cM
(d)
SynOp 4B
Comparison of genetic maps
Location (cM)
Chromosome
MAGIC
SynOp
(e)
9KCONS 4B
9KCONS cM
MAGIC cM
(f)
IWGSC2 pseudomolecule 4B
IWGSC2_pseudomolecule bp
MAGIC cM

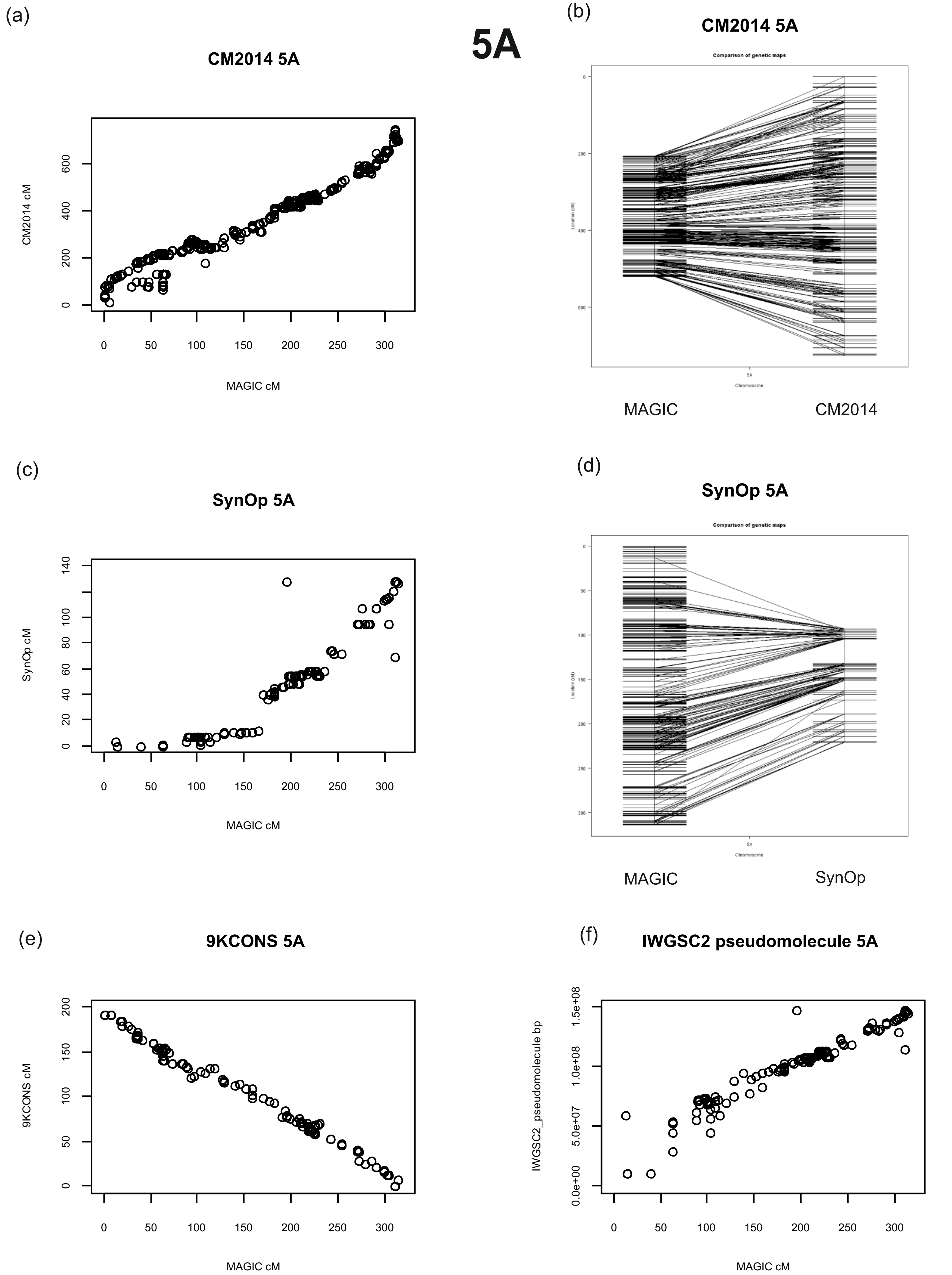

(a)
5A
CM2014 5A
CM2014 cM
MAGIC cM
(b)
CM2014 5A
Comparison of genetic maps
MAGIC
CM2014
(c)
SynOp 5A
SynOp cM
MAGIC cM
(d)
SynOp 5A
Comparison of genetic maps
MAGIC
SynOp
(e)
9KCONS 5A
9KCONS cM
MAGIC cM
(f)
IWGSC2 pseudomolecule 5A
IWGSC2_pseudomolecule bp
MAGIC cM

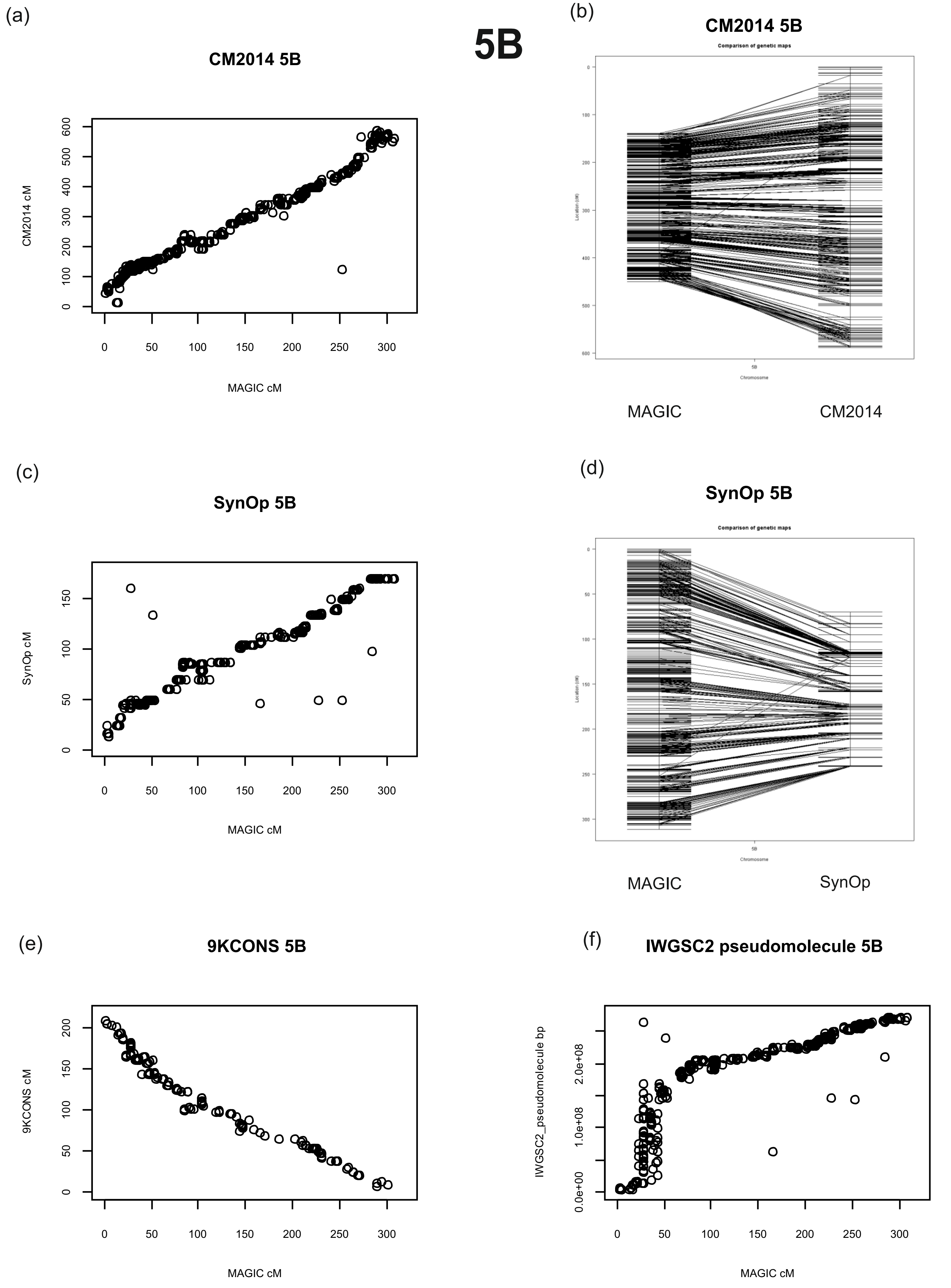

5B
(a)
CM2014 5B
CM2014 cM
MAGIC cM
0
100
200
300
400
500
600
0
50
100
150
200
250
300
(b)
CM2014 5B
Comparison of genetic maps
Location (cM)
Chromosome
MAGIC
CM2014
(c)
SynOp 5B
SynOp cM
MAGIC cM
(d)
SynOp 5B
Comparison of genetic maps
Location (cM)
Chromosome
MAGIC
SynOp
(e)
9KCONS 5B
9KCONS cM
MAGIC cM
(f)
IWGSC2 pseudomolecule 5B
IWGSC2_pseudomolecule bp
0.0e+00
1.0e+08
2.0e+08
MAGIC cM

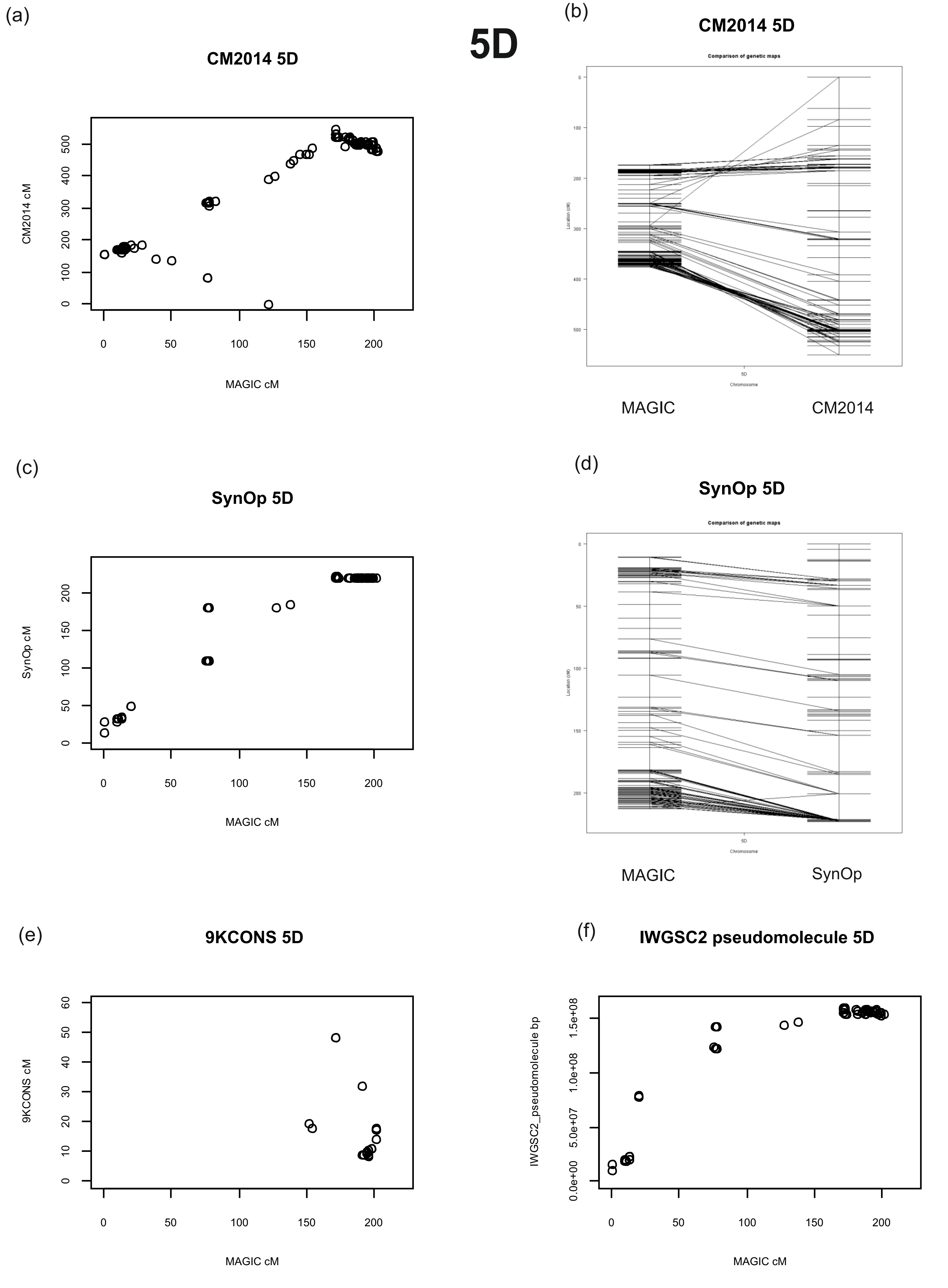
5D
(a)
CM2014 5D
CM2014 cM
MAGIC cM
0
100
200
300
400
500
0
50
100
150
200
(b)
CM2014 5D
Comparison of genetic maps
Location (cM)
Chromosome
MAGIC
CM2014
(c)
SynOp 5D
SynOp cM
MAGIC cM
0
50
100
150
200
(d)
SynOp 5D
Comparison of genetic maps
Location (cM)
Chromosome
MAGIC
SynOp
(e)
9KCONS 5D
9KCONS cM
MAGIC cM
0
10
20
30
40
50
60
(f)
IWGSC2 pseudomolecule 5D
IWGSC2_pseudomolecule bp
MAGIC cM
0.0e+00
5.0e+07
1.0e+08
1.5e+08

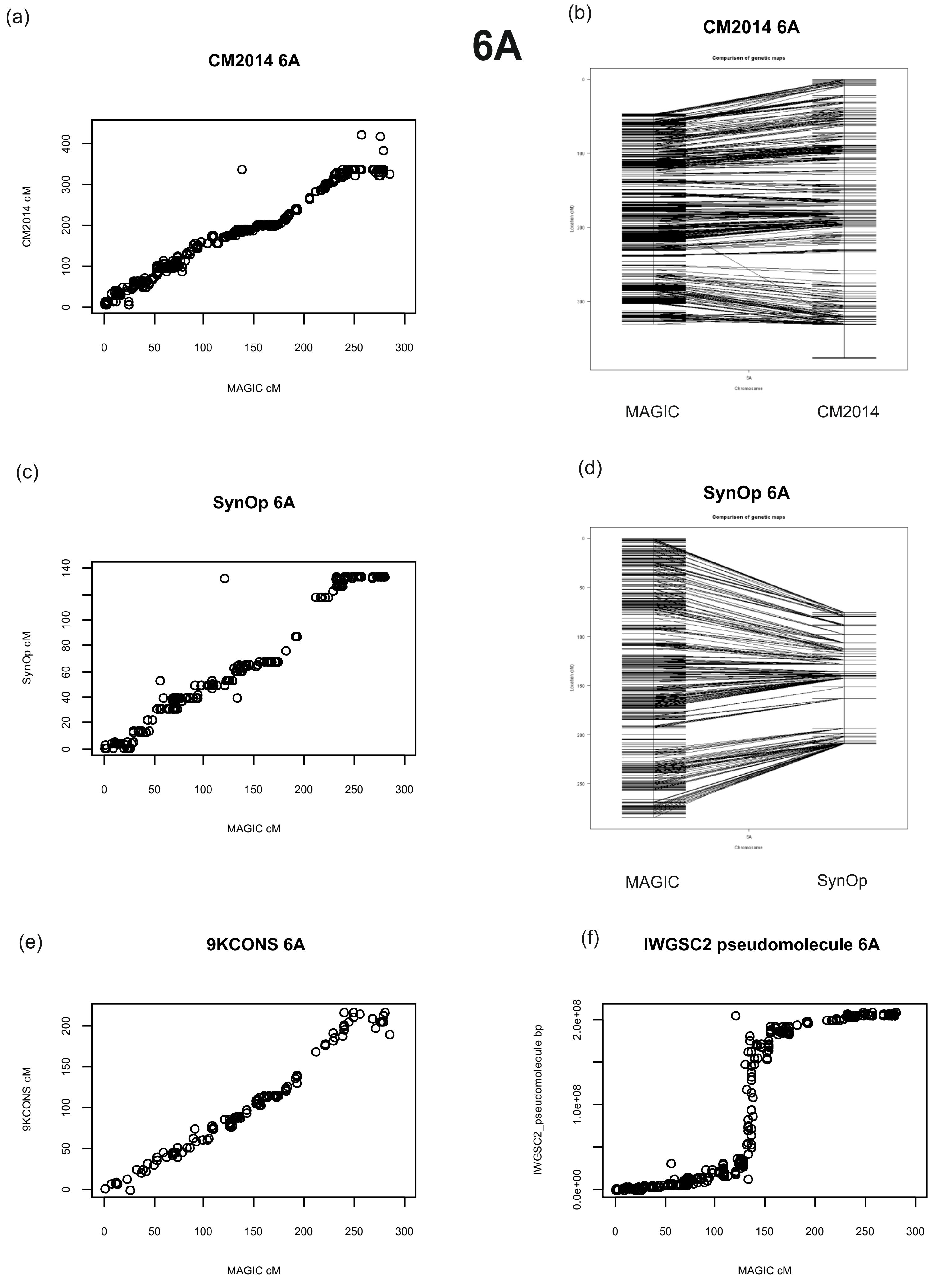
6A
(a)
CM2014 6A
CM2014 cM
MAGIC cM
(b)
CM2014 6A
Comparison of genetic maps
Location (cM)
Chromosome
MAGIC
CM2014
(c)
SynOp 6A
SynOp cM
MAGIC cM
(d)
SynOp 6A
Comparison of genetic maps
Location (cM)
Chromosome
MAGIC
SynOp
(e)
9KCONS 6A
9KCONS cM
MAGIC cM
(f)
IWGSC2 pseudomolecule 6A
IWGSC2_pseudomolecule bp
MAGIC cM

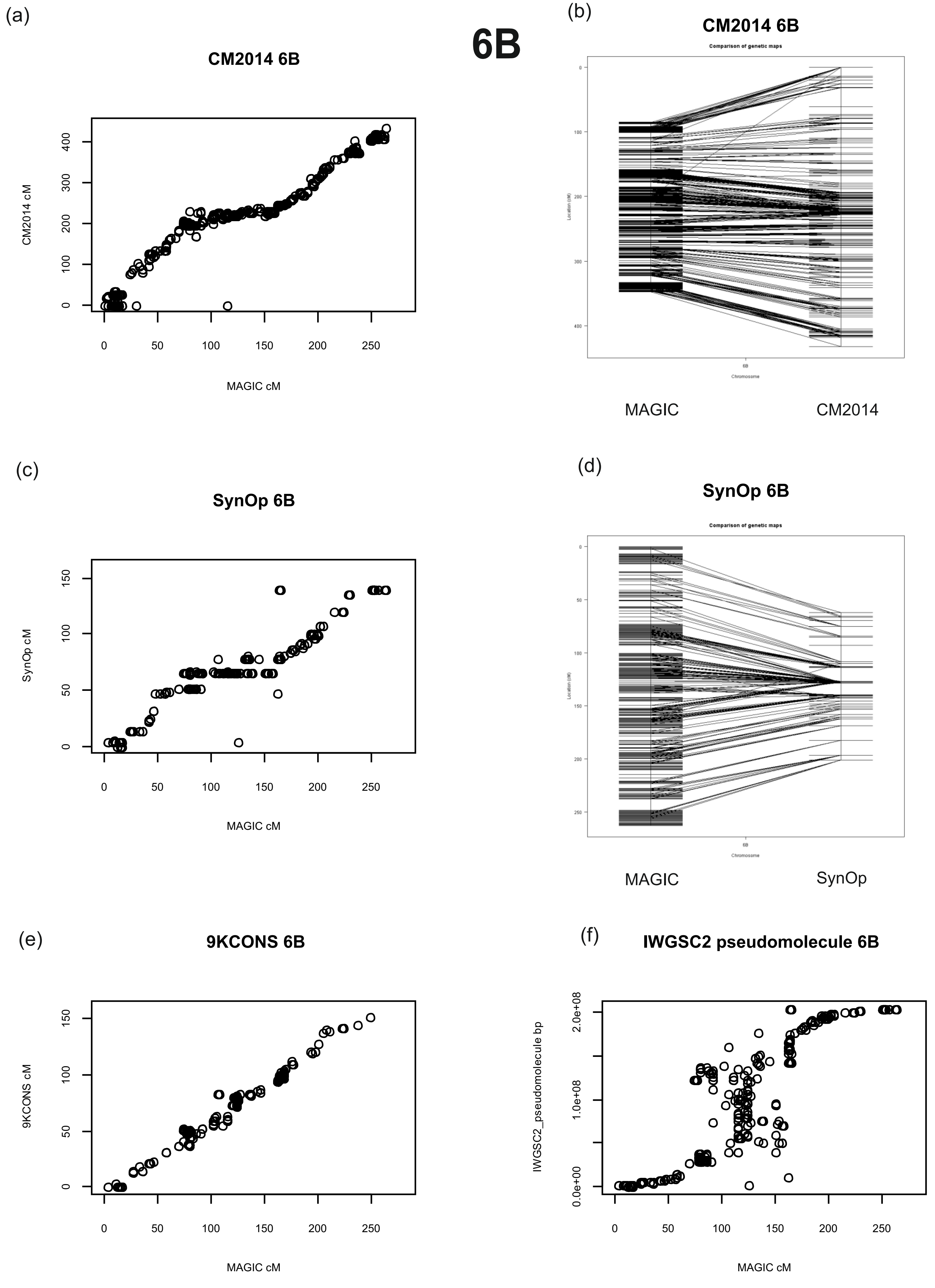

6B
(a)
CM2014 6B
CM2014 cM
MAGIC cM
(b)
CM2014 6B
Comparison of genetic maps
Location (cM)
Chromosome
MAGIC
CM2014
(c)
SynOp 6B
SynOp cM
MAGIC cM
(d)
SynOp 6B
Comparison of genetic maps
Location (cM)
Chromosome
MAGIC
SynOp
(e)
9KCONS 6B
9KCONS cM
MAGIC cM
(f)
IWGSC2 pseudomolecule 6B
IWGSC2_pseudomolecule bp
MAGIC cM

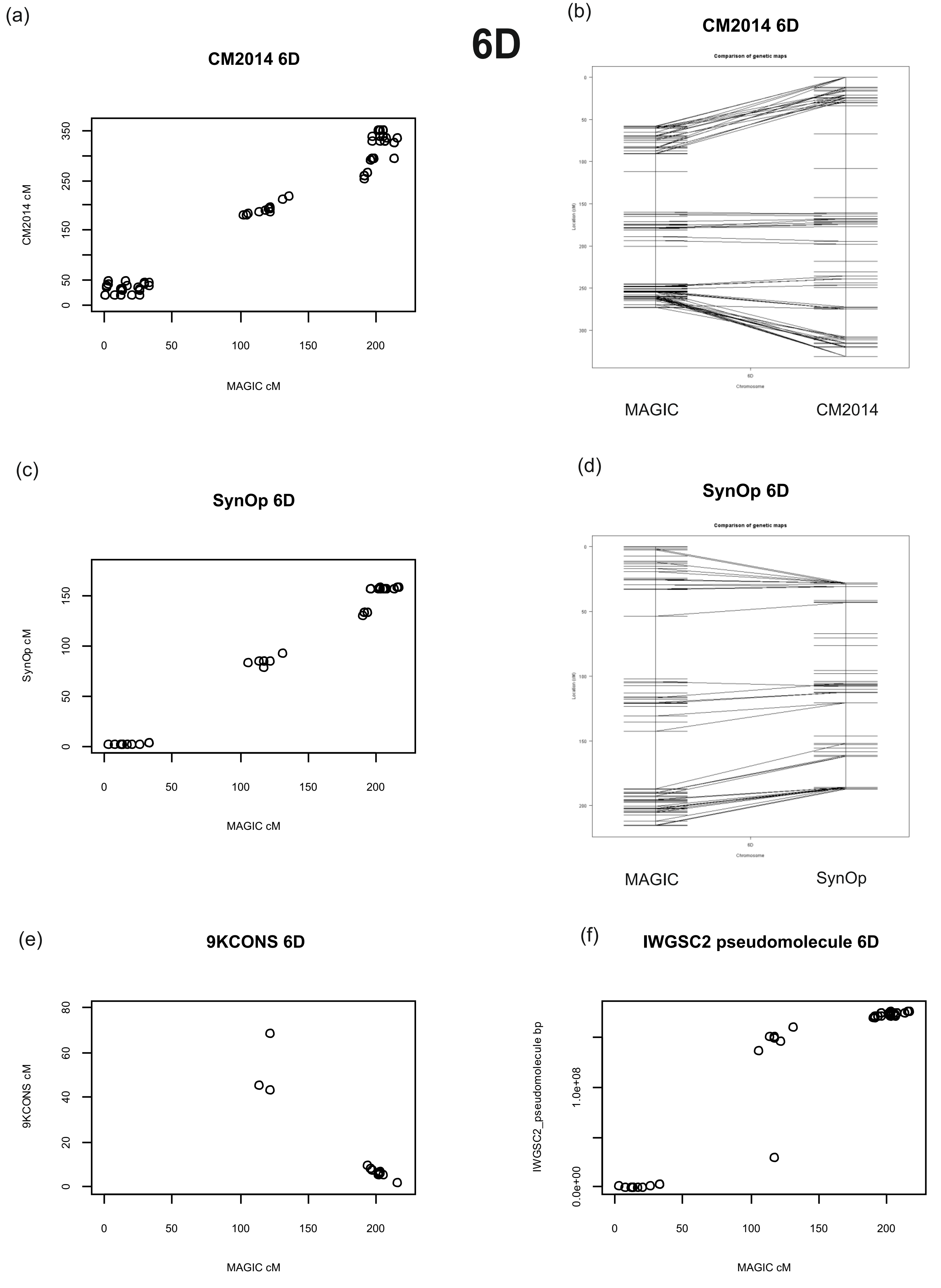
6D
(a)
CM2014 6D
CM2014 cM
MAGIC cM
0
50
150
250
350
0
50
100
150
200
(b)
CM2014 6D
Comparison of genetic maps
Location (cM)
Chromosome
6D
MAGIC
CM2014
(c)
SynOp 6D
SynOp cM
MAGIC cM
0
50
100
150
200
(d)
SynOp 6D
Comparison of genetic maps
Location (cM)
Chromosome
6D
MAGIC
SynOp
(e)
9KCONS 6D
9KCONS cM
MAGIC cM
0
20
40
60
80
100
150
200
(f)
IWGSC2 pseudomolecule 6D
IWGSC2_pseudomolecule bp
0.0e+00
1.0e+08
MAGIC cM
0
50
100
150
200

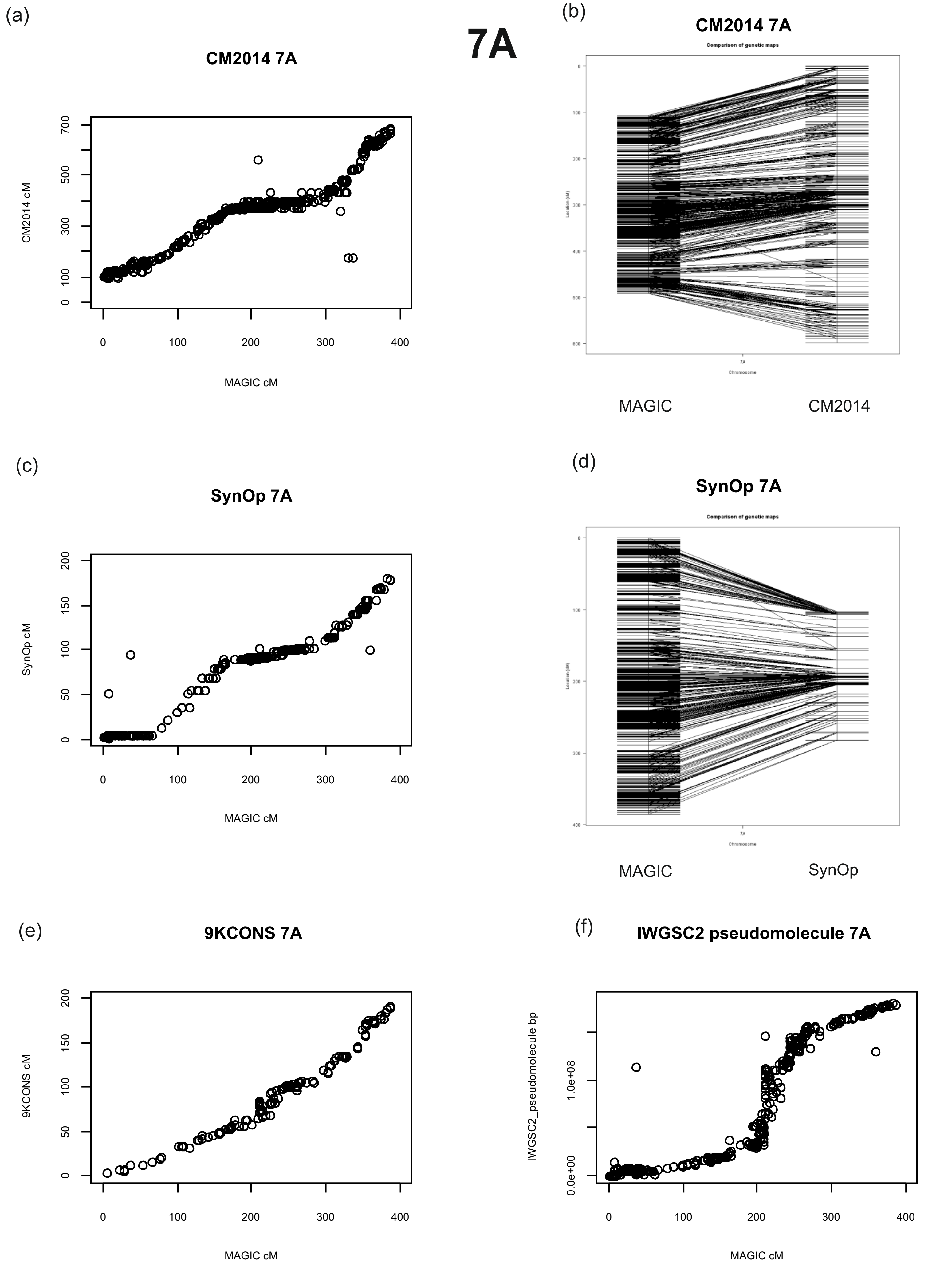
7A
(a)
CM2014 7A
CM2014 cM
MAGIC cM
(b)
CM2014 7A
Comparison of genetic maps
Location (cM)
Chromosome
MAGIC
CM2014
(c)
SynOp 7A
SynOp cM
MAGIC cM
(d)
SynOp 7A
Comparison of genetic maps
Location (cM)
Chromosome
MAGIC
SynOp
(e)
9KCONS 7A
9KCONS cM
MAGIC cM
(f)
IWGSC2 pseudomolecule 7A
IWGSC2_pseudomolecule bp
MAGIC cM

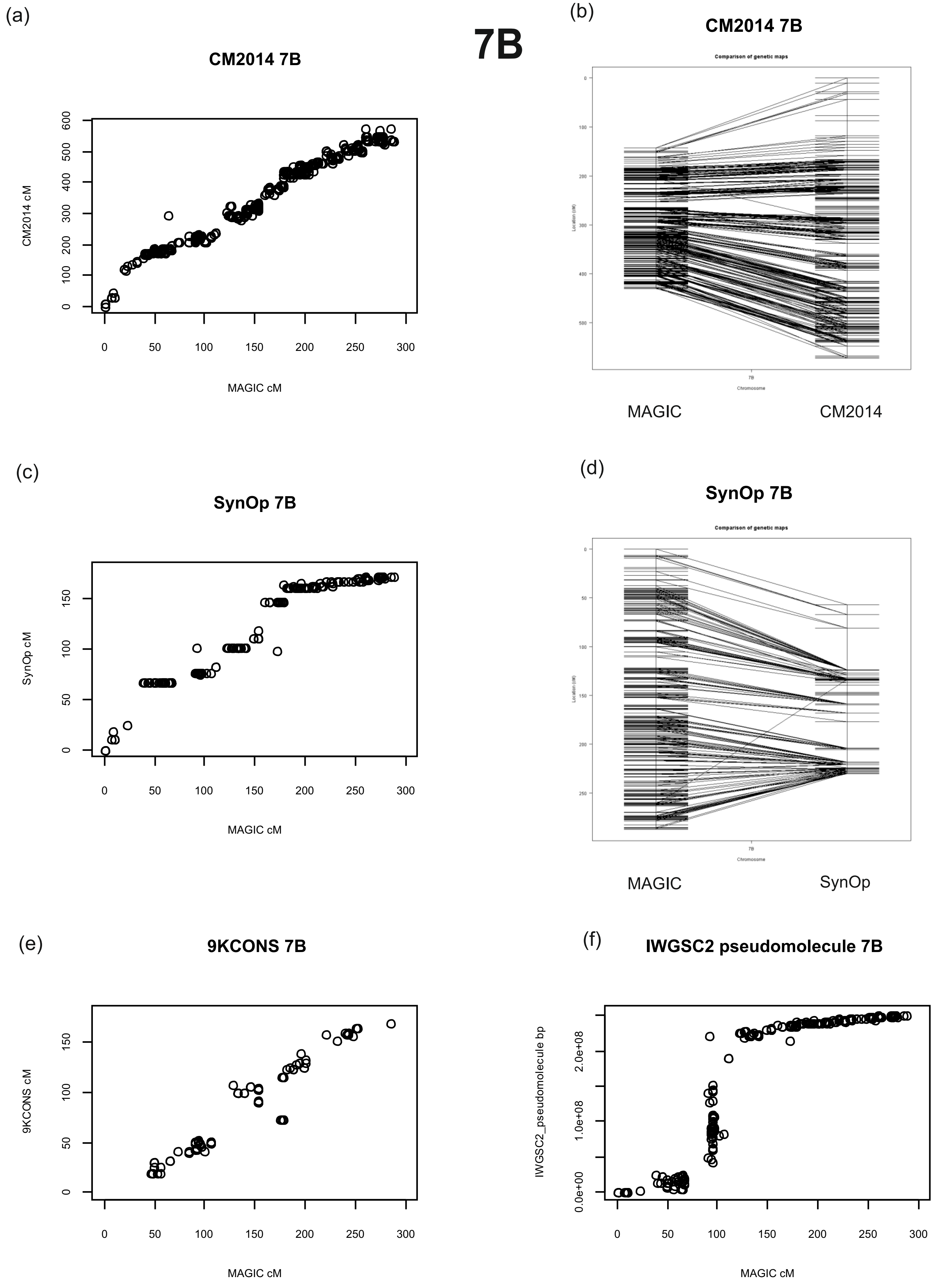

7B
(a)
CM2014 7B
CM2014 cM
MAGIC cM
(b)
CM2014 7B
Comparison of genetic maps
Location (cM)
7B
Chromosome
MAGIC
CM2014
(c)
SynOp 7B
SynOp cM
MAGIC cM
(d)
SynOp 7B
Comparison of genetic maps
Location (cM)
7B
Chromosome
MAGIC
SynOp
(e)
9KCONS 7B
9KCONS cM
MAGIC cM
(f)
IWGSC2 pseudomolecule 7B
IWGSC2_pseudomolecule bp
MAGIC cM

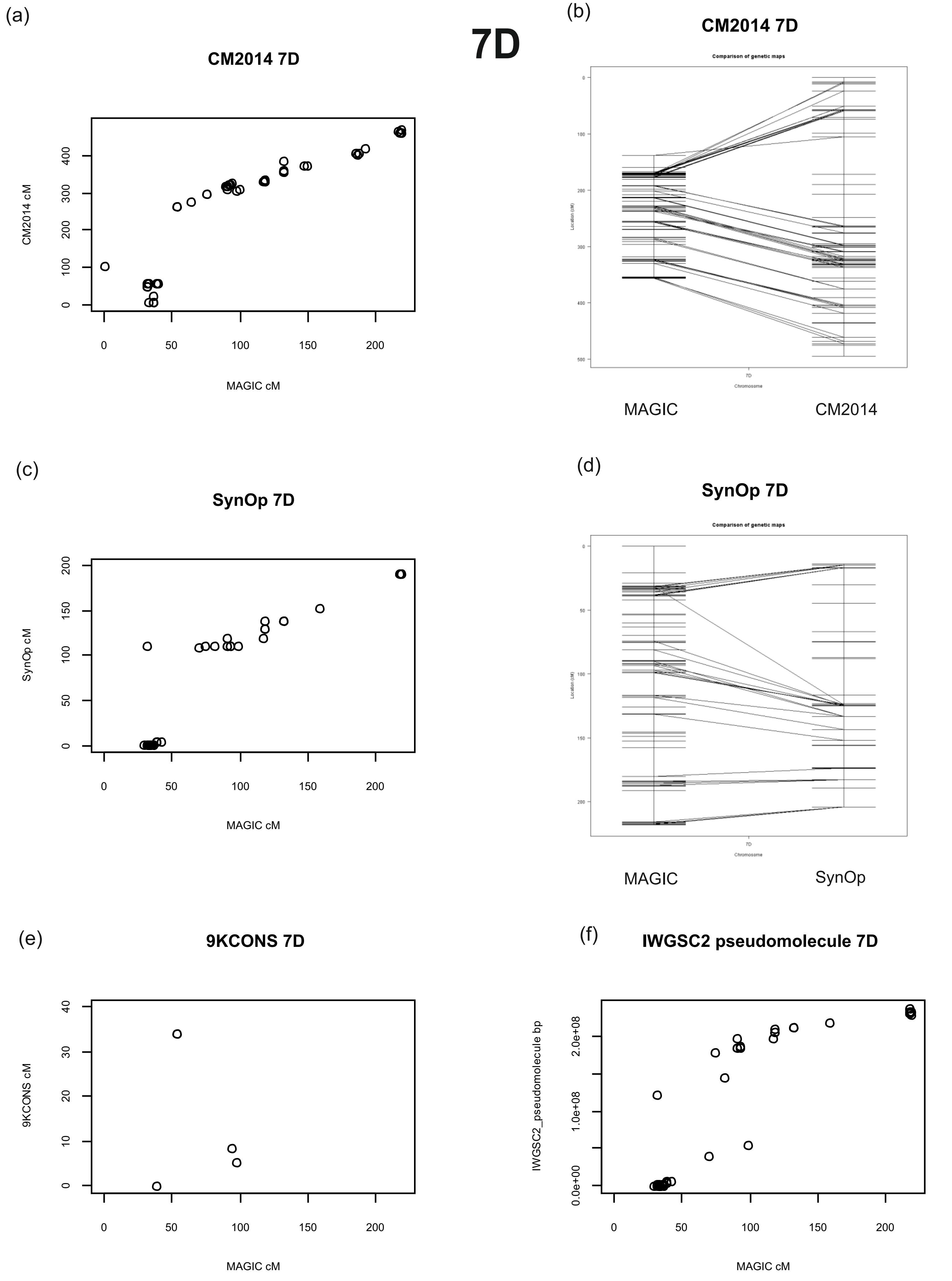

7D
(a)
CM2014 7D
CM2014 cM
MAGIC cM
0
100
200
300
400
0
50
100
150
200
(b)
CM2014 7D
Comparison of genetic maps
Location (cM)
Chromosome
7D
0
100
200
300
400
500
MAGIC
CM2014
(c)
SynOp 7D
SynOp cM
MAGIC cM
0
50
100
150
200
(d)
SynOp 7D
Comparison of genetic maps
Location (cM)
Chromosome
7D
MAGIC
SynOp
(e)
9KCONS 7D
9KCONS cM
MAGIC cM
0
10
20
30
40
(f)
IWGSC2 pseudomolecule 7D
IWGSC2_pseudomolecule bp
MAGIC cM
0.0e+00
1.0e+08
2.0e+08
