# Supplementary material for: A highly recombined, high‐density, eight‐founder wheat MAGIC map reveals extensive segregation distortion and genomic locations of introgression segments
Source: Plant Biotechnol J. 2016 Jan 23;14(6):1406–17. doi: 10.1111/pbi.12504 (PMC4985697; doi:10.1111/pbi.12504)
Supplement: Supplementary file 3 — Figure S3 LD decay (measured as R 2 and D') compared across all chromosomes for NIAB2015 and CM2014. [file PBI-14-1406-s003.pdf]

1A

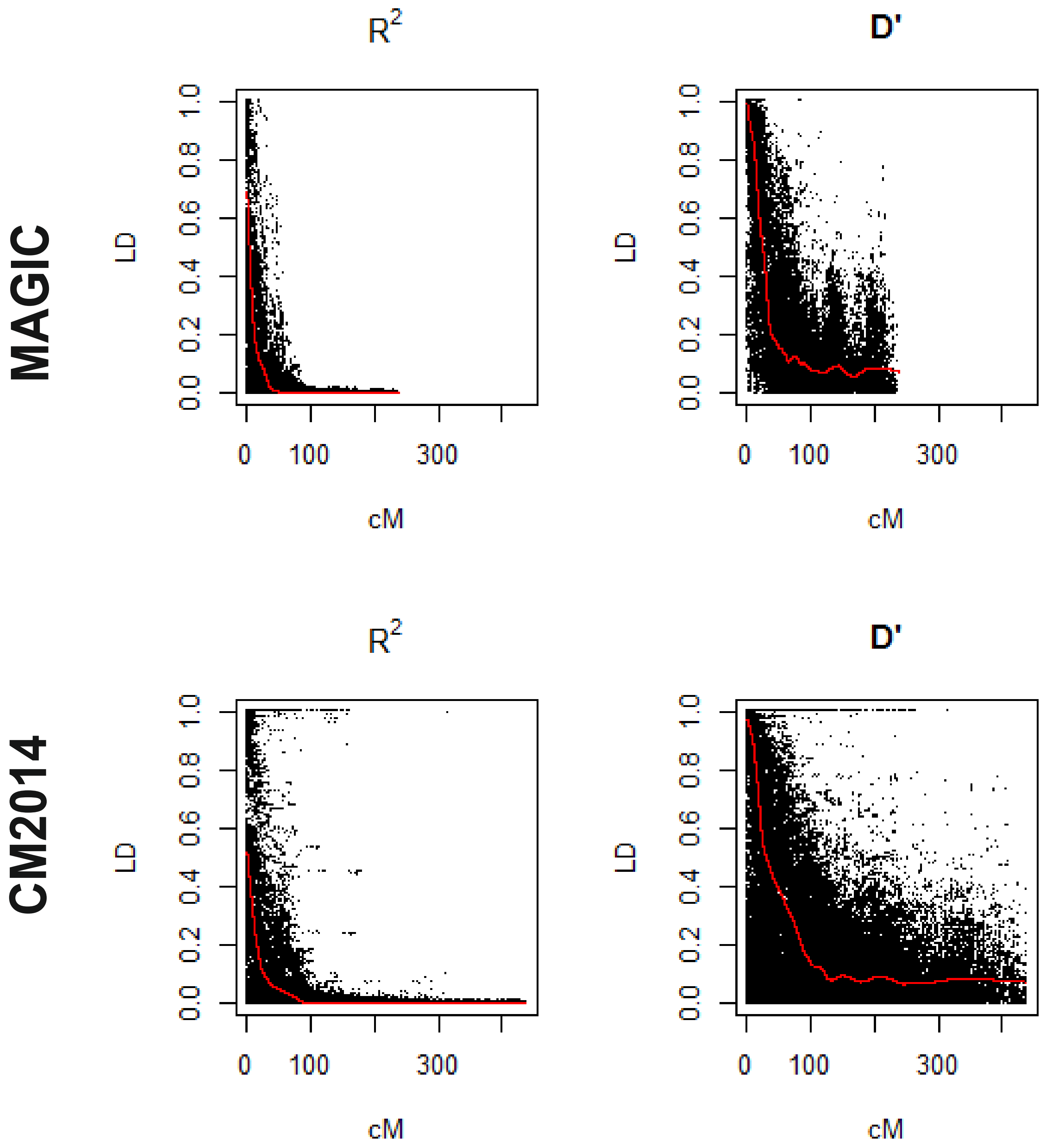

1B

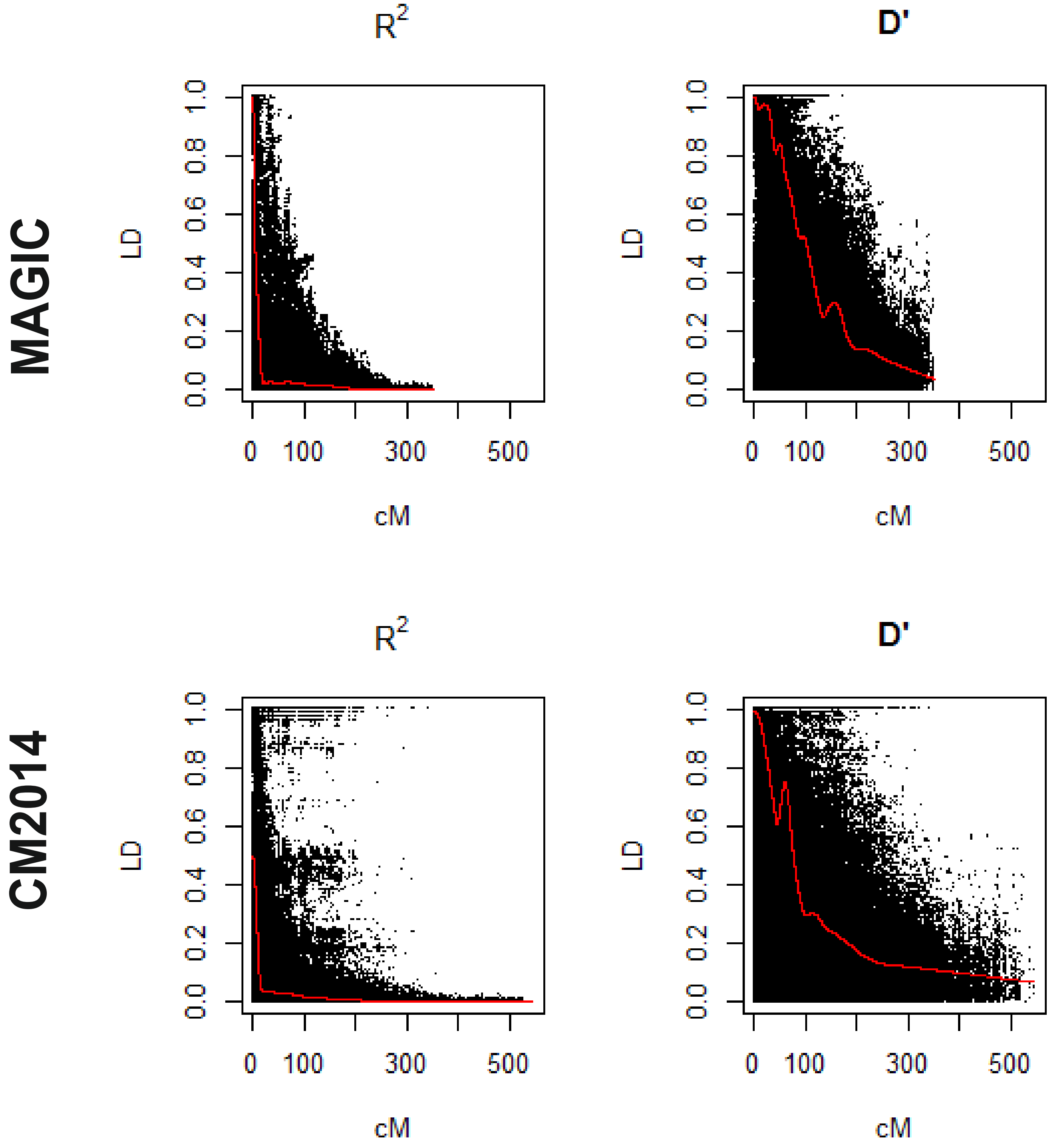

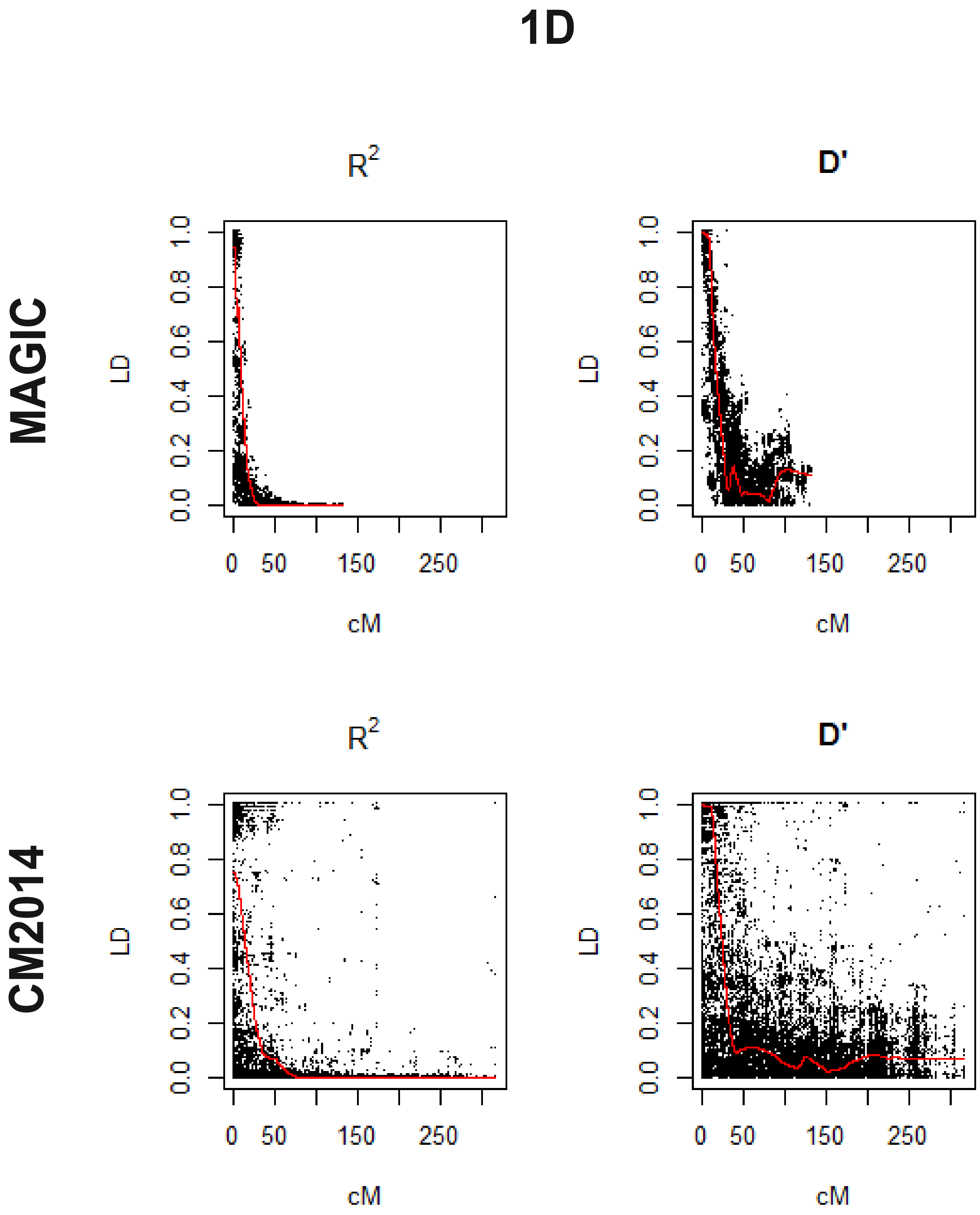
1D
MAGIC
CM2014
R2
D'
LD
cM
0.0
0.2
0.4
0.6
0.8
1.0
0
50
150
250

2A

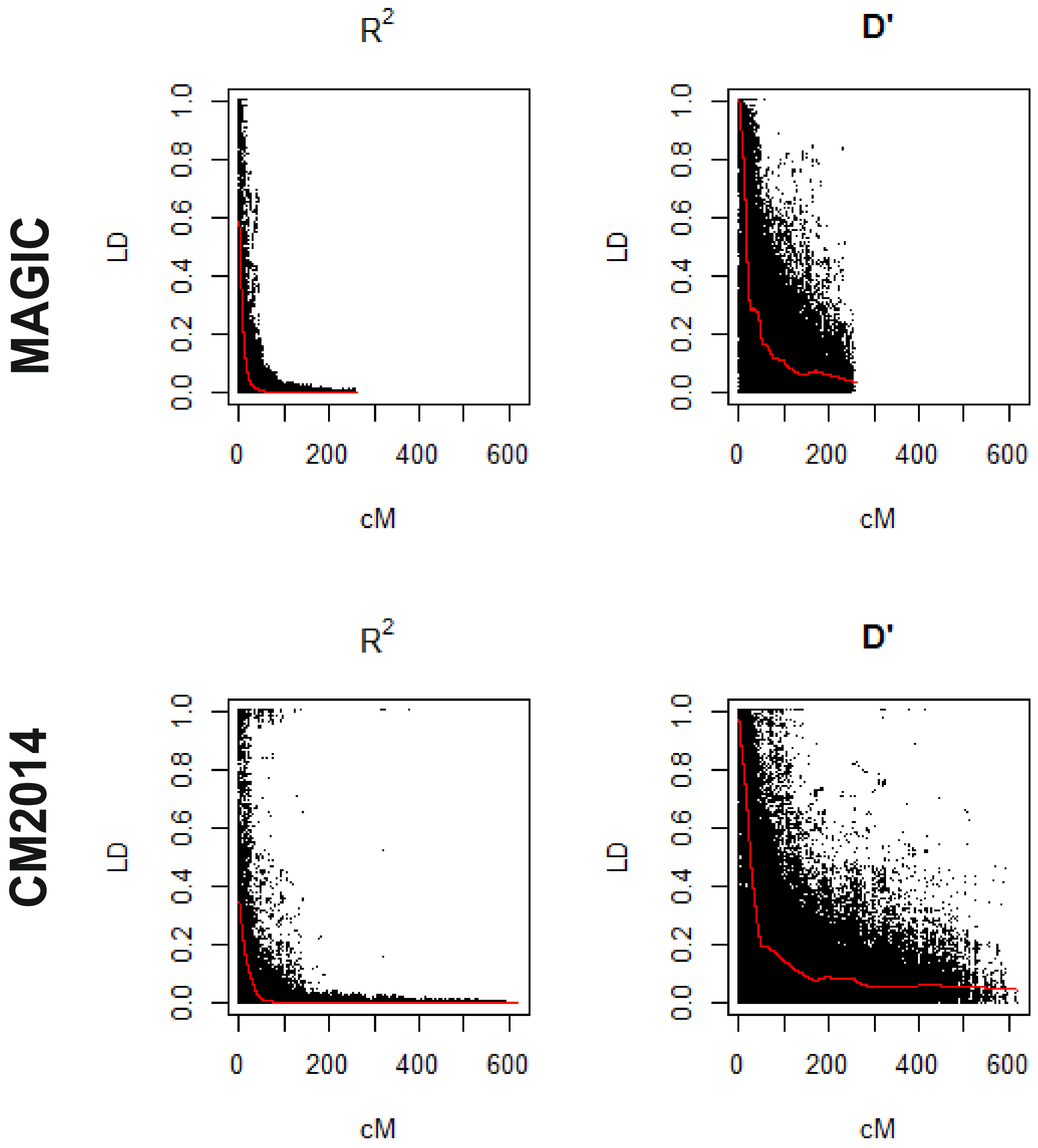

2B

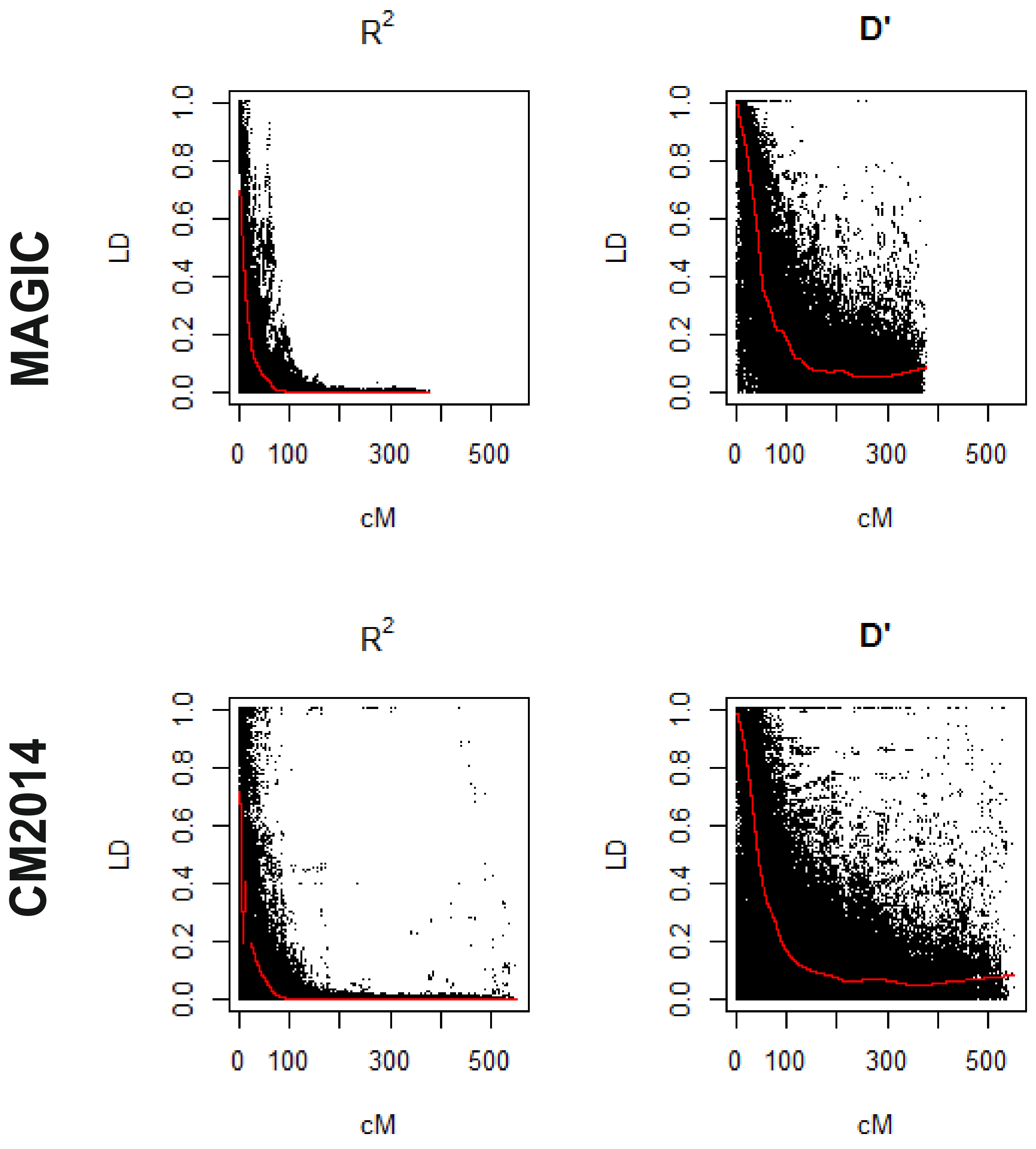

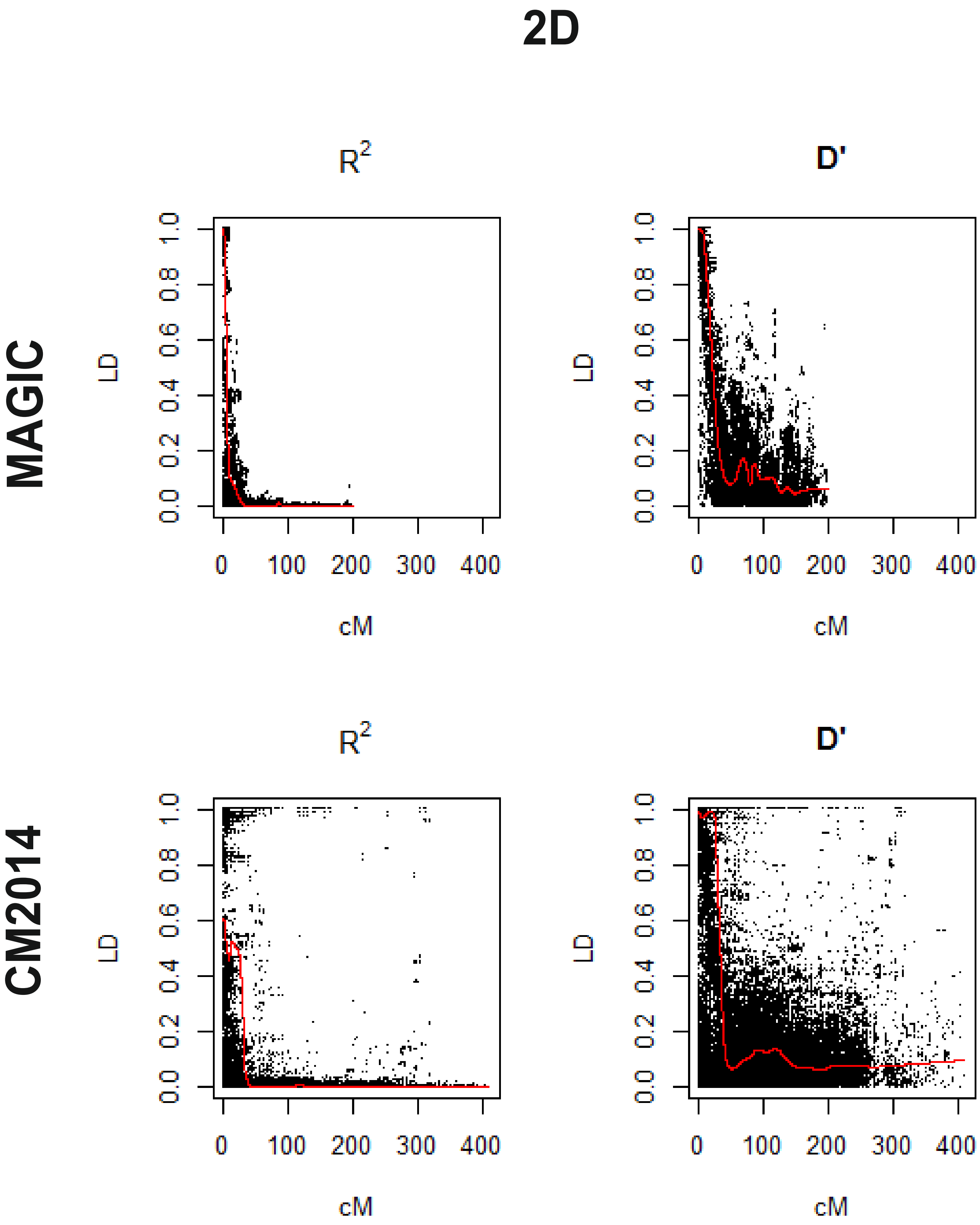
2D
R2
D'
MAGIC
CM2014
LD
cM
0.0
0.2
0.4
0.6
0.8
1.0
0
100
200
300
400

3A

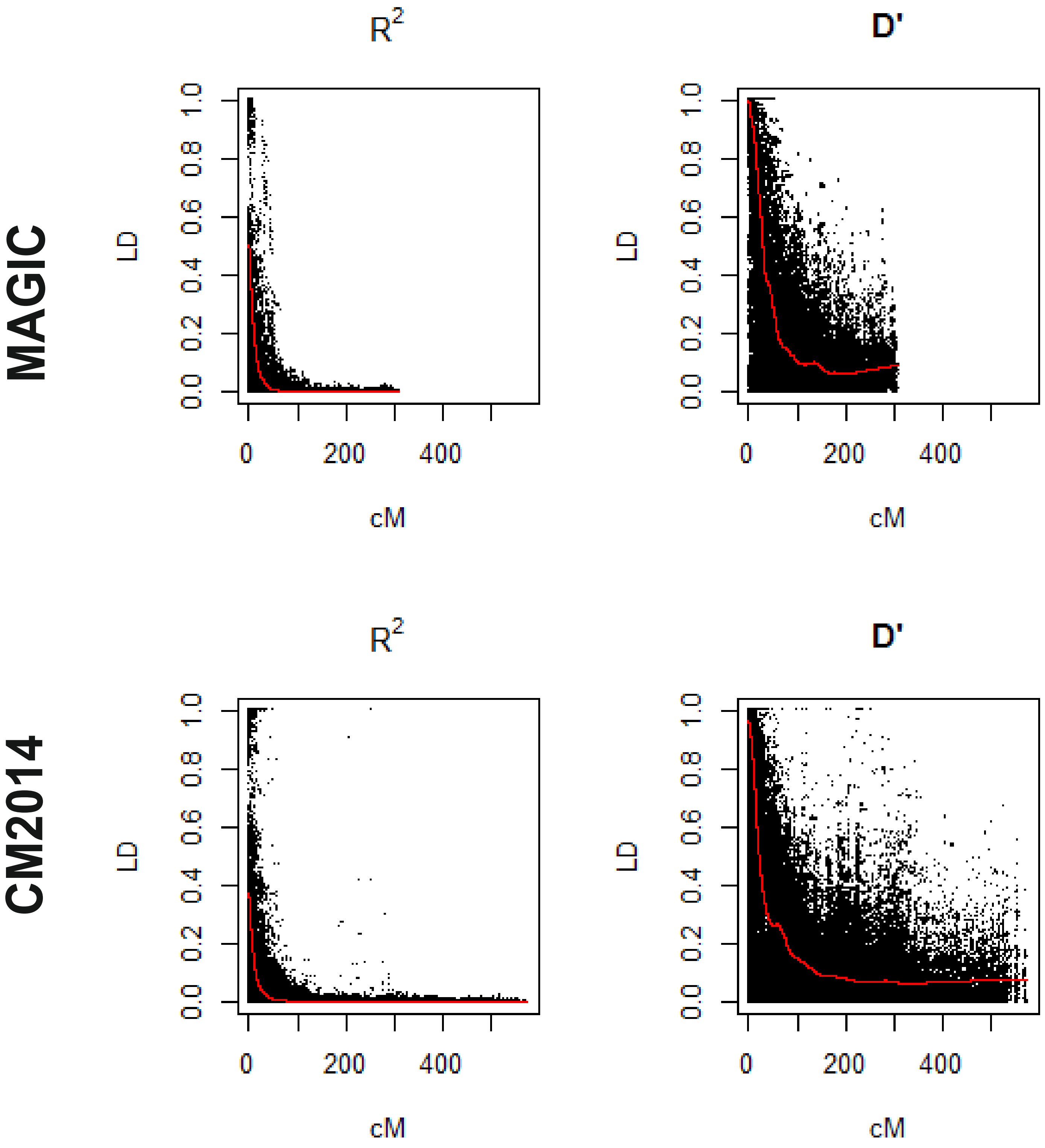

3B

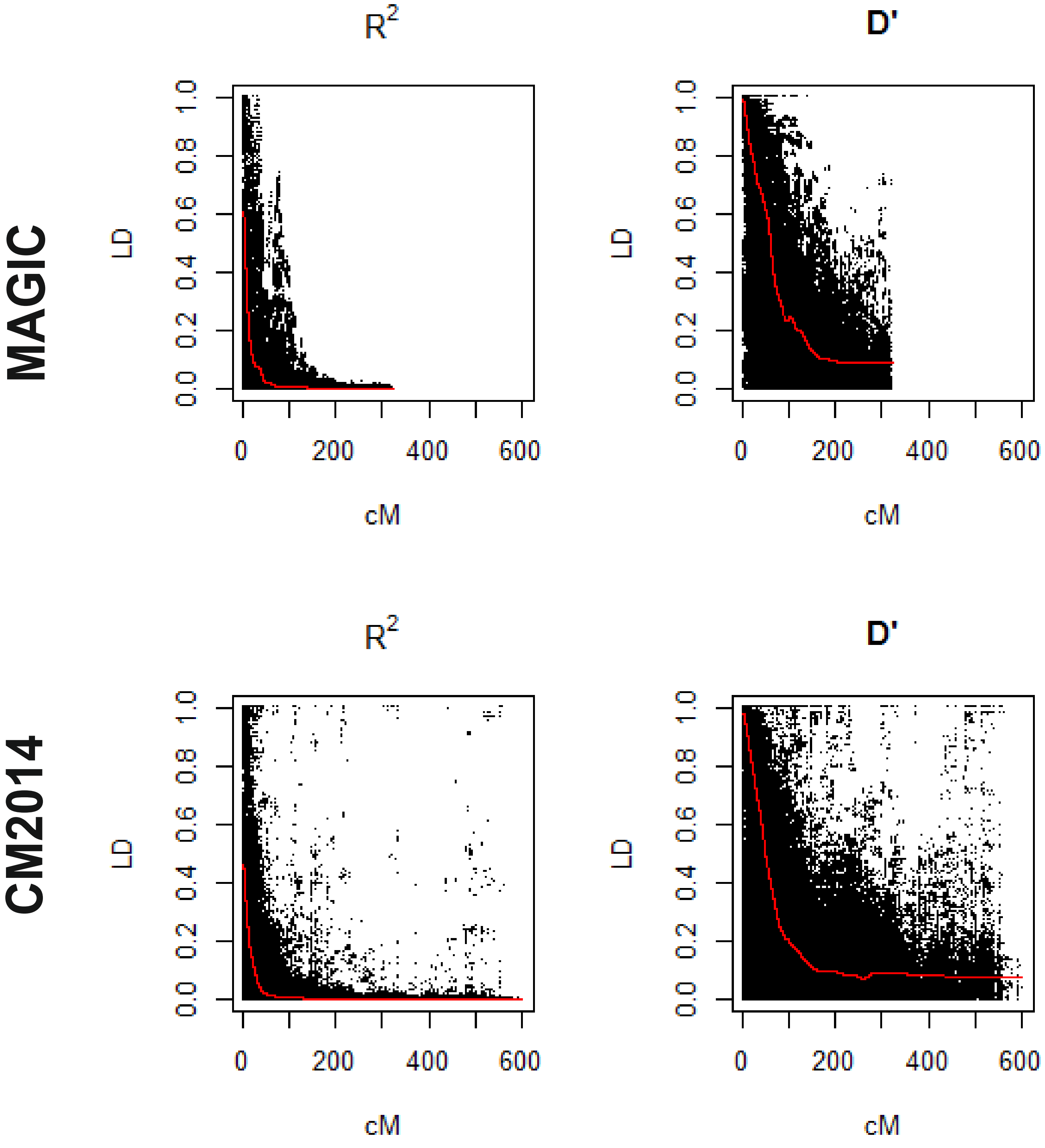

3D

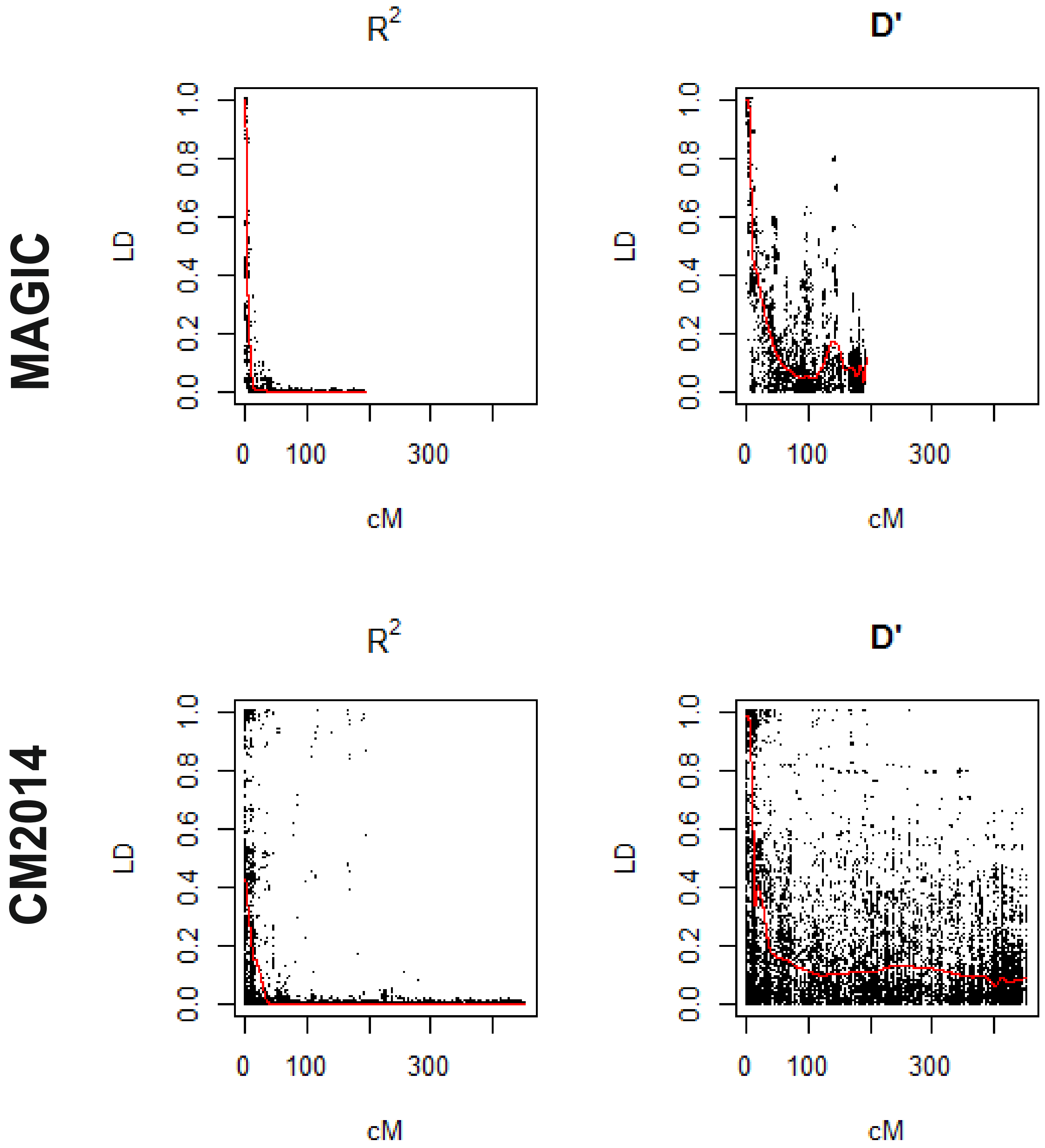

4A

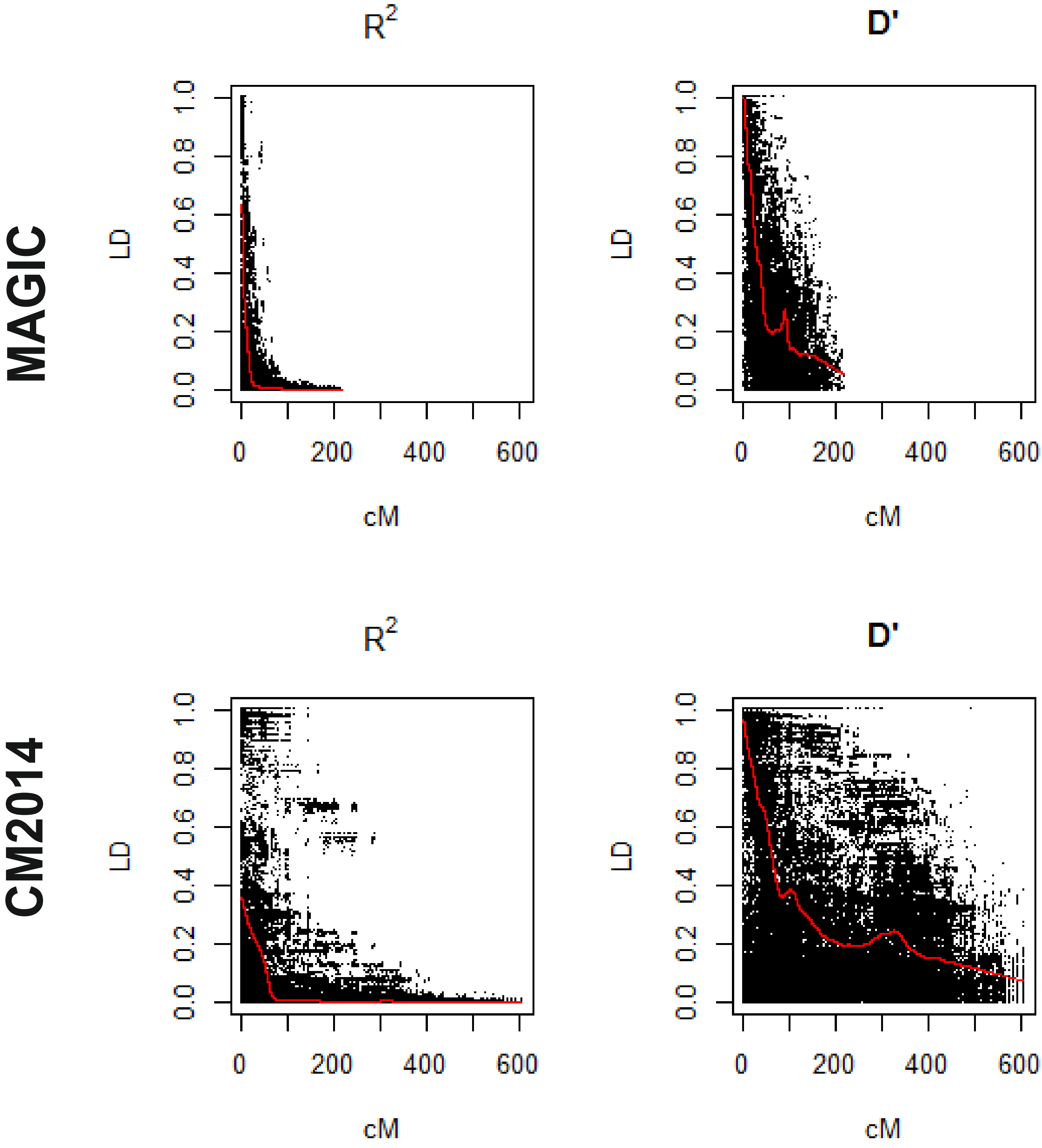

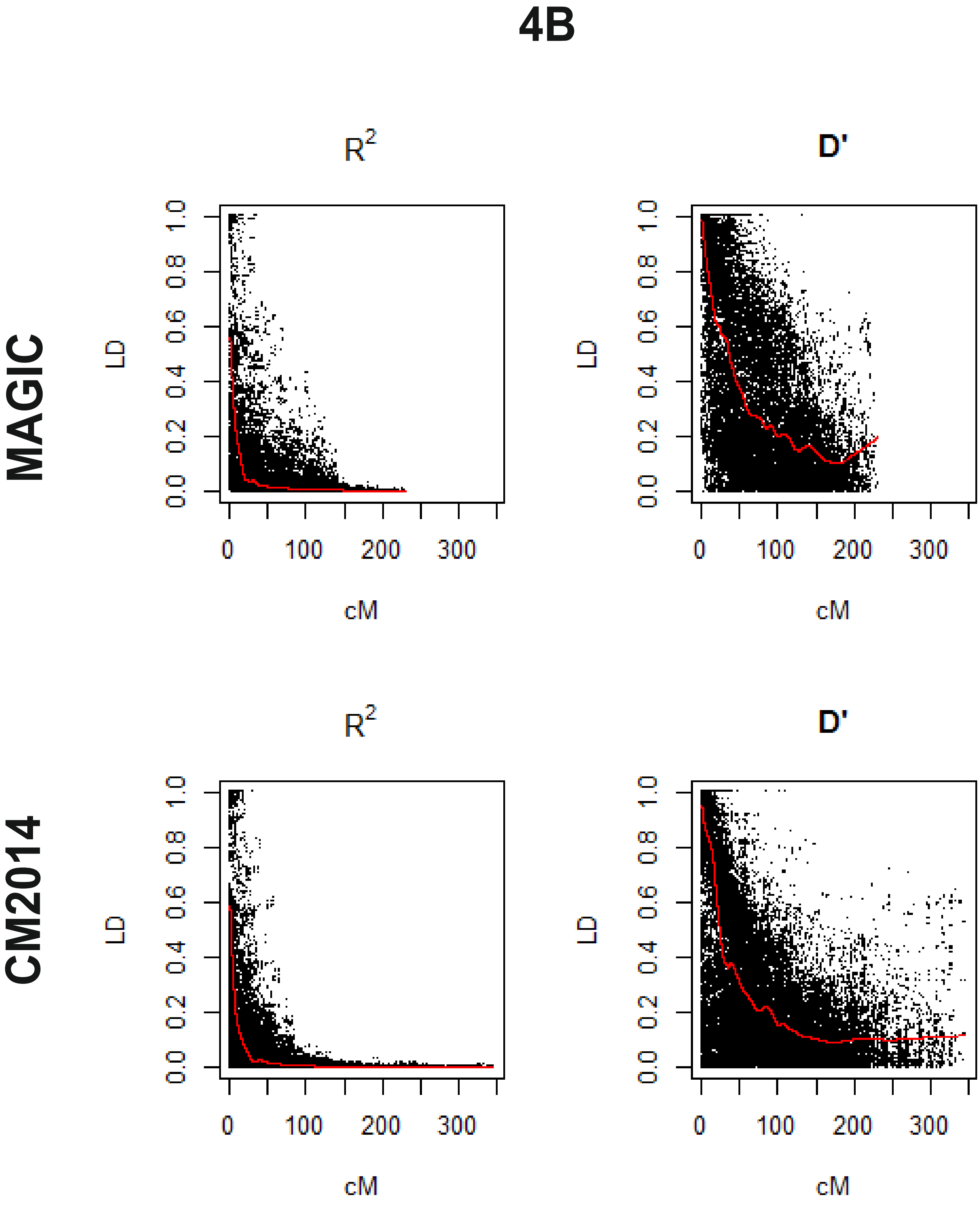

4B
MAGIC
CM2014
R^2
D'
LD
cM
0.0
0.2
0.4
0.6
0.8
1.0
0
100
200
300

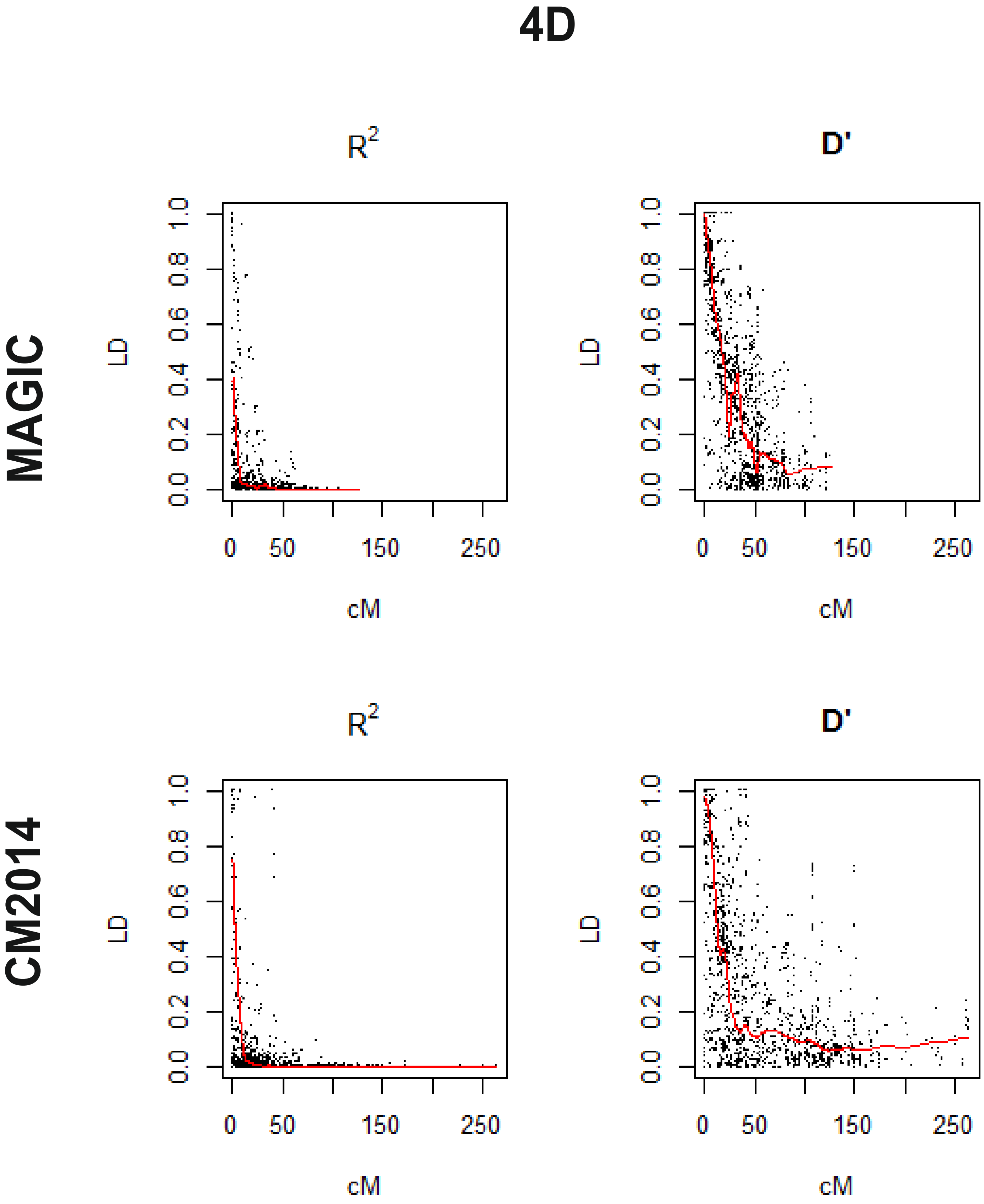
4D
MAGIC
CM2014
R2
D'
LD
cM
0.0
0.2
0.4
0.6
0.8
1.0
0
50
150
250

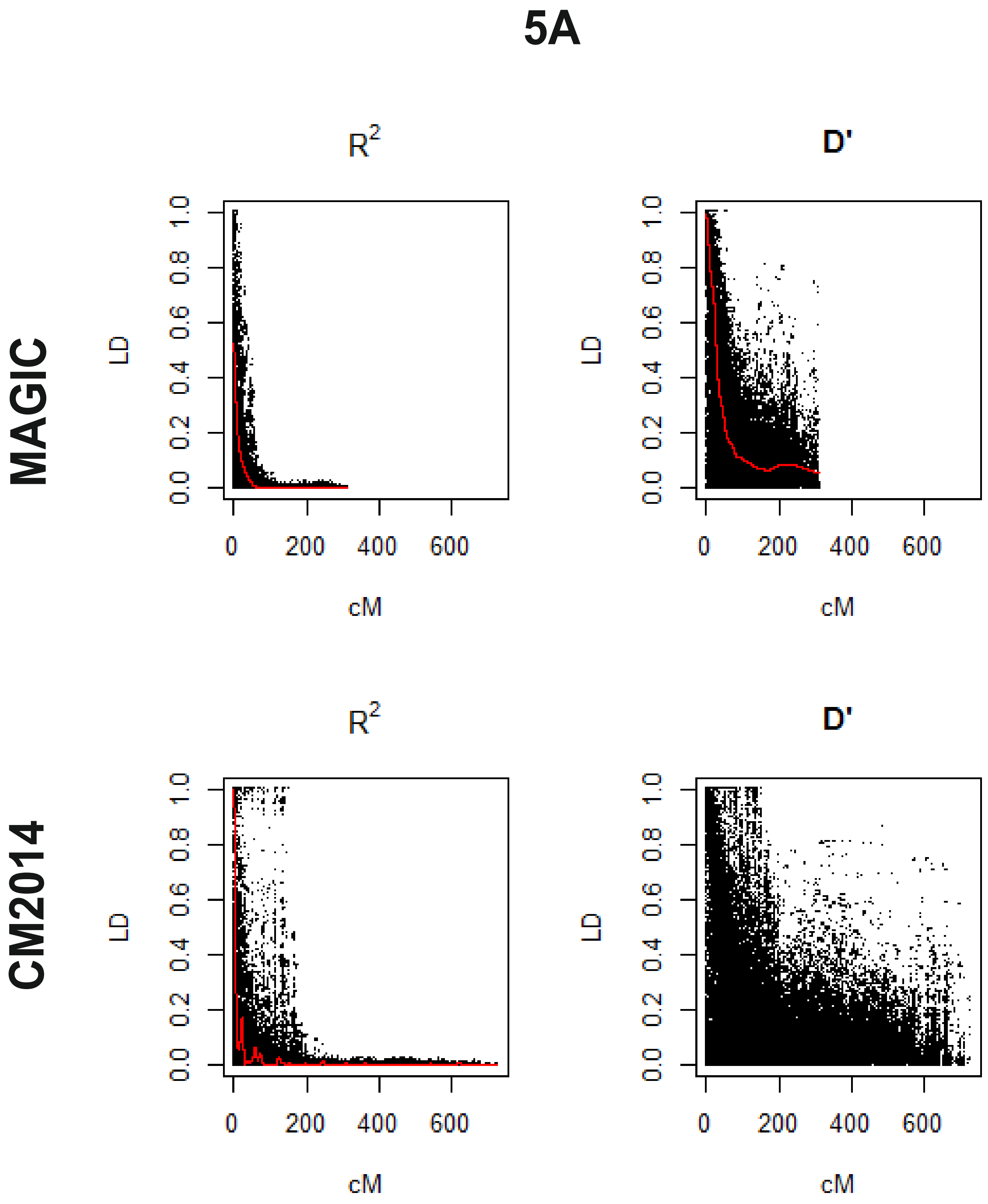
5A
MAGIC
CM2014
R2
D'
LD
cM
0.0 0.2 0.4 0.6 0.8 1.0
0 200 400 600

5B

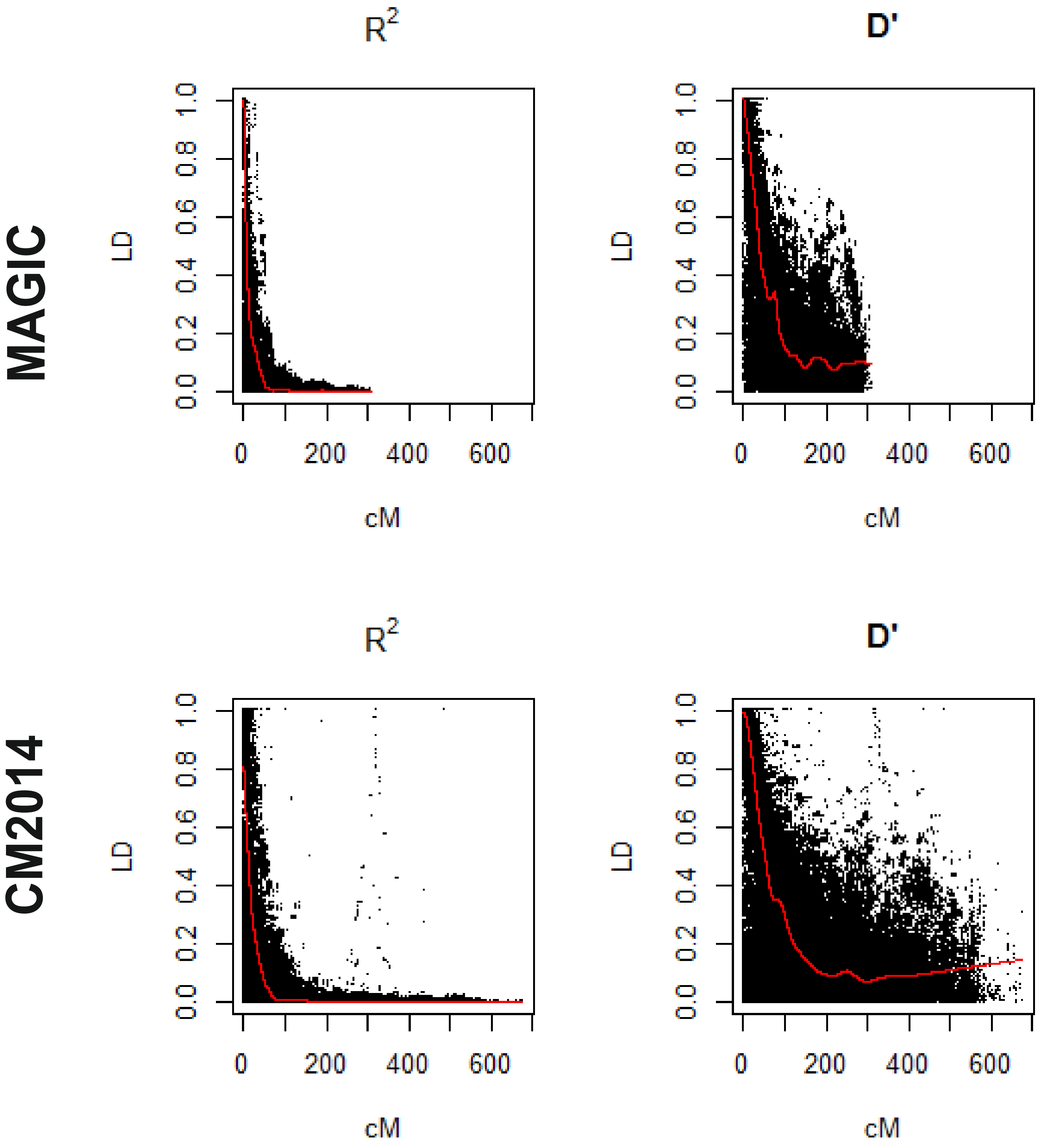

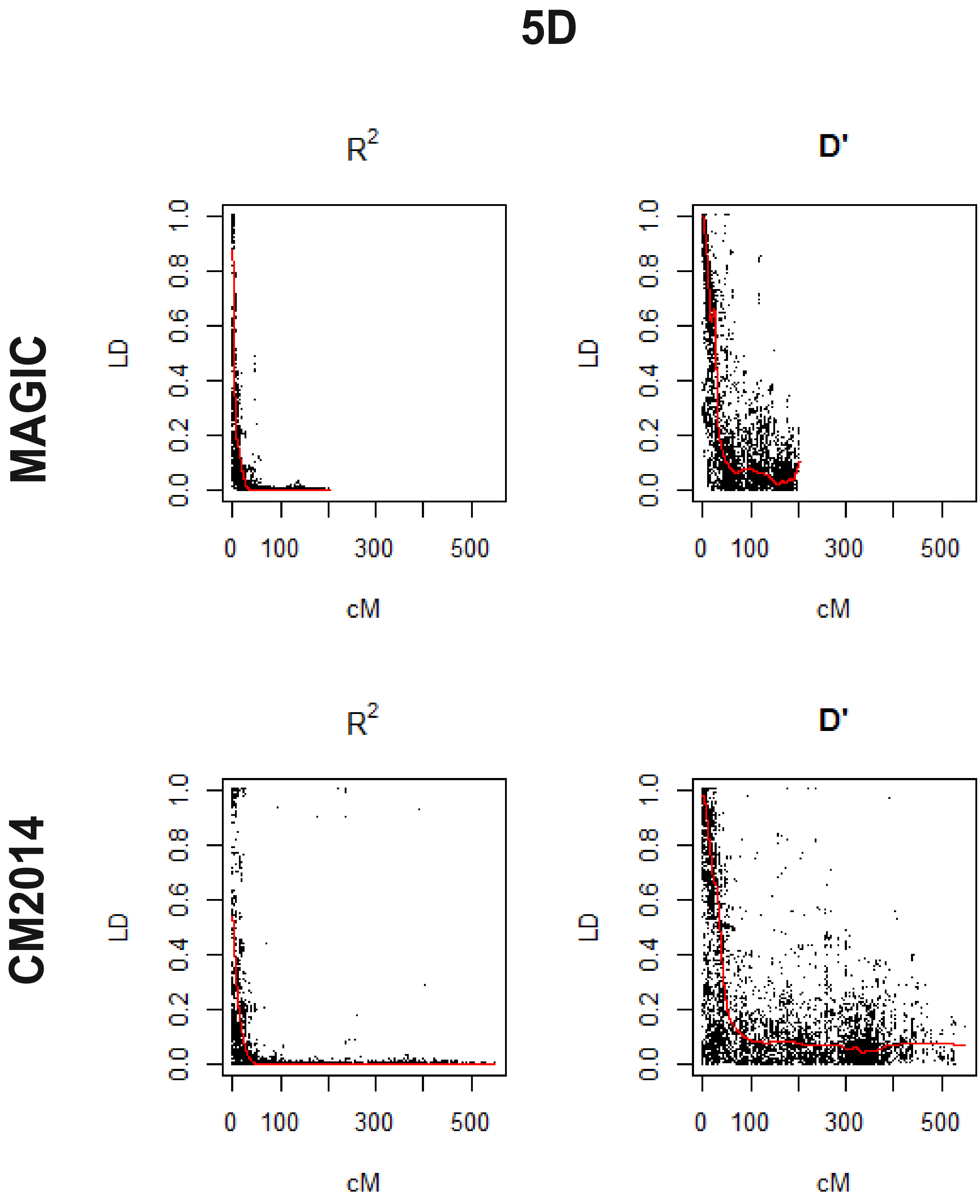
5D
MAGIC
CM2014
R2
D'
LD
cM
0.0
0.2
0.4
0.6
0.8
1.0
0
100
300
500

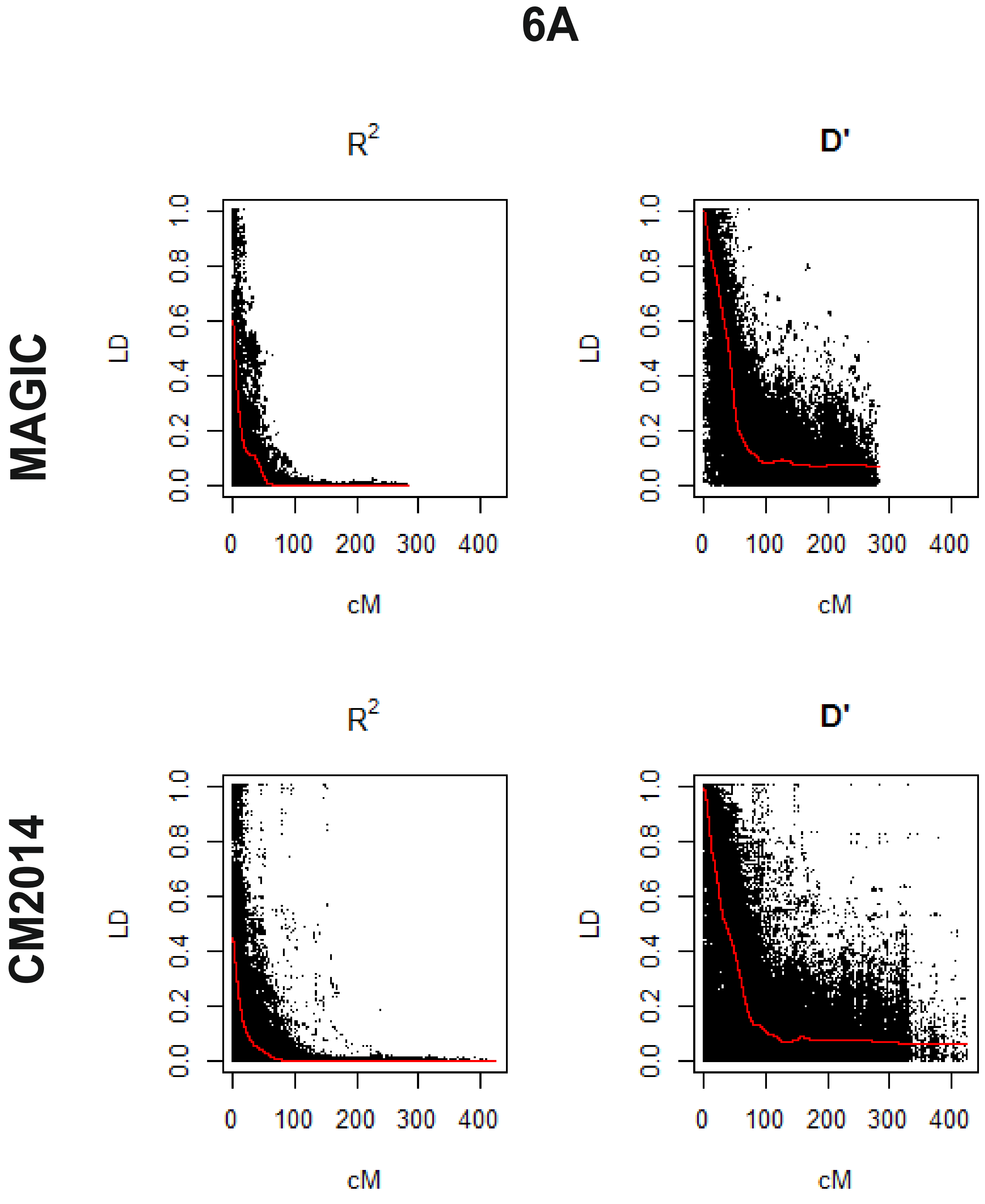
6A
MAGIC
CM2014
R^2
D'
R^2
D'
LD
LD
LD
LD
0.0
0.2
0.4
0.6
0.8
1.0
0
100
200
300
400
cM
cM
cM
cM

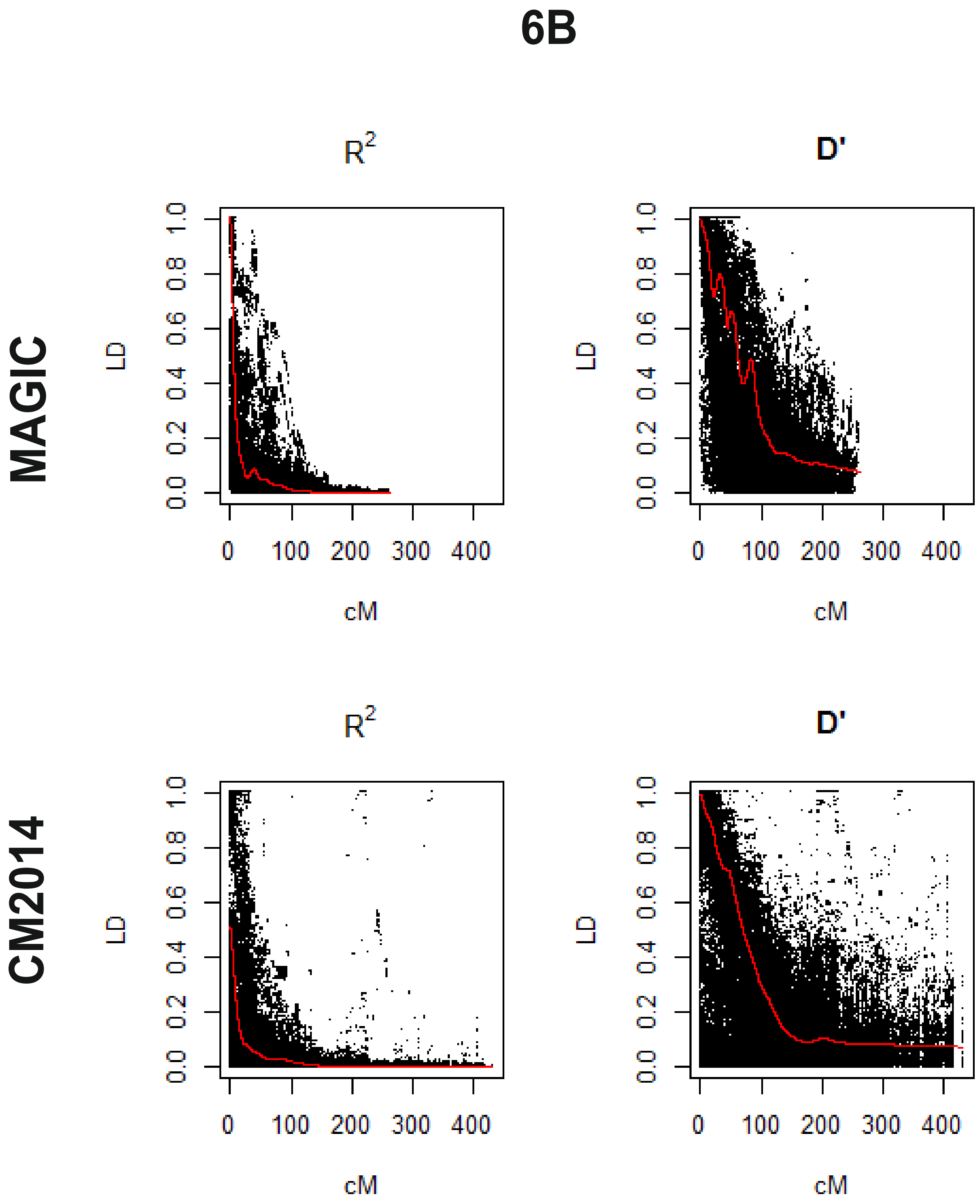
6B
MAGIC
CM2014
R2
D'
LD
cM
0.0
0.2
0.4
0.6
0.8
1.0
0
100
200
300
400

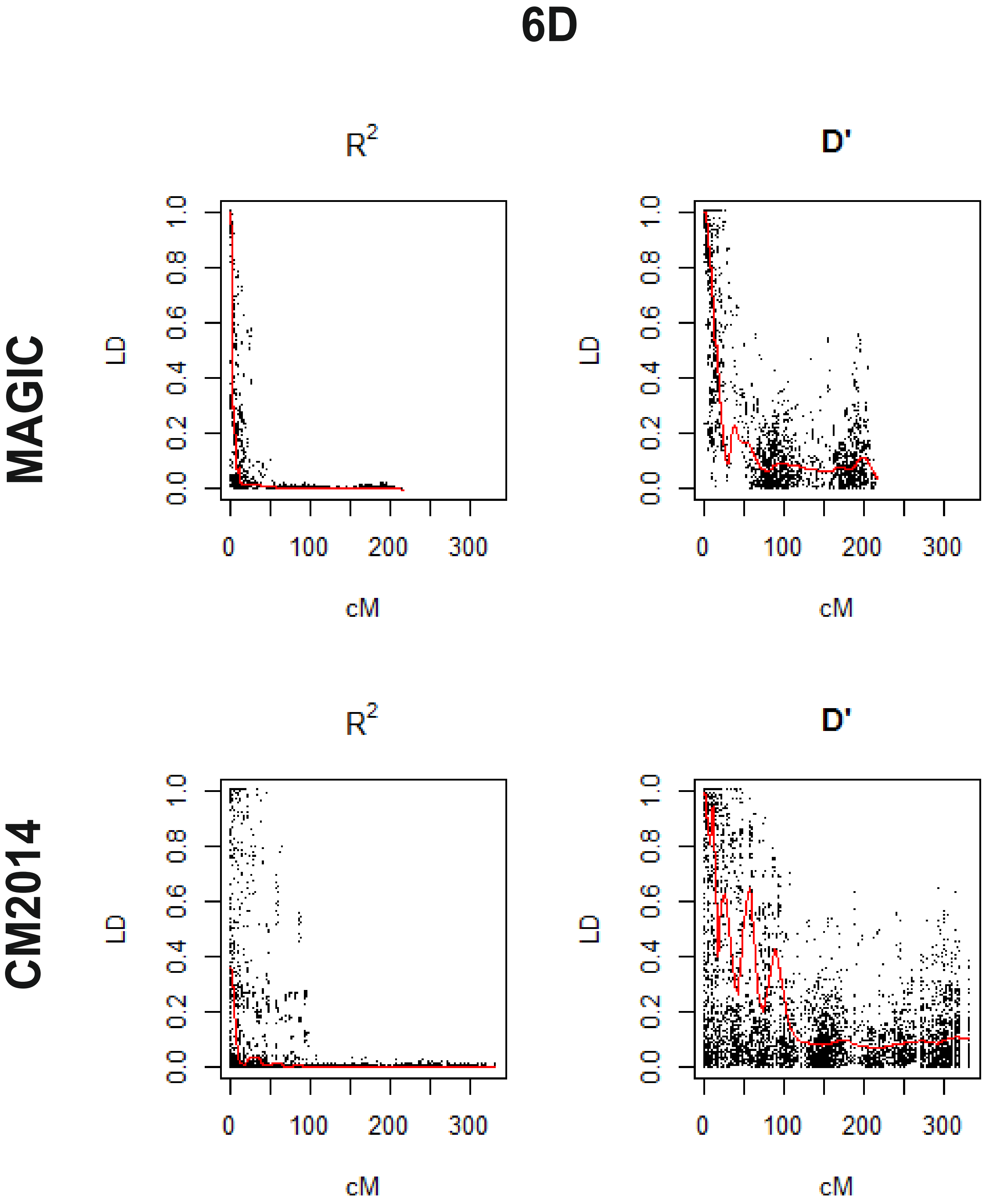

6D
MAGIC
CM2014
R^2
D'
LD
cM
0.0
0.2
0.4
0.6
0.8
1.0
0
100
200
300

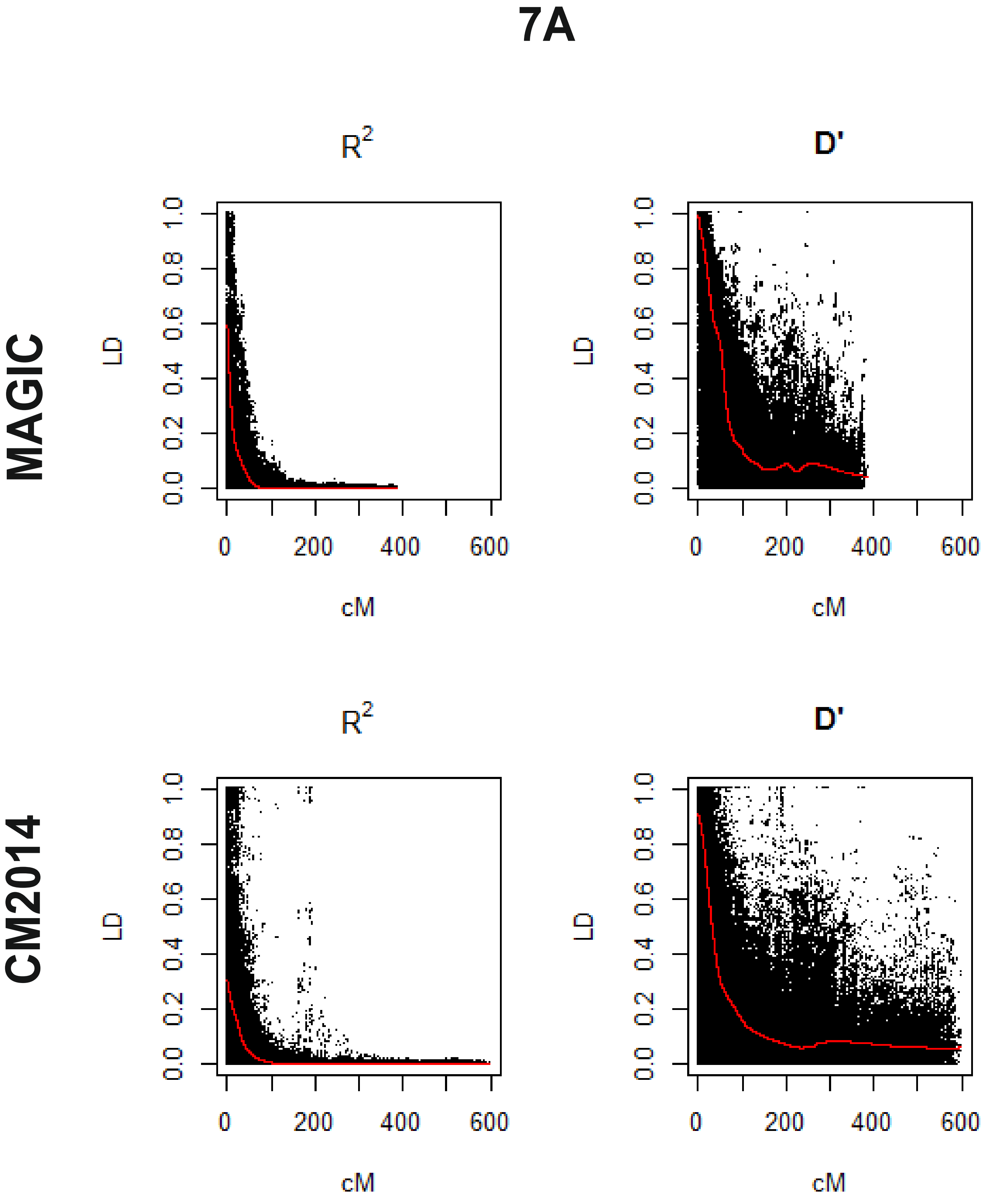

7A
MAGIC
CM2014
R2
D'
LD
cM
0.0
0.2
0.4
0.6
0.8
1.0
0
200
400
600

7B

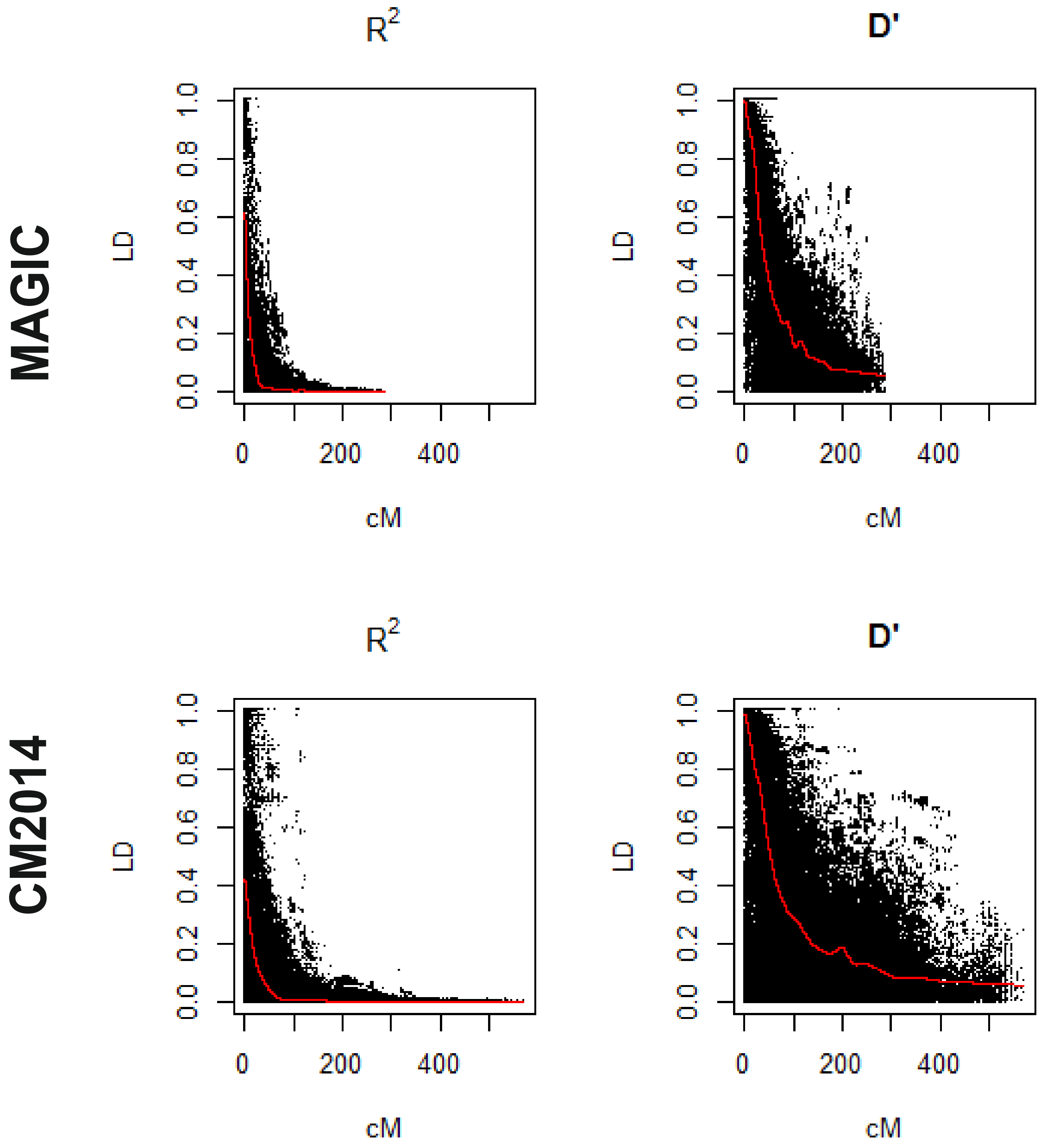

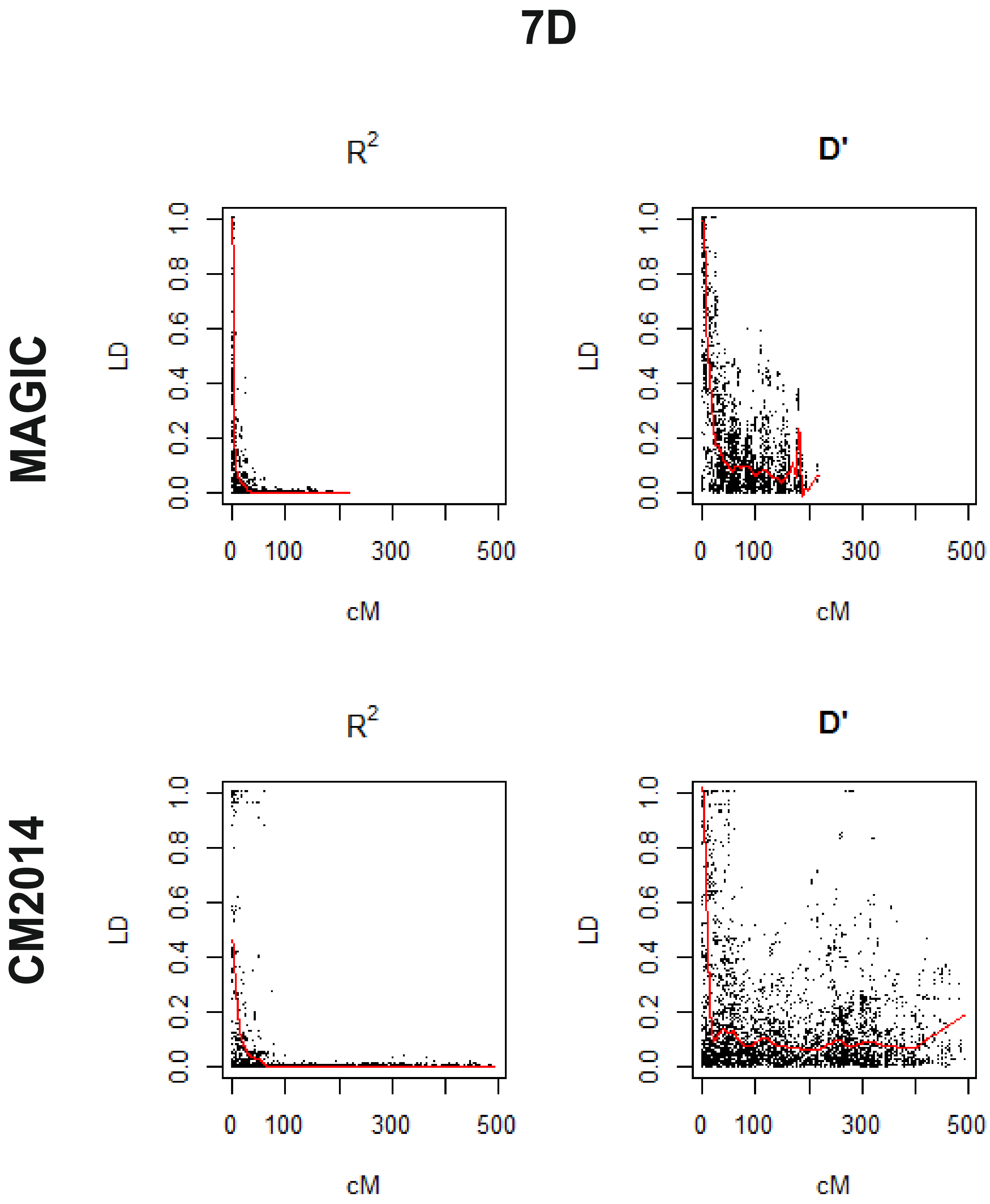
7D
MAGIC
CM2014
R2
D'
LD
cM
0.0
0.2
0.4
0.6
0.8
1.0
0
100
300
500
