# Supplementary material for: A highly recombined, high‐density, eight‐founder wheat MAGIC map reveals extensive segregation distortion and genomic locations of introgression segments
Source: Plant Biotechnol J. 2016 Jan 23;14(6):1406–17. doi: 10.1111/pbi.12504 (PMC4985697; doi:10.1111/pbi.12504)
Supplement: Supplementary file 6 — Appendix S1 Methods (Genotype calling including genotype error calculation, mapping error rate estimation, SD analysis). [file PBI-14-1406-s001.docx]

**Appendix S1**

**Genotyping**

MAGIC population construction and DNA extraction is described in Mackay et al., (2014). Genotyping was performed using the Illumina Infinium iSelect 80,000 SNP wheat array (“80K array”, http:// www.illumina.com/), provided as a service by the Department of Primary Industries (Victorian AgriBiosciences Center, Bundoora, VIC 3083, Australia). The development of this array is described in Wang et al. (2014). We successfully genotyped 707 lines of which 64 lines were dropped from the mapping analysis for showing a sporadic tendency to occur “off-cluster” towards other clusters, an indication of potential low level contamination. 20639 markers were scorable and polymorphic in the MAGIC population, compared to 25499 markers which were also polymorphic in a comprehensive UK wheat association mapping panel (520 varieties, using identical scoring criteria, Gardner et al. in prep), suggesting the MAGIC population has captured >80% of the genetic diversity of UK wheat germplasm. 53 markers had heterozygotes or missing data in the founder lines so could not be used for mapping with R/mpMap. Of the remaining 20586 markers, 18750 (91%) were scored as codominant and 1836 (9%) as dominant. 664 of the dominant loci were nulls (3.2%) compared to 5.4% of single-locus scorable SNPs showing null alleles in Wang et al (2014); we filtered out a disproportionate number of null allele loci based on SD. For codominant markers, 2.4% residual heterozygocity was observed, compared to an expectation of 2.2%. Four PCR markers were also genotyped for all or part of the MAGIC population, three of these by RAGT UK Ltd.

**Table of extra PCR markers**

| Marker name | Trait | Source | No individuals genotyped |
| --- | --- | --- | --- |
| *Ppd_D1* | Flowering time | Rhian Howells (NIAB) | 555 |
| OBM | **O**range wheat **B**lossom **M**idge resistance | RAGT | 506 |
| 1B_1R | 1B_1R rye translocation | RAGT | 507 |
| *Rht_D* | Height | RAGT | 507 |

Note that these 4 PCR markers were not subjected to the same QC procedure as the SNP array markers. Founder genotypes matched prior expectation for *Ppd_D1* (Soissons minority allele)*, Rht_*D (Robigus and Soissons) and 1B_1R (Brompton, Rialto), OBM was considered largely to have entered the UK wheat gene pool via Robigus but the phenotype was known also to occur in other varieties including Brompton (Phil Howells, pers. comm.). However, whether the resistance had one or more different genetic sources was unclear. Here the OBM marker was found in both Robigus and Brompton. All four PCR markers have previously been mapped and serve as a useful first test of our mapping accuracy. All four markers did indeed map to their expected locations in our map.

**SNP genotype calling**

As shown previously by Wang et al. (2014), using the standard diploid version of the Genome Studio (GS) software (llumina) for genotype calling in polyploid wheat has serious shortcomings. To address these shortcomings, Wang et al. (2014) developed a polyploid scoring approach in GS, using two density-based cluster identification algorithms in conjunction with a cluster file from multiple bi-parental populations. This represented a significant improvement over the diploid approach, and substantially reduced the amount of manual curation required. Unfortunately, the algorithms used in the polyploid clustering approach are limited in their ability to identify low-density clusters (Wang et al., 2014) and so are not well-matched to analysis of this MAGIC population, where most markers have a low density heterozygote cluster (average 15 heterozygotes across all markers). As a result, we used an alternative strategy as follows:

1. All SNP assays were called with the diploid calling algorithm in GS v2011.1, but using the wheat cluster file 130208_90kInfinium_SNPclusterfile_IWSC.egt, developed from Wang et al. (2014) (obtained from A. Allen, University of Bristol) and a no call threshold off 0.10.
2. Assays where founders were polymorphic, missing data were <5% of the total and the genotype frequency of the MAGIC lines was consistent with the founder frequencies (i.e. there was no evidence of segregation distortion, with a P value of <0.05), were put into bins based on the number of missing genotypes (0,1,2,3,4,5,6,7,8,9,10, >10).
3. A subsample of 100 assays per bin was scored manually and the error rate of the automated scoring was calculated. A cut-off of acceptable error rate was determined as 0 assays with incorrect calls >2%, 2 or fewer assays with incorrect calls <2%. This was found in bins with fewer than 4 missing data points. Automated SNP calls were therefore accepted for single marker assays with <4 missing data points and no segregation distortion.
4. Of the remaining loci, we discarded those that were putatively assigned to A or B genomes (as determined by BLASTn searches of the IWGSC wheat genome assembly <http://www.wheatgenome.org/>) AND which had no polymorphism or which had missing data>50%. However, we kept all markers putatively assigned to the D genome so as to potentially maximise the recovery of D genome markers within the time constraints of the project. All remaining data (putative D genome and A/B variable, NC<50%) were examined visually, and where clearly segregating for a locus, were manually curated. Loci exhibiting segregation distortion were accepted in the dataset if the visual observations were clear; if visually uncertain they were discarded. A scoring file was prepared based on the cut-off values of Theta and R which separated genotypes, enabling further samples and populations to be scored consistently in the future.
5. Most loci were scored as codominant but where heterozygotes were difficult to separate from one of the homozygotes (almost always the result of a difference in signal strength of the 2 alleles), loci were scored as dominant. The majority of loci segregating for null alleles were scored in this way due to difficulty separating heterozygotes from the non-null allele(s). Where greater than one locus was segregating in an assay, all scorable loci were used and identified by a suffix (a, b, c). For loci with 2 segregating alleles and a segregating null allele, the locus was scored in 2 separate ways: “a” suffix = NULL vs. allele, “b” suffix = codominant with NULL=NA. The latter could not be used in the map due to the constraint of no missing parental genotypes.

**Genotyping error rate estimation**

We estimate error rates at several stages of the genotyping process.

1. "Technical" replicates of the machine (identical samples being run more than once) - 0.00011 (0.011%).
2. “NA” in automatically and manually scored data combined - 0.0005 per data point (0.33 per marker)
3. Automatically-called data – rescoring checks on 350 markers – errors not called as NAs – 0.048 per marker (0.000075 per data point)
4. Manually-called data – rescoring checks on 300 markers - errors not called as NAs – 0.12 per marker (0.000264 per data point)

The overall error rate excluding “NA”s is therefore probably less than 0.3 per marker (0.00047 per data point) and including “NA”s about 0.61 per marker (0.00095 per data point).

**Physical map comparisons**

We assigned markers to contigs in both IWGSC and CHAPMAP at different stringency levels, and quantified differences in MAGIC map location for markers sharing contigs. With an e-value cut-off of 1e-10, 99% of mapped markers were assigned to a contig in both the IWGSC and CHAPMAP maps. However, 43% of markers were found in singleton contigs in IWGSC, compared to only 33% in CHAPMAP; this is consistent with the much greater number of scaffolds ≥10kb in CHAPMAP (Chapman et al., 2015). Increasing the stringency to 99% identity, only 66% of markers could be assigned to a contig in CHAPMAP, but this reduced to 46% in IWGSC. In Table 2, we presented the within contig analysis from the CHAPMAP map with high stringency only, while noting that the within contig analysis results are broadly similar in both IWGSC1 and CHAPMAP, and also between the stringent and none stringent datasets. A priori, we knew that our MAGIC population was segregating for the well-known 1BS rye introgression, that it was present in 2 of the MAGIC founders (Brompton, Rialto) and that when present, the introgression region does not recombine with other bread wheat chromosomes. Given its interspecific origins, there is a disproportionately very high density of markers segregating in this region, between the introgression and the native bread wheat chromosomes (>700 markers in complete LD). Large tracts of 1BS show high segregation distortion against the introgression and we knew from the heat map, PCA plot and map comparisons (Figure 2, supplementary material 4, 5) that there was a detrimental effect on our map accuracy on 1BS. Markers segregating within bread wheat chromosomes in the vicinity of the rye introgression will be pushed to the side of the markers which are only segregating between rye and bread wheat. We therefore anticipated that markers sharing contigs on 1B would be more likely to be non-contiguous than on other chromosomes and analysed 1B separately. Table 2 shows the results for all other chromosomes. For 1B, 75% of markers in shared contigs on the same chromosome were fully consistent with our map order (same or adjacent location), a further 5% had a single non-shared unique site (usually a single marker) between the shared contig markers and the remaining 20% were further apart in genetic distance (median 9.32cM).

**Genome diversity analysis**

For combined SD-HD blocks potentially representing interspecific introgressions in the founder Robigus, we made a detailed comparison to the Bristol University 820K SNP array database on cereals db (http://www.cerealsdb.uk.net). Firstly, we extracted all markers where the Robigus allele was found in some or all of 17 varieties with known Robigus pedigree but found in fewer than 10 out of 69 varieties with known pedigrees not including Robigus. We then blasted the flanking sequences of the SNP markers against the IWGSC genome sequence (IWGSC 2014) using BLASTn, with a cut-off of 98% identity and an e-value of 1e-20. This generated a dataset of 10169 SNPs, of which 7202 had a chromosomal location assigned in the IWGSC2 pseudomolecules. We searched this Robigus-enriched dataset to find all the markers corresponding to blocks of interest in our dataset. 481 (6.7%) and 595 (8.3%) of these respectively mapped to the 3B and 4A SD-HD blocks involving Robigus. We also generated a strict Robigus dataset with 0 of the 69 varieties with known non-Robigus pedigree having the Robigus allele. This dataset had 921 SNPs, of which 678 had a chromosomal location assigned in the IWGSC2 pseudomolecules. We arranged this dataset by chromosome, searched for continuous blocks of proximate (<0.75 Mb separation in IWGSC2 pseudomolecule) markers, and compared to our results. The distribution of block sizes was as follows:

| Block size | No blocks |
| --- | --- |
|  |  |
| 155 | 1 |
| 59 | 1 |
| 48 | 1 |
| 19 | 1 |
| 17 | 1 |
| 16 | 1 |
| 12 | 1 |
| 9 | 1 |
| 7 | 2 |
| 6 | 2 |
| 5 | 3 |
| 4 | 6 |
| 3 | 14 |
| 2 | 19 |
| 1 | 198 |

The largest block (155 markers) is a perfect match to the region of the distal end of 4AL containing our SD-HD linkage block with SD against Robigus. This is therefore a very strong candidate for the elusive Robigus-derived *Triticum dicoccoides* introgression in UK wheat.

**References**

Chapman, J.A., Mascher, M., Buluc, A., Barry, K., Georganas, E., Session, A., Strnadova, V., Jenkins, J., Sehgal, S., Oliker, L., Schmutz, J., Yelick, K.A., Scholz, U., Waugh, R., Poland, J.A., Muehlbauer, G.J., Stein, N. and Rokhsar, D.S. (2015) A whole-genome shotgun approach for assembling and anchoring the hexaploid bread wheat genome. *Genome Biol* **16**, 26.

International Wheat Genome Sequencing Consortium (2014) A chromosome-based draft sequence of the hexaploid bread wheat (Triticum aestivum) genome. *Science* **345**, 11.

Mackay, I.J., Bansept-Basler, P., Barber, T., Bentley, A.R., Cockram, J., Gosman, N., Greenland, A.J., Horsnell, R., Howells, R., O'Sullivan, D.M., Rose, G.A. and Howell, P.J. (2014) An Eight-Parent Multiparent Advanced Generation Inter-Cross Population for Winter-Sown Wheat: Creation, Properties, and Validation. *G3-Genes Genomes Genet.* **4**, 1603-1610.

Wang, S., Wong, D., Forrest, K., Allen, A., Chao, S., Huang, B.E., Maccaferri, M., Salvi, S., Milner, S.G., Cattivelli, L., Mastrangelo, A.M., Whan, A., Stephen, S., Barker, G., Wieseke, R., Plieske, J., International Wheat Genome Sequencing, C., Lillemo, M., Mather, D., Appels, R., Dolferus, R., Brown-Guedira, G., Korol, A., Akhunova, A.R., Feuillet, C., Salse, J., Morgante, M., Pozniak, C., Luo, M.C., Dvorak, J., Morell, M., Dubcovsky, J., Ganal, M., Tuberosa, R., Lawley, C., Mikoulitch, I., Cavanagh, C., Edwards, K.J., Hayden, M. and Akhunov, E. (2014) Characterization of polyploid wheat genomic diversity using a high-density 90,000 single nucleotide polymorphism array. *Plant Biotechnol J* **12**, 787-796.
